# An iron-regulated methionine redox axis governs adipose browning and cancer cachexia

**DOI:** 10.1038/s43018-026-01234-y

**Published:** 2026-09-22

**Authors:** Jung Seung Nam, Sung Shin Ahn, Yeonoh Shin, Ariana Vargas-Castillo, Maya S. Dixon, Pardis Ahmadi, Kathrin Schilling, Fereshteh Zandkarimi, Alex Chen, Nal Ae Yoon, Harrison Cullen, Yanping Sun, Thomas C. Caffrey, Kelsey A. Klute, Benjamin J. Swanson, Jordan Lu, Samuel Pan, Yuxuan Chen, Shu Ichimiya, Geena Kim, Jeanine M. Genkinger, Kazuki Sugahara, Michael D. Kluger, Paul Grandgenett, Michael A. Hollingsworth, Luke Berchowitz, Anthony W. Ferrante, Lori M. Zeltser, Sabrina Diano, Iok In Christine Chio

**Affiliations:** 1 Institute for Cancer Genetics, Columbia University Irving Medical Center, New York, NY USA; 2 Herbert Irving Comprehensive Cancer Center, Columbia University Irving Medical Center, New York, NY USA; 3 Department of Biochemistry and Molecular Biophysics, Columbia University Irving Medical Center, New York, NY USA; 4 Department of Cell Biology, Harvard Medical School, Boston, MA USA; 5 Department of Biomedical Engineering, Columbia University, New York, NY USA; 6 Department of Environmental Health and Sciences, Columbia University Irving Medical Center, New York, NY USA; 7 Institute of Human Nutrition, Columbia University Irving Medical Center, New York, NY USA; 8 Department of Physiology and Cellular Biophysics, Columbia University Irving Medical Center, New York, NY USA; 9 Department of Genetics and Development, Columbia University Irving Medical Center, New York, NY USA; 10 Eppley Institute for Research in Cancer and Allied Diseases, Fred and Pamela Buffett Cancer Center, University of Nebraska Medical Center, Omaha, NE USA; 11 Department of Internal Medicine, University of Nebraska Medical Center, Omaha, NE USA; 12 Department of Pathology and Microbiology, College of Medicine, University of Nebraska Medical Center, Omaha, NE USA; 13 Department of Biostatistics, Columbia University Irving Medical Center, New York, NY USA; 14 Department of Epidemiology, Columbia University Irving Medical Center, New York, NY USA; 15 Division of Gastrointestinal and Endocrine Surgery, Vagelos College of Physicians and Surgeons, Columbia University Irving Medical Center, New York, NY USA; 16 Department of Medicine, Columbia University Irving Medical Center, New York, NY USA; 17 Department of Pathology and Cell Biology, Columbia University Irving Medical Center, New York, NY USA

**Keywords:** Cancer metabolism, Post-translational modifications

## Abstract

Adipose browning and atrophy are early events of cachexia, a lethal metabolic disorder affecting nearly half of the population with cancer. Here, using individual-derived specimens and mouse models, we identified an iron-dependent pathway that initiates adipose browning in both physiological and cachectic settings. Upon adrenergic stimulation of adipocytes, an influx of iron induces the activity of methionine sulfoxide reductase A (MSRA), an enzyme that reverses the oxidation of proteinaceous methionine residues. Mechanistically, iron coordination by the conserved iron-binding EXXH motif of two MSRA polypeptides serves to dimerize, stabilize and elevate its reductase activity. Iron-bound MSRA dimers in turn promote adipose browning by maintaining the reduced state of select substrates, including the catalytic subunit of protein kinase A. Remarkably, in mouse models, *MsrA* deletion impairs adipose browning, mitigates cachexia and prolongs the survival of tumor-bearing animals. Thus, as a key nexus of cancer-associated cachexia, the β3 adrenergic receptor–iron–MSRA axis is a promising target for clinical intervention.

## Abstract (web summary)

Chio and colleagues describe an iron-dependent pathway with a role in the induction of cancer cachexia-linked events such as adipose browning and identify methionine sulfoxide reductase A as an important and targetable factor in this process.

## Main

Individuals with cancer who develop cachexia, a metabolic disorder characterized by morbid wasting of adipose tissue and skeletal muscle, suffer involuntary weight loss, poor responses to therapy and elevated mortality rates^1^. While cachexia affects nearly half of the population with cancer, it is especially prevalent among those with pancreatic ductal adenocarcinoma (PDA), the most lethal pancreatic malignancy^2^. Although the biological factors that induce cachexia remain poorly defined, recent studies indicate that adipose tissue wasting precedes muscle loss in individuals with cancer^3–5^ and that muscle mass can be preserved in tumor-bearing animals by experimental blockage of adipose depletion^6,7^. Thus, elucidating the factors that initiate adipose loss should reveal underlying mechanisms of cancer cachexia and provide new clinical strategies to forestall its development.

Mammals have both white adipose tissue (WAT), which stores energy as triglycerides, and brown adipose tissue (BAT), which can release energy as heat. WAT, the predominant type of fat tissue in adults, is composed mostly of white adipocytes that have a unilocular morphology typically marked by a single massive lipid droplet. With relatively few mitochondria, these cells have limited capacity for oxidative phosphorylation. In contrast, the brown adipocytes of BAT, which possess many small (multilocular) lipid droplets and abundant mitochondria, can robustly support oxidative phosphorylation. Under certain physiological conditions, such as cold exposure, white adipocytes can be transformed into ‘beige’ adipocytes that have BAT-like features, including a multilocular morphology and high mitochondrial content^8^. As a result of this ‘browning’ process, beige adipocytes gain a thermogenic potential that is readily induced by sympathetic tone. In particular, the norepinephrine released by parenchymal neurites can activate β3 adrenergic signaling in beige adipocytes, mobilizing their lipid stores for thermogenesis^9–12^.

As the fat depots of adult humans are predominantly comprised of white adipocytes, WAT browning is often associated with metabolic health and the mitigation of obesity^13^. However, when dysregulated, sustained thermogenic remodeling can drive pathological energy expenditure and systemic wasting^14^. Indeed, WAT browning has been shown to be an early hallmark of cancer cachexia that accompanies adipose atrophy and precedes skeletal muscle wasting in both cancer mouse models^7,15–17^ and patients^5,18,19^. Here, we describe an adrenergic iron-dependent signaling axis (β3 adrenergic receptor (β3AR)–iron–methionine sulfoxide reductase A (MSRA)) that promotes WAT browning by preserving the proper redox state of select methionine residues in the adipocyte proteome. This axis is required for browning in both pathological (for example, cancer cachexia) and physiological (cold-induced thermogenesis) settings and its abrogation can alleviate cachexia and prolong the survival of tumor-bearing animals.

## Methionine oxidation is altered during WAT browning in PDA-induced cachexia

**Fig. 1:**
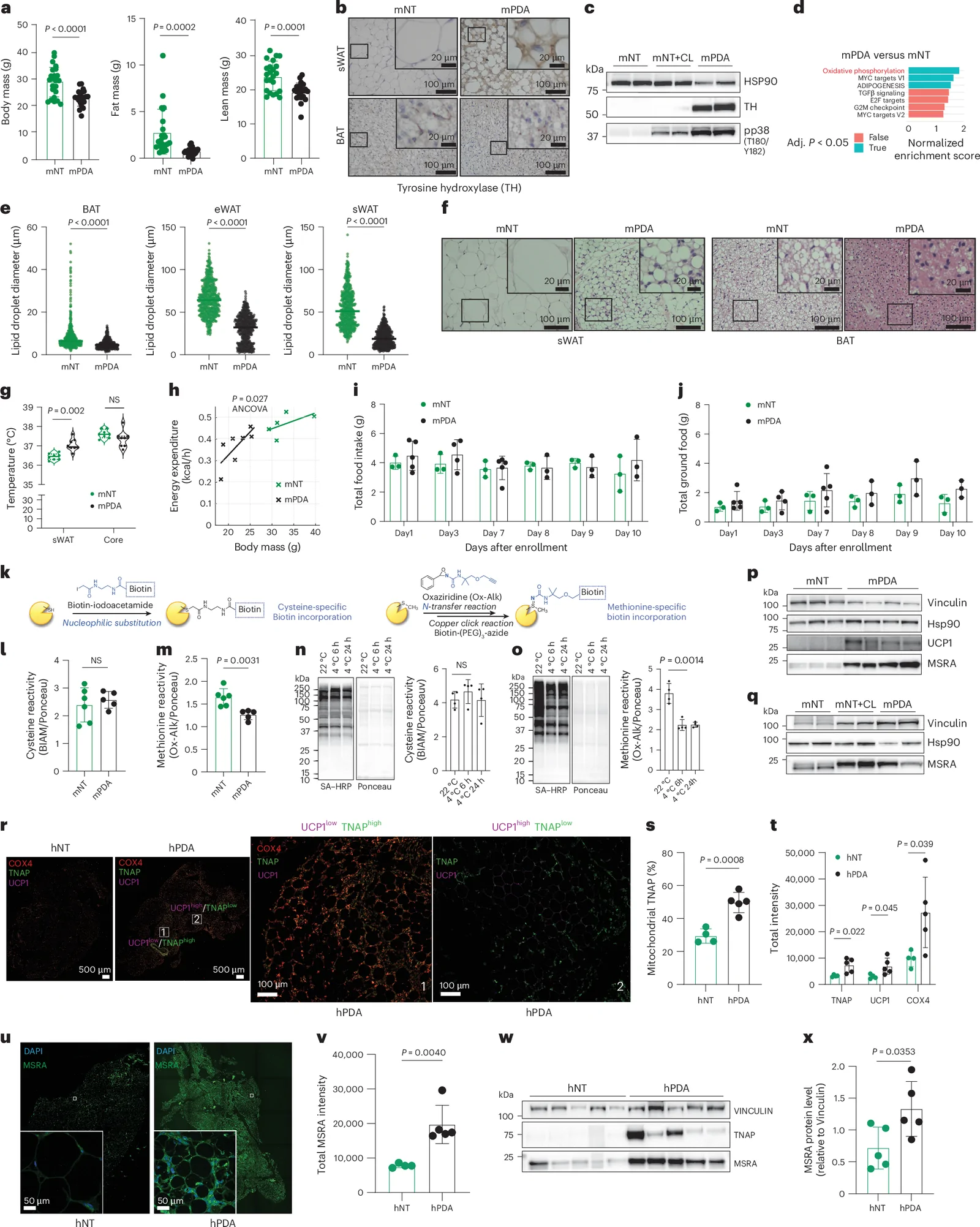
MSRA is markedly induced in WAT during cancer-induced cachexia. **a**, Body, fat and lean mass of mPDA mice compared to age-matched (3–8 months old) mNT controls. mNT, *n* = 23 mice (16 males and 7 females); mPDA, *n* = 24 mice (7 males and 17 females). **b**, Representative immunohistochemical staining of TH expression level in BAT and sWAT. The square box is enlarged to the same magnification in the top right corner of each image. Representative images are shown from three independent mice per group with similar results. **c**, Immunoblot analysis of sWAT. mNT mice treated with 5 mg kg^−1^ β3-agonist CL-316243 (mNT + CL) are included as a control for β3AR activation. Each lane represents lysate from an independent mouse. mNT, *n* = 2 mice; mNT + CL, *n* = 2 mice; mPDA, *n* = 2 mice. **d**, Gene set enrichment analysis of the global sWAT proteome. Adjusted *P* value was determined using Benjamini–Hochberg correction. **e**, Lipid droplet diameter measurements of BAT, eWAT and sWAT from mPDA and mNT controls. A total of 100 lipid droplets were quantified from each adipose tissue specimen (BAT, *n* = 5 mice; sWAT, *n* = 6 mice; eWAT, *n* = 6 mice). **f**, Representative H&E staining of sWAT and BAT. The square box is enlarged to the same magnification in the top right corner of each image. Representative images are shown from independent mice with similar results (BAT, *n* = 5 mice; sWAT, *n* = 6 mice). **g**, Inguinal sWAT temperature measured with implanted temperature transponders and core temperature measured with a rectal probe. mNT, *n* = 8 mice; mPDA, *n* = 8 mice. **h**, Mean energy expenditure of each animal plotted as a function of their body mass. An analysis of covariance (ANCOVA) was performed to assess the energy expenditure–body mass relationship of mPDA mice compared to mNT controls (mPDA slope = 0.0215 kcal h^−1^ g^−1^; mNT slope = 0.0073 kcal h^−1^ g^−1^). The *P* value was derived from an ANCOVA. mNT, *n* = 5 mice; mPDA, *n* = 7 mice. **i**,**j**, As food intake in the mPDA group (Extended Data Fig. 1h) was higher than the expected daily intake of 3–5 g per day for an adult mouse^74^, we performed an extended measurement of food intake (**i**) and food grinding (**j**) behavior, which may be increased by stress^75^. Manual measurement of ground food and food consumption was conducted by weighing the daily differences in chow pellets for each singly housed mouse during their waking hours (at 6 p.m. and 9 a.m.) over a 10-day period. Actual food intake (**i**) was calculated by subtracting the mass of ground food on the cage floor. Three mPDA mice died after day 7. There was an increase in food grinding by mPDA mice, albeit not statistically significant. mNT, *n* = 3 mice; mPDA, *n* = 5 mice. **k**, Scheme of global cysteine and methionine oxidation analysis using BIAM and Ox-Alk. **l**,**m**, Global cysteine oxidation analysis by BIAM (**l**) and global methionine oxidation analysis by Ox-Alk (**m**) of the sWAT proteome. mNT, *n* = 6 mice; mPDA, *n* = 5 mice. **n**, Global cysteine oxidation analysis by BIAM of the sWAT proteome upon acute cold exposure (*n* = 4 mice each). **o**, Global methionine oxidation analysis by Ox-Alk of the sWAT proteome upon acute cold exposure (*n* = 4 mice each). **p**,**q**, Immunoblot analysis of sWAT lysates for MSRA expression. Hsp90 and Vinculin were used as loading controls; UCP1 was used as a marker of adipose browning. mNT mice treated with 5 mg kg^−1^ CL-316243 (mNT + CL) are included as a control for β3AR activation. Each lane represents lysate from an independent mouse. mNT, *n* = 3 mice; mPDA, *n* = 4 mice (**p**); mNT, *n* = 2 mice; mNT + CL, *n* = 2 mice; mPDA, *n* = 2 mice (**q**). **r**–**v**, Analysis of WAT from hNT (*n* = 4) or hPDA (*n* = 5) participants: immunofluorescence staining (**r**) and quantification of mitochondrial TNAP abundance (**s**), TNAP, UCP1 and COX4 levels (**t**), and immunofluorescence staining (**u**) and quantification of MSRA levels (**v**). Square white boxes demarcate enlarged regions. DAPI, nuclear marker. Each data point is the total intensity of the entire section from one participant. Participant information is detailed in Supplementary Table 2. **w**,**x**, Immunoblot analysis (**w**) and quantification (**x**) of individual-derived WAT. Each lane represents lysate from an independent participant. hNT, *n* = 5 participants; hPDA, *n* = 5 participants. Error bars represent the mean ± s.d. A two-tailed unpaired Student’s *t*-test was performed unless stated otherwise. NS, not significant. Source data

**Extended Data Fig. 1:**
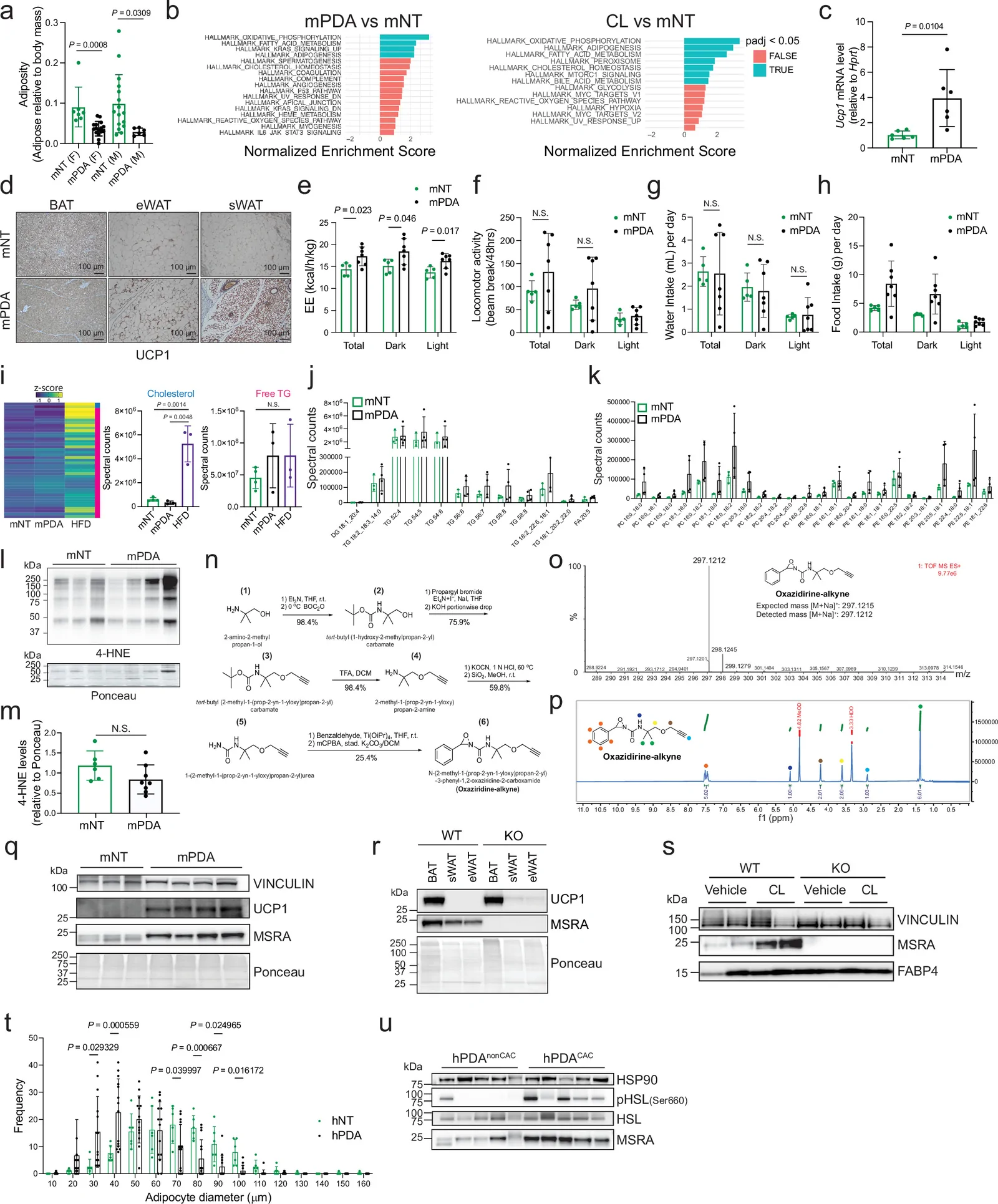
Cachectic and physiological adipose browning are associated with hypermetabolism, methionine oxidation, and MSRA induction. **a**, Normalized fat mass of pancreatic tumour-bearing mice (mPDA) compared to age-matched non-tumour controls (mNT). F, female; M, male. mNT (F), *n* = 7 mice; mPDA (F), *n* = 17 mice; mNT (M), *n* = 16 mice; mPDA (M), *n* = 6 mice. **b**, Gene Set Enrichment Analysis of the sWAT proteome from female pancreatic tumour-bearing mice (mPDA) compared to age-matched non-tumour controls (mNT) (left), and non-tumour-bearing male mice treated with 5 mg/kg β3-adrenergic agonist CL-316243 (CL) compared to non-tumour controls (mNT) for 6 days (right). Adjusted *P* value was determined using Benjamini-Hochberg correction. **c**, qRT-PCR of *Ucp1* mRNA expression in eWAT of pancreatic tumour-bearing mice (mPDA) compared to age-matched non-tumour controls (mNT). mNT, *n* = 6 mice; mPDA, *n* = 6 mice. **d**, Immunohistochemical analysis of UCP1 expression in BAT and WAT from pancreatic tumour-bearing mice (mPDA) and age-matched non-tumour controls (mNT). Representative images are shown from independent mice per group with similar results (mNT, *n* = 5 mice; mPDA, *n* = 4 mice). mNT and mPDA are the same animals in Figs. 5l and 5m. **e**, Energy expenditure (EE) per hour normalized to body weight. mNT, *n* = 5 mice; mPDA, *n* = 7 mice. **f-h**, Locomotor activity (**f**), water (**g**), and food intake (**h**, Total: *P* = 0.03, Dark: *P* = 0.04, Light: *P* = 0.09) of pancreatic tumour-bearing mice (mPDA) compared to age-matched non-tumour controls (mNT) over a four-day period in metabolic changes. Welch’s *t*-test was performed, given a significant variation in the variance of locomotive activity and food intakes of tumour-bearing mice compared to non-tumour controls (F-test, *P* < 0.01 for each parameter). *P* > 0.05 for all comparisons unless stated otherwise. mNT, *n* = 5 mice; mPDA, *n* = 7 mice. **i**, To rule out caloric intake-related confounders, we monitored fecal lipid content, as malabsorption can be a symptom of pancreatic cancer^1^, by performing lipidomic analysis of cholesterol and triglyceride abundance in feces. Global abundance is presented as a heatmap while specific species are plotted as accompanying bar graphs. sWAT from animals fed a high-fat diet (60% fat) for 5 weeks was included as a positive control for steatohorea. mNT, *n* = 4 mice; mPDA, *n* = 3 mice; High fat diet (HFD), *n* = 3 mice. **j**,**k**, Lipidomic analysis of the abundance of PUFAs with triglyceride (**j**) and phospholipid (**k**) headgroups in sWAT. mNT, *n* = 3 mice; mPDA, *n* = 4 mice. **l**,**m**, Immunoblot analysis (**l**) and quantification (**m**) of 4-HNE-modified proteins in sWAT of pancreatic tumour-bearing mice (mPDA) compared to age-matched non-tumour controls (mNT). mNT, *n* = 6 mice; mPDA, *n* = 8 mice. **n**, Chemical synthesis scheme for oxaziridine-alkyne (Ox-Alk). **o**,**p**, Synthesis product validation by high-resolution mass spectrometry (**o**) and proton nuclear magnetic resonance spectroscopy (^1^H-NMR) (**p**). ^1^H NMR (400 MHz, MeOD): δ (ppm) = 7.55–7.40 (*m*, 5H), 5.09 (*s*, 1H), 4.22 (*t*, 2H, *J* = 1.6 Hz), 3.61 (*s*, 2H), 2.89 (1H), 1.38 (*s*, 6H). HR-MS: calcd. for C_15_H_18_O_3_N_2_Na [M+Na]^+^: 297.1215; measured: 297.1208. **q**, Immunoblot analysis of protein expression in eWAT of pancreatic tumour-bearing mice (mPDA) compared to age-matched non-tumour controls (mNT). Each lane represents lysate from an independent mouse. mNT, *n* = 3 mice; mPDA, *n* = 4 mice. **r**, Immunoblot analysis of protein expression in BAT, eWAT, and sWAT from non-tumour-bearing mice. WT, *MsrA*^*+/+*^; KO, *MsrA*^−*/*−^. Images are representative of two independent experiments with similar results. **s**, Immunoblot analysis of protein expression in sWAT from male non-tumour-bearing mice treated with 5 mg/kg β3-adrenergic agonist CL-316243 (CL) or vehicle for 6 days. Each lane represents lysate from an independent mouse. WT vehicle, *n* = 2 mice; WT CL, *n* = 2 mice; KO vehicle, *n* = 2 mice; KO CL, *n* = 2 mice. **t**, Adipocyte diameter of white adipose tissues from non-tumour patients who underwent laparoscopic cholecystectomy (hNT) or were diagnosed with cachexic PDA (hPDA). 100 adipocytes were measured per patient specimen. Means ± SEMs. Each dot is one patient. hNT, *n* = 6 patients; hPDA, *n* = 14 patients. **u**, Immunoblot analysis of WAT from pancreatic cancer patients with (hPDA^CAC^, body mass loss >5%) or without (hPDA^nonCAC^, no or < 5% body mass loss) cachexia. Each lane represents lysate from an independent patient. hPDAC^nonCAC^, *n* = 5 patients; hPDAC^CAC^, *n* = 5 patients). Error bars in this Fig. are means ± SDs unless stated otherwise. A two-tailed unpaired Student’s *t*-test was performed unless stated otherwise. N.S., not significant. 1. Bartel, M. J., Asbun, H., Stauffer, J. & Raimondo, M. Pancreatic exocrine insufficiency in pancreatic cancer: A review of the literature. *Dig Liver Dis* **47**, 1013–1020 (2015). Source data

Previous studies have shown that PDA-bearing mice develop a cachectic phenotype marked by profound loss of adipose and muscle tissue^20,21^. Thus, to identify factors that induce adipose browning during cancer cachexia, we used the autochthonous KPC (*Kras*^G12D^;*p53*^R172H^;*PdxCre*)^22^ mouse model of PDA. As expected^20,21^, PDA-bearing KPC mice (mPDA) with tumors of 5–7 mm diameter lost significant weight, with depletion of both adipose and lean mass, relative to age-matched nontumor wild-type (WT, mNT) controls (Fig. 1a). Adipose atrophy occurred to the same degree in male and female mice upon adjusting for body mass differences (Extended Data Fig. 1a).

Interestingly, WAT of mPDA mice expresses elevated levels of tyrosine hydroxylase (TH), a marker of adipose sympathetic innervation^23^ (Fig. 1b,c). Global proteomic analysis of subcutaneous WAT (sWAT) (Supplementary Table 1) further revealed a marked upregulation of proteins involved in oxidative phosphorylation in both male (Fig. 1d) and female (Extended Data Fig. 1b) mPDA mice relative to mNT controls. A similar induction of oxidative phosphorylation occurred in nontumor animals treated with the β3AR agonist CL-316243, which mimics physiological induction of WAT browning (Extended Data Fig. 1b). Likewise, activated p38 kinase, a central regulator of thermogenesis^24^, was induced in the sWAT of both PDA-bearing animals and CL-316243-treated nontumor animals (Fig. 1c). In addition, white adipocytes from mPDA mice contain smaller lipid droplets, exhibit a BAT-like multilocular morphology and show elevated expression of UCP1, a mitochondrial uncoupling protein that mediates thermogenesis^9^ (Fig. 1e,f and Extended Data Fig. 1c,d). In normal (that is, nontumor) mice, UCP1-mediated energy expenditure occurs in response to specific physiological stimuli, such as cold exposure^8^. Interestingly, mPDA mice maintained at ambient temperature generated greater diurnal heat within their sWAT depots relative to age-matched mNT controls (Fig. 1g). These mice also displayed an elevated diurnal energy expenditure (Extended Data Fig. 1e) and a disproportionate increase in energy expenditure per unit of body mass (Fig. 1h). This hypermetabolic state was not associated with changes in core body temperature (Fig. 1g), activity (Extended Data Fig. 1f), water intake (Extended Data Fig. 1g), food intake (Fig. 1i and Extended Data Fig. 1h), food grinding (Fig. 1j) or malabsorption (Extended Data Fig. 1i). Together, these findings indicate that early-stage PDA-bearing mice undergo hypermetabolic adipose remodeling associated with increased heat dissipation and negative energy balance.

As mitochondrial respiration generates reactive oxygen species (ROS)^25^, we expected that the intracellular oxidation state of WAT would be increased in PDA-bearing mice relative to controls and adipose atrophy might reflect aberrant induction of ferroptosis. However, PDA WAT did not display characteristic indicators of lipid oxidation^26,27^, such as specific depletion of phospholipids or triglycerides with polyunsaturated fatty acid (PUFA) tails (Extended Data Fig. 1j,k) or increased lipid peroxide adduction (that is, 4-hydroxynonenal (4-HNE) abundance) (Extended Data Fig. 1l,m). Thus, ferroptosis is an unlikely outcome of cachectic WAT browning and atrophy. We next surveyed the sWAT proteome for major oxidative protein modifications, namely, cysteine oxidation (loss of biotinylated iodoacetamide (BIAM) reactivity; Fig. 1k,l) and methionine oxidation (loss of oxaziridine-alkyne (Ox-Alk) reactivity; Fig. 1k,m and Extended Data Fig. 1n–p). Of these, only methionine reactivity was significantly perturbed in the tumor setting (Fig. 1m), suggesting that oxidation of proteinaceous methionines may contribute to adipose wasting during cancer cachexia. Interestingly, the sWAT proteome also displayed decreased Ox-Alk but not BIAM reactivity in normal mice subjected to acute cold exposure (Fig. 1n,o). This observation implies that global methionine oxidation is a common feature of adipose browning during both cancer cachexia and physiological thermogenesis.

## MSRA induction occurs during both physiological and cachectic adipose browning

The sulfur atom of some proteinaceous methionines can act as a functional redox switch that toggles between its reduced and oxidized states^28,29^. This toggling occurs because ROS can convert methionine residues to their oxidized form, methionine sulfoxide, which in turn can be enzymatically reduced by MSRA^30^. Interestingly, this oxidoreductase was strongly upregulated in both the sWAT (Fig. 1p) and the epididymal WAT (eWAT) (Extended Data Fig. 1q) of tumor-bearing KPC mice. MSRA expression was also elevated, albeit more modestly, in the BAT compared to WAT depots of healthy mice (Extended Data Fig. 1r). In addition, MSRA could be robustly induced in the WAT of healthy mice by treatment with the β3AR-selective agonist CL-316243 (Fig. 1q and Extended Data Fig. 1s), suggesting that MSRA expression in adipocytes is also responsive to sympathetic stimulation.

To ascertain whether similar metabolic changes occur during human cachexia, we examined a panel of visceral adipose tissues procured from (1) nontumor control patients undergoing laparoscopic cholecystectomy (hNT) and (2) PDA patients with cachexia (>5% weight loss, hPDA) (Supplementary Table 2). The WAT adipocytes of PDA patients displayed smaller diameters (Extended Data Fig. 1t), a multilocular morphology (Fig. 1r), increased mitochondria, regionally increased UCP1 levels (Fig. 1r–t) and induction of mitochondrial TNAP (Fig. 1s,t), a key regulator of UCP1-independent thermogenesis^31^ in humans^32,33^. Increased MSRA expression (Fig. 1u–x and Extended Data Fig. 1u) and activating phosphorylation of the lipolytic enzyme hormone-sensitive lipase (HSL) (Extended Data Fig. 1u) were also observed in WAT specimens from cachectic PDA patients. Thus, MSRA expression is upregulated in the WAT of PDA patients and PDA-bearing mice, along with a metabolic shift toward mitochondrial respiration that occurs during WAT browning.

## Iron uptake stabilizes MSRA protein and induces adipose browning during cancer cachexia

**Extended Data Fig. 2:**
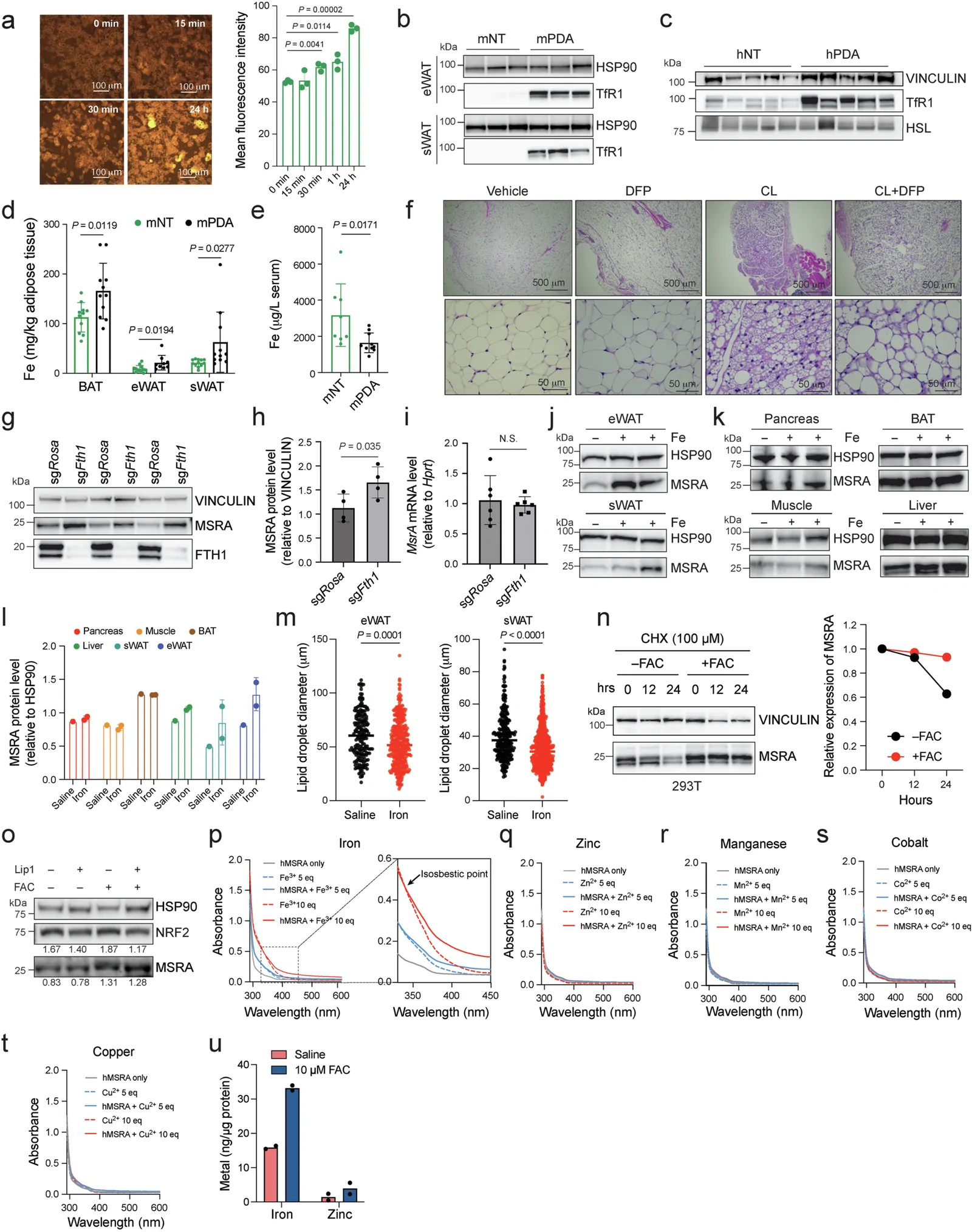
Iron uptake promotes MSRA stabilization and adipose browning. **a**, FerroOrange staining and quantification of white adipocyte cultures upon isoproterenol treatment at the indicated times. Images are representative of three independent experiments with similar results. Each dot represents an independent cell culture replicate (*n* = 3). **b**, Immunoblot analysis of protein expression in eWAT and sWAT from pancreatic tumour-bearing mice (mPDA) compared to age-matched non-tumour controls (mNT). Each lane represents lysate from an independent mouse. mNT, *n* = 3 mice; mPDA, *n* = 3 mice. **c**, Immunoblot analysis of protein expression in WAT from non-tumour patients who underwent laparoscopic cholecystectomy (hNT) or were diagnosed with cachexic PDA (hPDA). Each lane represents lysate from an independent patient. hNT, *n* = 5 patients; hPDA, *n* = 5 patients. **d**, Total iron (Fe) levels in the adipose tissues of pancreatic tumour-bearing mice (mPDA) compared to age-matched non-tumour controls (mNT). mNT (BAT, *n* = 11 mice; eWAT, *n* = 12 mice; sWAT, *n* = 12 mice) and mPDA (BAT, *n* = 11 mice; eWAT, *n* = 9 mice; sWAT, *n* = 11 mice). **e**, Total iron (Fe) levels in the serum of pancreatic tumour-bearing mice (mPDA) compared to age-matched non-tumour controls (mNT). mNT, *n* = 8 mice; mPDA, *n* = 10 mice. **f**, Representative hematoxylin and eosin (H&E) staining of subcutaneous white (sWAT) adipose tissues from mice treated with 5 mg/kg β3-adrenergic agonist (CL-316243), 50 mg/kg deferiprone (DFP), or in combination (CL + DFP) for 6 days. Images are representative of three independent mice with similar results (*n* = 3 mice each). **g**-**i**, Immunoblot (**g**, **h**) and qRT-PCR (**i**) analyses of white adipocyte cultures expressing control (sg*Rosa*) or ferritin heavy chain-specific (sg*Fth1*) sgRNAs. Each dot represents an independent cell culture replicate of primary adipocytes derived from neonates of each genotype (g-n; *n* = 4; i, *n* = 6). **j**-**l**, Immunoblot analysis (**j**, **k**) and quantification (**l**) of tissues 24 hours after receiving saline or three doses of iron dextran at 15 mg kg^−1^ spaced two hours apart. Each lane represents lysate from an independent mouse. Saline, *n* = 1 mouse; iron dextran, *n* = 2 mice. **m**, Lipid droplet diameter measurement of white adipose tissues 24 hours after receiving saline or three doses of iron dextran at 15 mg kg^−1^ spaced two hours apart. 200 lipid droplets were quantified from each adipose tissue specimen. Saline, *n* = 1 mouse; iron dextran, *n* = 2 mice. **n**, Cycloheximide (CHX) pulse chase of 293 T cells in the presence or absence of 10 μM ferric ammonium citrate (FAC). Images are representative of three independent experiments with similar results. **o**, Immunoblot analysis of MSRA expression in white adipocyte cultures upon treatment with FAC (10 μM) and the ferroptosis inhibitor Liproxstatin-1 (Lip1, 0.5 μM) for 24 hours. Images are representative of three independent experiments and show similar MSRA expression patterns. **p**-**t**, Absorbance properties of recombinant human MSRA in the presence of different elements. Inset, zoomed-in spectra of MSRA in the presence of ferric ammonium citrate (FAC). 5 eq, five molar equivalents in concentration. 10 eq, ten molar equivalents in concentration. Isosbestic point: A point observed in overlaid spectra when a chromophoric precursor is converted to a product with a different spectrum. An isosbestic point occurs only when the precursor is quantitatively converted to a single product. **u**, Inductively coupled plasma mass spectrometric analysis of total iron and zinc content in MSRA immunoprecipitants in the presence or absence of exogenous ferric ammonium citrate (FAC). Each dot represents an independent cell culture replicate (*n* = 2). Error bars in this Fig. are means ± SDs. Student’s *t*-test was performed. N.S., not significant. Source data

The characteristic brown color of thermogenic adipocytes is derived from the high-density iron–porphyrin complexes of their mitochondrial electron transport chains^34^. To meet the increased demand for oxidative metabolism in brown and beige adipocytes, β3AR signaling promotes iron uptake for synthesis of iron-bound mitochondrial enzymes^35^. As this physiological influx of iron is facilitated by upregulation of the transferrin receptor (TfR1), genetic disruption of *TfR1* impairs mitochondrial biogenesis and thermogenesis in brown and beige adipocytes^35^. Accordingly, β3AR stimulation of cultured WAT cells from WT neonates led to an increase in intracellular labile iron, as visualized using the ferrous iron-sensitive fluorescent probe FerroOrange (Extended Data Fig. 2a). We also observed markedly increased TfR1 levels in the WAT of both mPDA mice (Extended Data Fig. 2b) and cachectic PDA patients (hPDA) (Extended Data Fig. 2c). Moreover, inductively coupled plasma mass spectrometry (ICP–MS) revealed elevated levels of total iron content in the adipose depots of tumor-bearing mice (Extended Data Fig. 2d) and correspondingly reduced levels in their sera (Extended Data Fig. 2e). These observations suggest that iron influx not only is essential for physiological thermogenesis^35^ but also facilitates adipose browning during cancer cachexia.

**Fig. 2:**
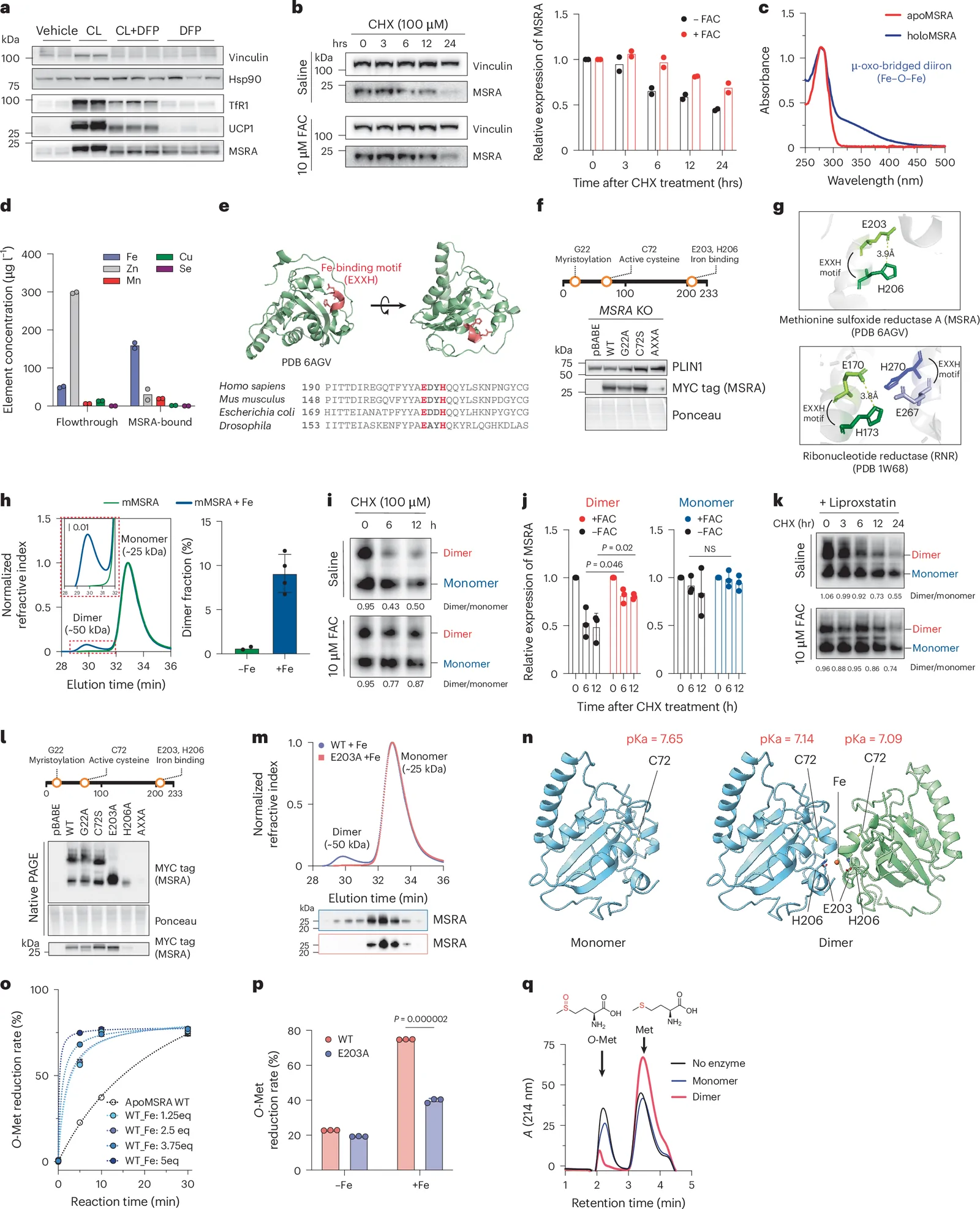
MSRA is stabilized and activated upon iron binding. **a**, Immunoblot analysis of sWAT from mNT mice treated with vehicle, 5 mg kg^−1^ β3AR agonist (CL-316243), 50 mg kg^−1^ DFP or their combination (CL + DFP) for 6 days. Hsp90 and Vinculin were used as loading controls. Each lane represents lysate from an independent mouse. Vehicle, *n* = 2 mice; CL, *n* = 2 mice; CL + DFP, *n* = 3 mice; DFP, *n* = 3 mice. **b**, Cycloheximide (CHX) pulse–chase to measure MSRA protein half-life in white adipocyte cultures treated with saline or FAC. Right: quantification of MSRA expression relative to Vinculin. Each dot represents an independent cell culture replicate (*n* = 2). **c**, UV–visible spectroscopy of recombinant murine MSRA absorbance with (holo) or without (apo) ferrous iron reconstitution in anoxia. **d**, ICP–MS was used to measure intracellular trace elements enriched in MSRA immunoprecipitants. Each dot represents an independent cell culture replicate (*n* = 2). **e**, *M*. *musculus* MSRA crystal structure and sequence alignment. Red, putative iron-binding motif. **f**, Immunoblot analysis of WT and various point mutants of MSRA in white adipocyte cultures. Data are representative of three independent experiments with similar results. **g**, Structural comparison of RNR and MSRA iron-binding sites. **h**, SEC–MALS analysis of monomer and dimer distribution of recombinant murine MSRA in the presence or absence of five molar equivalents of iron (pooled results from independent experiments: mMSRA, *n* = 2; mMSRA + Fe, *n* = 4). **i**,**j**, Native PAGE analysis (**i**) and quantification (**j**) of MSRA protein half-life in white adipocytes upon CHX treatment, in the presence of saline or 10 μM FAC. Data are representative of three independent experiments with similar results and each dot represents an independent cell culture replicate. **k**, Native PAGE analysis of MSRA protein half-life in 293T cells pretreated with 1 μM liproxstatin 1, followed by CHX, in the presence of saline or 10 μM FAC. Data are representative of two independent experiments with similar results. **l**, Native and SDS–PAGE analysis of WT and point mutants of MSRA. Data are representative of three independent experiments with similar results. **m**, SEC–MALS and immunoblot analysis of monomer and dimer distribution of recombinant WT and E203A mutant MSRA in the presence of five molar equivalents of iron. Data are representative of two independent experiments with similar results. **n**, Predicted iron-binding model of MSRA dimer, along with the respective active-site cysteine pKa in monomeric versus dimeric configurations. **o**, Reductase activity of recombinant WT MSRA measured by the conversion of oxidized to reduced methionine upon increasing concentration of iron over 30 min. **p**, Reductase activity of recombinant WT and E203A mutant MSRA measured by the conversion of oxidized to reduced methionine in the presence or absence of five molar equivalents of iron (pooled results from independent experiments: *n* = 3). **q**, Enzymatic activity of gel-filtration-purified recombinant His-tagged MSRA monomer and dimer when incubated with an equimolar ratio of oxidized and reduced methionine for 30 min. Error bars represent the mean ± s.d. A two-tailed unpaired Student’s *t*-test was performed. *A*, absorbance at 214 nm measured by the HPLC detector. Source data

To determine whether iron is required for MSRA induction during physiological browning, we coadministered healthy mice with CL-316243 and the iron chelator deferiprone (DFP). As expected^35^, iron chelation mitigated the browning effects of CL-316243 at the histological level (Extended Data Fig. 2f). In addition, the robust induction of MSRA expression that occurred in these cells upon β3AR activation and coincided with both TfR1 and UCP1 upregulation was reversed by DFP treatment (Fig. 2a). Conversely, expansion of the labile iron pool by CRISPR-mediated depletion of the iron storage protein ferritin heavy chain (FTH1) allowed post-transcriptional MSRA induction to occur in WAT adipocytes even in the absence of adrenergic stimulation (Extended Data Fig. 2g–i). Similarly, iron–dextran infusion in WT mice effectively induced MSRA expression in WAT (Extended Data Fig. 2j–l). Of note, iron administration alone also led to a modest but significant reduction in the diameter of eWAT and sWAT lipid droplets (Extended Data Fig. 2m). These results suggest that iron can directly elicit MSRA upregulation and regulate the properties of white adipocytes.

In mammalian cells, iron homeostasis is partly maintained by the iron regulatory proteins IRP1 and IRP2, which coordinate expression of various iron metabolism genes post-transcriptionally by recognizing iron-responsive elements (IREs) within the 5′ and 3′ untranslated regions (UTRs) of their mRNA transcripts. Although iron-dependent induction of MSRA also occurs post-transcriptionally (Extended Data Fig. 2g–i), an in silico search^36^ failed to identify candidate IREs in the UTR sequences of mouse or human MSRA. Therefore, we asked whether iron could upregulate post-translational MSRA expression by enhancing its protein stability. Indeed, pulse–chase experiments revealed that the half-life of MSRA is markedly prolonged when adipocyte cultures (Fig. 2b) and 293T cells (Extended Data Fig. 2n) were supplemented with iron in the form of ferric ammonium citrate (FAC). Iron-induced stabilization of MSRA is independent of ferroptotic responses, as it persists even when cells are cotreated with the ferroptosis inhibitor liproxstatin 1 (Extended Data Fig. 2o).

## Iron promotes the formation of an active MSRA oligomer

To determine whether the stabilization of MSRA involves a direct physical interaction with iron, we first analyzed its metal-binding properties. Of note, the absorbance spectrum of purified recombinant mouse (Fig. 2c) and human (Extended Data Fig. 2p) MSRA was markedly shifted by exposure to iron but not to other metals, including Zn^2+^, Co^2+^, Mn^2+^ and Cu^2+^ (Extended Data Fig. 2q–t). Of note, the absorbance of iron-bound MSRA on the ultraviolet (UV)–visible spectrum is consistent with a μ-oxo-bridged diiron (Fe–O–Fe)^37^ (Fig. 2c). Furthermore, using ICP–MS, we confirmed that iron content was substantially enriched in MSRA immunoprecipitants relative to other metals of equal or lower cellular abundance (Fig. 2d) and this enrichment was further increased in cells supplemented with FAC (Extended Data Fig. 2u). Together, these data suggested that MSRA is a metalloprotein with selective iron-binding capacity.

**Extended Data Fig. 3:**
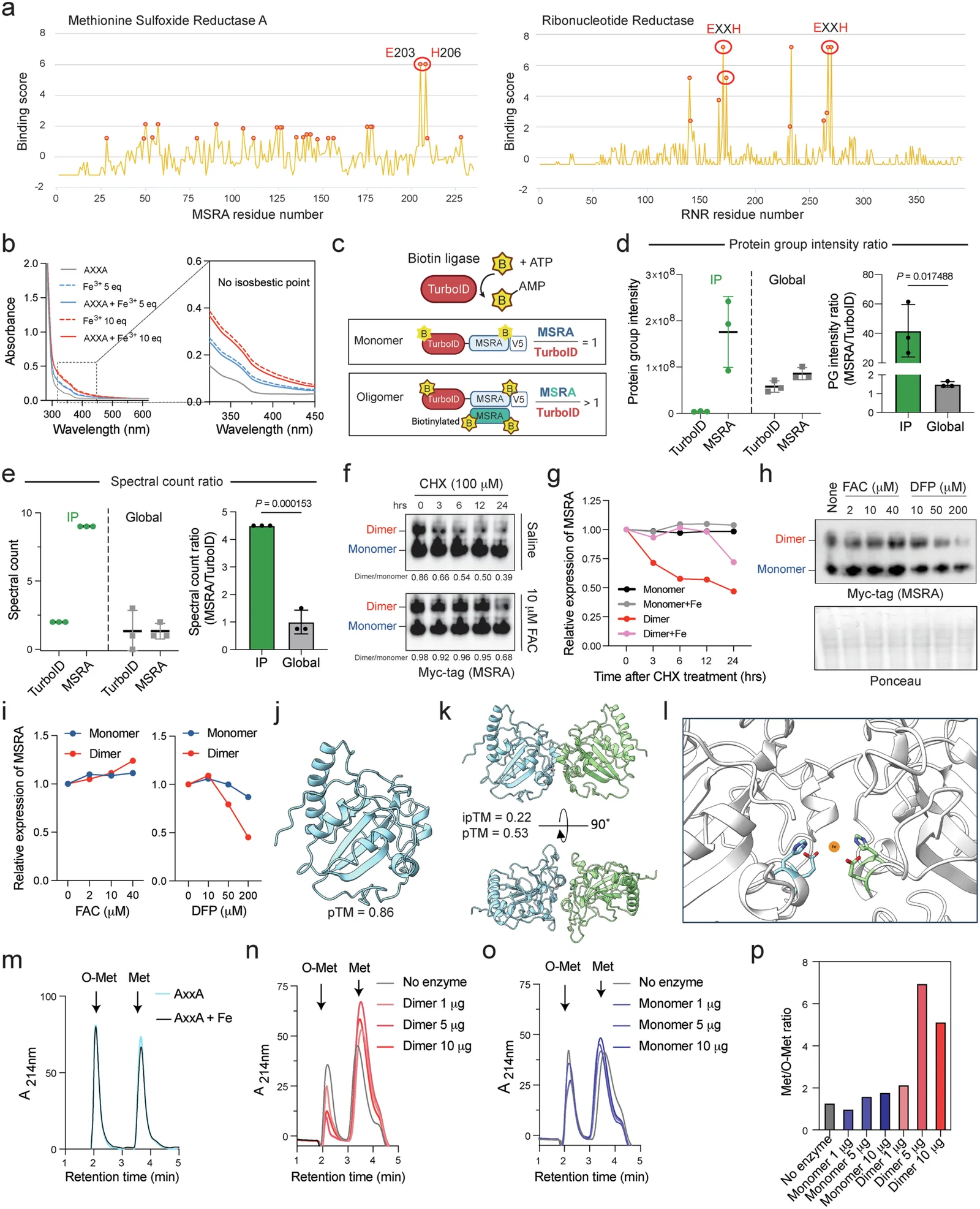
Iron coordinates MSRA dimerization through its conserved EXXH motif. **a**, Metal Ion-Binding site prediction of MSRA (left) and Ribonucleotide Reductase (right). **b**, Absorbance properties of recombinant human MSRA iron domain mutant (AXXA) in the presence of Fe. Inset, zoomed-in spectra of MSRA in the presence of ferric ammonium citrate (FAC). 5 eq, five molar equivalents in concentration. Isosbestic point: A point observed in overlaid spectra when a chromophoric precursor is converted to a product with a different spectrum. An isosbestic point occurs only when the precursor is quantitatively converted to a single product. **c**, TurboID experimental design to discern whether MSRA exists as a monomer or oligomer in iron-replete cells using mass spectrometry. Blue, MSRA tagged to the biotin ligase TurboID; Teal, endogenous MSRA. B = Biotin. **d**,**e**, Mass spectrometric abundance (**d**) and spectral counts (**e**) of TurboID and MSRA peptides in whole cell lysates (Global) and biotin-enriched (IP) proteome. Each dot represents an independent cell culture replicate (*n* = 3). Error bars in this Fig. are means ± SDs. A two-tailed unpaired *t*-test was performed. **f**-**i**, Native PAGE analysis and quantification of MSRA expression in white adipocyte cultures in the presence of exogenous ferric ammonium citrate (FAC) (**f**,**g**) or iron chelator deferiprone (DFP) (**h**,**i**). **j**,**k**, AlphaFold structure prediction of MSRA monomer (**j**) and dimer (**k**) in the absence of metal. Predicted structure of MSRA monomer, pTM = 0.86 (left). **l**, Predicted MSRA dimer interface. **m**-**p**, Activity assay of MSRA iron domain mutant (E203A-H206A, AXXA) (**m**), or increasing concentrations of gel filtration purified his-tagged mouse MSRA dimer (**n**) and monomer (**o**), in the presence of 1:1 molar ratio of reduced (Met) and oxidized (O-Met) methionine after reconstitution with 5 molar equivalents of ferric ammonium citrate (FAC). Reductase activity is measured based on the quantification of Met/Met-O ratio (**p**). Data are representative of three independent experiments with similar results. Source data

To ascertain how MSRA interacts with iron, we examined its amino acid sequence using the metal-ion-binding site prediction algorithm MIB2 (ref. ^38^). This analysis suggested that residues E203 and H206, which are highly conserved in MSRA orthologs across the evolutionary spectrum (Fig. 2e), have iron-binding potential (Extended Data Fig. 3a). Indeed, the absorbance spectrum of MSRA-AXXA, a mutant lacking both conserved residues (EDYH→ADYA), is insensitive to iron, unlike that of WT MSRA (Extended Data Fig. 3b). Moreover, the protein expression levels of MSRA-AXXA but not MSRA mutants lacking the myristoylation site (G22A) or active-site cysteine (C72S), were significantly suppressed (Fig. 2f). The EXXH motif of MSRA bears some resemblance to the Fe-binding centers of several nonheme proteins that coordinate iron through the carboxylate and imidazole groups of their conserved glutamic acid and histidine residues, respectively^39^. However, unlike MSRA, most members of the ferritin-like protein superfamily, including the β subunit of class I ribonucleotide reductases (RNRs), possess two conserved EXXH motifs that cooperate to form a diiron center containing two oxygen-bridged iron atoms^40,41^. For example, the two EXXH motifs of RNR, albeit widely separated within its primary amino acid sequence (Extended Data Fig. 3a), converge in space to coordinate its diiron center (Fig. 2g)^40,41^. Interestingly, the electrostatic environment surrounding the predicted iron-binding EXXH motif of MSRA bears a striking resemblance to a half-site of the RNR diiron center (Fig. 2g). This observation suggested that MSRA dimerization might allow for the assembly of a full diiron center, which in turn might enhance the stability and even the enzymatic activity of MSRA.

To determine whether MSRA can oligomerize in vivo, we initially used proximity-dependent biotinylation to evaluate interactions between MSRA protomers under normal (that is, iron-replete) cellular conditions. Therefore, an MSRA construct fused to an engineered biotin ligase (TurboID)^42^ was expressed ectopically and the streptavidin-bound fraction of cell lysates was subjected to MS. If MSRA exists primarily as a monomer, then the ratio of peptides derived from MSRA versus TurboID sequences should approximate 1:1 (Extended Data Fig. 3c). Instead, MSRA peptides were robustly enriched in the biotinylated (that is, spatially proximal) proteome relative to the global proteome, indicating that ectopic MSRA–TurboID polypeptides readily interact with endogenous MSRA in vivo (Extended Data Fig. 3d,e). Moreover, size-exclusion chromatography coupled to multiangle light scattering (SEC–MALS) showed that iron supplementation directly promotes in vitro formation of a higher-molecular-weight species consistent with the size of an MSRA dimer (Fig. 2h). Indeed, native PAGE analysis of cell lysates revealed two distinct in vivo MSRA species: a lower-molecular-weight form (monomer) and a higher-molecular-weight form (dimer). Of these, the latter is more labile but is stabilized by exogenous iron supplementation (Fig. 2i,j and Extended Data Fig. 3f,g) and persists following liproxstatin 1 cotreatment (Fig. 2k). Increasing iron concentration promoted dimer formation, whereas iron chelation with DFP decreased the abundance of both forms, with a more pronounced decrease in the dimer (Extended Data Fig. 3h,i). Importantly, both in vivo dimer formation in adipocytes (Fig. 2l) and iron-induced dimerization of recombinant MSRA in vitro (Fig. 2m) were abolished by individual (E203A, H206A) and combined (AXXA) substitutions in the EXXH motif.

To investigate how MSRA dimerizes at the molecular level, we first performed in silico modeling of the mouse apo MSRA crystal structure using AlphaFold3 (ref. ^43^) and HADDOCK^44^. Both algorithms generated a structurally similar dimeric interaction between two MSRA protomers (Extended Data Fig. 3j,k), with low-confidence scores that likely reflect the absence of iron. Consistent with our experimental evidence that iron directly promotes MSRA dimerization (Fig. 2h), the EXXH motifs from each protomer are spatially juxtaposed in the predicted dimer configuration in a manner that would coordinately bridge iron and potentially stabilize the MSRA dimer (Extended Data Fig. 3l). Strikingly, PropKa analysis^45^ predicted that iron-mediated dimerization would also decrease the pKa of the catalytic residue C72 relative to an apo form MSRA monomer (Fig. 2n), thereby favoring its deprotonated (thiolate) state. As such, dimerization through EXXH–mediated iron coordination should enhance the nucleophilicity and catalytic efficiency of the active-site cysteine (Fig. 2n). Consistent with this prediction, iron supplementation increased the activity of recombinant WT MSRA in a dose-dependent manner (Fig. 2o). In contrast, the MSRA-E203A mutant showed a diminished response to iron (Fig. 2p), while MSRA-AXXA was entirely unresponsive (Extended Data Fig. 3m). Furthermore, dimeric MSRA exhibited significantly higher reductase activity than the monomeric form (Fig. 2q and Extended Data Fig. 3n–p). Together, these findings indicate that iron enhances the enzymatic activity of MSRA by promoting dimerization through its juxtaposed EXXH motifs.

## Chemoproteomic analysis identifies MSRA substrates during cancer-induced browning

**Fig. 3:**
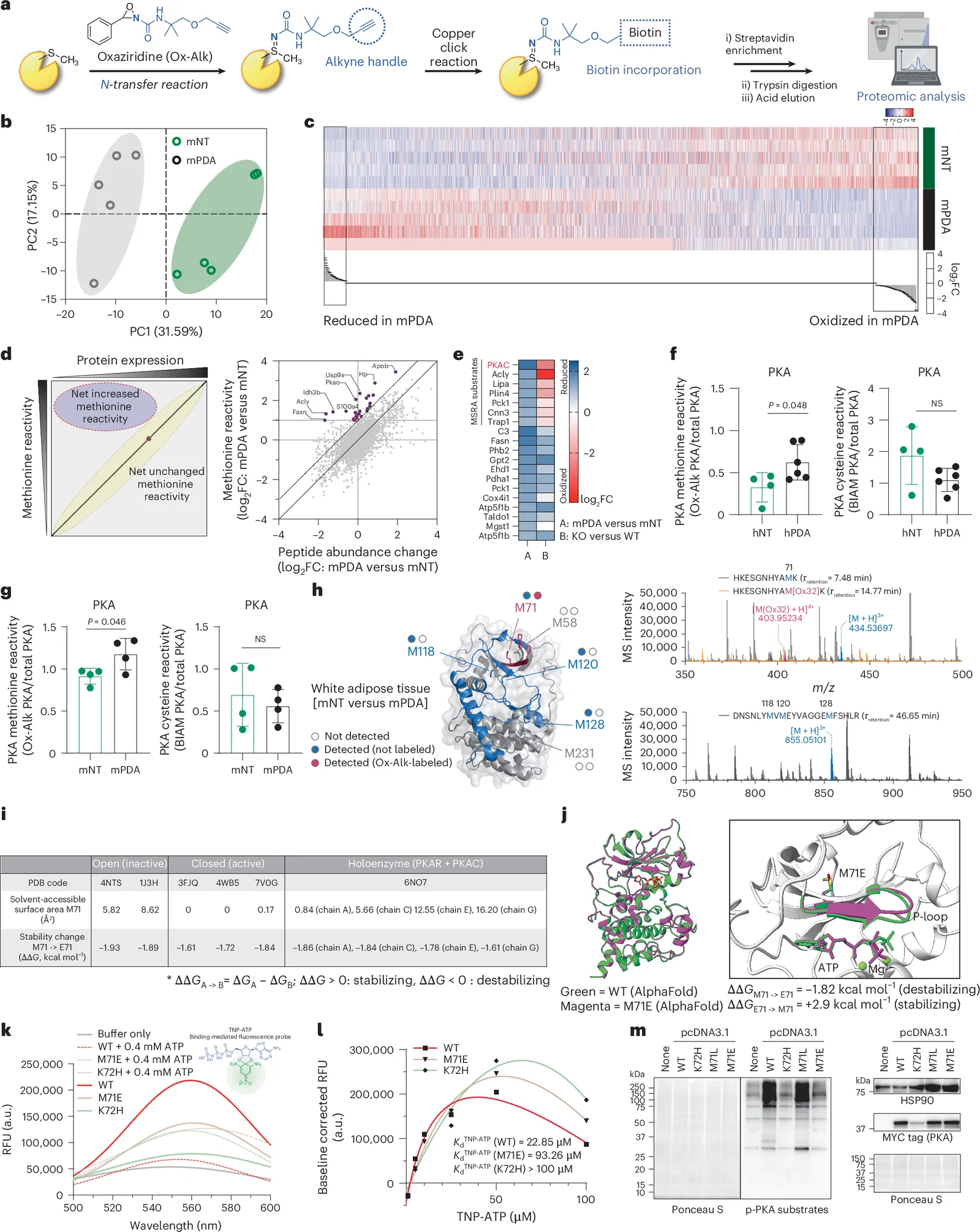
Chemoproteomic analysis identifies PKA as a key substrate of MSRA for cancer-induced browning. **a**, Chemoproteomic analysis workflow. **b**, Principal component analysis of the reactive methionine proteome of sWAT tissues from mPDA mice (*n* = 5) compared to age-matched (6–8 months old) mNT controls (*n* = 5). **c**, Heat map of activity-based, oxidation-sensitive methionine sWAT proteomes. **d**, Methionine reactivity of each peptide is plotted as a function of its abundance. Peptides in the top left quadrant denote increased reactivity independent of protein expression. The peptides detected exclusively in the chemoproteomics analysis were excluded from this analysis. Net methionine reactivity was defined as the difference between log_2_FC_IP and log_2_FC_global (ΔFC). Peptides with ΔFC > 1 were defined as exhibiting increased net methionine reactivity (blue dot with red line). **e**, Heat map of proteins that are significantly reduced in mPDA compared to mNT and their redox status in *MsrA*-KO mice compared to WT (mNT). If multiple peptides from the same protein exhibit increased net methionine reactivity, the peptide with the highest intensity is shown in the heat map. The scale bar denotes log_2_FC. **f**,**g**, PKA methionine oxidation status by Ox-Alk and cysteine oxidation status by BIAM analysis in human (**f**) and mouse (**g**) sWAT. hNT, *n* = 4 participants; hPDA, *n* = 6 participants; mNT, *n* = 4 mice; mPDA, *n* = 4 mice. **h**, Left: methionine residues in PKAC are annotated on its protein structure. MS-detected methionine peptides are marked in blue, while Ox-Alk (Ox32)-reactive ones are marked in red. Right: spectral signal. **i**, A table showing the analysis of SASA (Å^2^) for residue M71 of PKAC and the analysis of protein stability change (kcal mol^−1^) with M71E substitution in PKAC. **j**, AlphaFold-predicted structures of PKAC WT and M71E mutant. Protein stability changes upon M71E substitution in PKAC WT and upon E71M substitution in PKAC-M71E. **k**,**l**, TNP-ATP binding assay (**k**) and saturation curve (**l**) to compare affinities of ATP to the catalytic subunit of PKA. ATP was added to outcompete TNP-ATP to ascertain signal specificity. **m**, PKA substrate phosphorylation in 293T cells upon ectopic expression of the WT or mutant (K72H, M71L or M71E) forms of PKA. Error bars represent the mean ± s.d. A two-tailed unpaired Student’s *t*-test was performed. Source data

To identify methionine residues that serve as MSRA enzymatic substrates in adipose tissue, we surveyed the WAT proteome using Ox-Alk bioconjugation followed by MS to distinguish between reduced and oxidized methionines^29,46^ (Fig. 3a). From this analysis, we identified and quantified 4,655 peptides containing reactive (that is, reduced) methionine, derived from 1,204 distinct proteins across all samples (Supplementary Table 3). Principal component analysis revealed a clear separation between the reactive methionine proteomes of tumor-bearing (mPDA) and control (mNT) mice (Fig. 3b), with mPDA samples showing a net increase in methionine oxidation—88 peptides significantly more oxidized (log_2_FC (fold change) < −1, *P* < 0.05) versus 47 that were reduced (log_2_FC > 1, *P* < 0.05) (Fig. 3c), consistent with our global oxidation analysis (Fig. 1j). As MSRA is upregulated in PDA WAT, we reasoned that its direct substrates would show decreased methionine oxidation in the tumor setting and increased oxidation in *MsrA*-null (knockout, KO) WAT. Applying these criteria and adjusting for expression differences (Fig. 3d), we identified 19 candidates with significantly reduced methionine oxidation in mPDA samples (log_2_FC > 1, *P* < 0.05), seven of which are more oxidized in *MsrA*-null WAT (Fig. 3e). These findings suggest that MSRA can coordinate the pleiotropic program of adipose browning by regulating methionine redox states of multiple select protein substrates.

**Extended Data Fig. 4:**
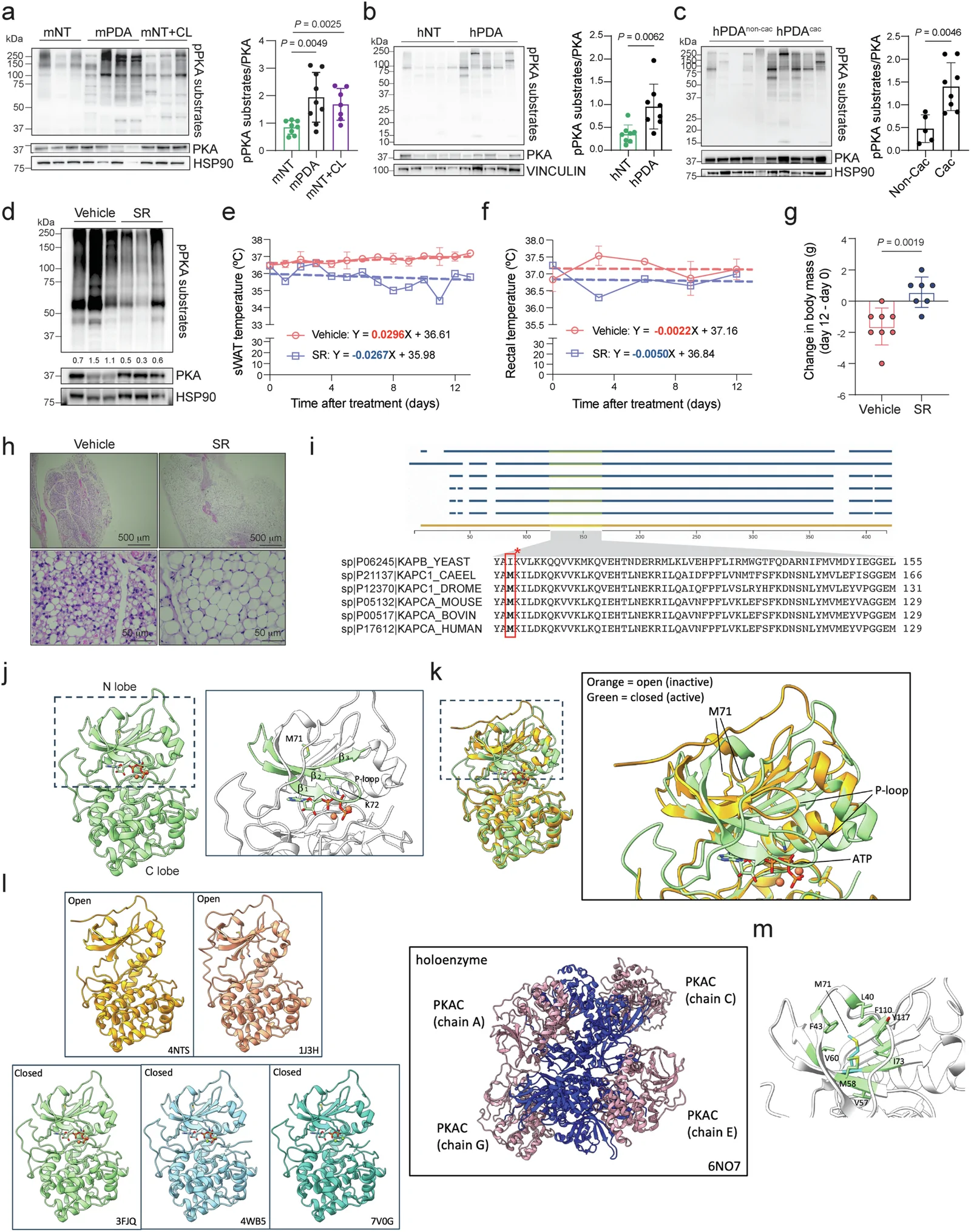
PKAC methionine redox state and β3AR-PKA signalling are altered during cachectic browning. **a**-**c**, Global phosphorylation of PKA substrates in murine (**a**) and human (**b**,**c**) WAT specimens was measured by immunoblot analysis and quantified relative to total PKA expression. Each lane represents lysate from an independent mouse or patient. mNT (*n* = 8 mice), non-tumour bearing wildtype control; mPDA (*n* = 9 mice), KPC mice with pancreatic tumour; mNT+CL (*n* = 7 mice), non-tumour bearing wildtype treated with 5 mg/kg β3-adrenergic agonist CL-316243 (CL) for 6 days; hPDA^CAC^ (*n* = 8 patients), cachectic PDA patients (body mass loss >5%); hPDA^nonCAC^ (*n* = 5 patients), non-cachectic PDA patients (body mass loss <5%); hNT (*n* = 8 patients), non-tumour patients who underwent laparoscopic cholecystectomy (hNT). Error bars in this Fig. are means ± SDs. A two-tailed unpaired *t*-test was performed. **d**, Immunoblot analysis of sWAT specimens to measure global phosphorylation of PKA substrates after 12 days of daily intraperitoneal administration of 200 μL vehicle (10% DMSO, 40% PEG300, 5% Tween80, 80% saline) or 5 mg/kg β3-adrenergic antagonist SR59230a. Each lane represents lysate from an independent mouse. Vehicle, *n* = 3 mice; SR, *n* = 3 mice. **e**,**f**, Measurements of local sWAT heat generation by telemetric implants (**e**) and core body temperature by rectal probe (**f**) over the course of a 12-day treatment with vehicle or β3-adrenergic antagonist SR59230a. The rate of change is indicated in bold for each curve. Vehicle, *n* = 3 mice; SR, *n* = 2 mice. **g**,**h**, Body mass (**g**) and subcutaneous white adipose tissue (sWAT) histology (**h**) of KPC mice over the course of a 12-day treatment with vehicle or β3-adrenergic antagonist SR59230a. Vehicle, *n* = 8 mice; SR, *n* = 7 mice. **i**, Sequence alignment of P-loop sequences from the PKA catalytic subunit showing phylogenetic conservation of the redox-regulated M71 methionine (red box) and ATP-binding K72 lysine (asterisk). **j**, Crystal structure (PDB: 3FJQ) of PKA catalytic subunit (PKAC). P-loop and residues M71, K72, ATP (stick structure), and Mn^2+^ (orange spheres) are highlighted. **k**, A comparison of open/inactive (orange, PDB: 4NTS) and closed/active (green, PDB: 3FJQ) conformations of PKAC. Relative locations of residue M71 and P-loop are shown for each state. **l**, Published crystal structures of PKAC in open and closed conformations and holoenzyme (PKA regulatory subunit in blue, catalytic subunit in pink). PDB accession codes are shown in the panels. **m**, Crystal structure of PKAC-WT (PDB: 3FJQ) with highlighting the M71 methionine and surrounding hydrophobic residues. Source data

Among these candidate substrates, the catalytic subunit of protein kinase A (PKAC) stood out for its established role in β3AR-induced thermogenesis^47^. To validate our proteomic screen, the PKAC oxidation status of independent WAT samples was compared between PDA-bearing versus nontumor individuals. In both human (Fig. 3f) and mouse (Fig. 3g) WAT specimens, we observed a consistent decrease in PKAC methionine oxidation in PDA-bearing individuals, while PKAC cysteine oxidation remained unchanged (Fig. 3f,g). In line with the elevated sympathetic tone of WAT in PDA-bearing mice (Fig. 1b,c), we also observed altered phosphorylation patterns of global PKA substrates in cachectic WAT from both tumor-bearing mice (Extended Data Fig. 4a) and humans (Extended Data Fig. 4b,c). Moreover, pharmacological inhibition of β3AR signaling in PDA mice suppressed PKA substrate phosphorylation (Extended Data Fig. 4d), abrogated sWAT-mediated heat dissipation (Extended Data Fig. 4e) without altering core body temperature (Extended Data Fig. 4f) and attenuated PDA-associated weight loss (Extended Data Fig. 4g) and the histological features of browning (Extended Data Fig. 4h). Together, these data identify PKAC as a candidate effector protein of MSRA and support a role for β3AR–PKA signaling in cancer-induced cachexia.

The sole redox-sensitive methionine residue of PKAC (M71) (Fig. 3h), which is highly conserved across evolution (Extended Data Fig. 4i), lies immediately adjacent to the ATP-binding lysine K72 on the β3 strand of the N-terminal lobe and within the PKAC catalytic cleft that coordinates ATP, Mg^2+^ and substrate binding (Extended Data Fig. 4j)^48^. In its inactive (open) conformation—before ligand binding—the catalytic cleft of PKAC is accessible to both solvent and ligand but it adopts a closed (active) conformation upon ligand engagement (Extended Data Fig. 4k). Thus, we hypothesize that oxidation and reduction of M71 likely occur in the open state, before both substrate and ATP binding. Supporting this, solvent-accessible surface area (SASA) analysis^49^ of published crystal structures revealed that M71 is solvent exposed and readily oxidizable in the open-state apo form of PKAC, as well as in its holoenzyme complex with the PKA regulatory subunit (Extended Data Fig. 4l). By contrast, in the closed conformation, M71 is completely buried within a hydrophobic pocket (Extended Data Fig. 4m) and no longer solvent accessible (Fig. 3i and Extended Data Fig. 4l,m), suggesting that M71 must be in a reduced state for PKAC to adopt its active conformation. Consistent with this model, DynaMut2 (ref. ^50^) analysis of protein stability following the M71E substitution—a structural and electrostatic mimic of oxidized methionine—showed decreased protein stability across all available structures, supporting the idea that M71 oxidation destabilizes PKAC (Fig. 3i). Moreover, AlphaFold-based modeling predicts weaker interactions between the P-loop and ATP in mutant PKAC-M71E relative to WT PKAC; in particular, the M71E substitution in the WT structure decreased protein stability by −1.82 kcal mol^−1^, while reversing the substitution (E71M) increased stability by +2.9 kcal mol^−1^ (Fig. 3j). To validate these in silico data, we next probed the ATP–protein interaction in vitro using a fluorescent ATP analog (TNP-ATP). While a kinase-dead PKAC mutant (K72H) known to lack ATP-binding capacity^51^ displayed no detectable TNP-ATP binding, the PKAC-M71E mutant retained only ~50% of the ATP-binding capacity of WT PKAC (Fig. 3k) and required a fourfold higher ATP concentration to achieve the same level of binding as its WT counterpart (Fig. 3l). Furthermore, cellular expression of either PKAC WT or its redox-insensitive M71L mutant promoted global PKA substrate phosphorylation, while the M71E and K72H mutants failed to do so (Fig. 3m). Collectively, these findings suggest that M71 can serve as a redox-sensitive site to modulate PKAC activity and its reduction by MSRA is essential to maintain full PKA activity in β3AR-stimulated adipocytes.

## MSRA is required for β3AR-induced signaling in adipocytes

**Extended Data Fig. 5:**
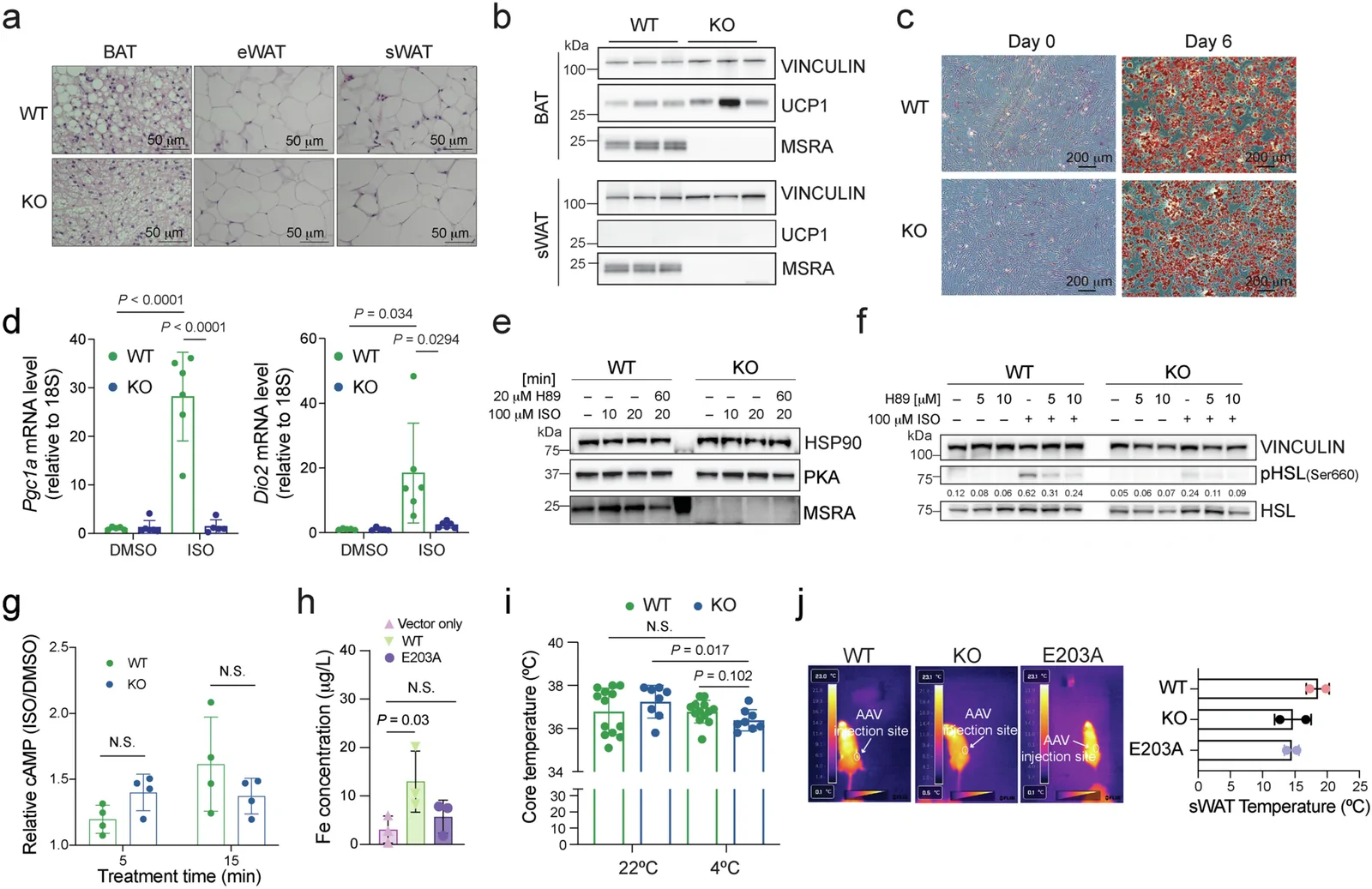
MSRA supports adipocyte β-adrenergic signalling and thermogenesis. **a**, Representative hematoxylin and eosin (H&E) staining of brown (BAT) and white (WAT) adipose tissues from non-tumour-bearing mice. Representative images are shown from three independent mice with similar results (*n* = 3 mice). **b**, Immunoblot analysis of protein expression from BAT and WAT of non-tumour-bearing mice. Each lane represents lysate from an independent mouse. WT, *n* = 3 mice; KO, *n* = 3 mice. **c**, Brightfield images of preadipocyte cultures stained with Oil Red O for lipid droplets after a 6-day differentiation protocol into white adipocytes. Representative images are shown from three independent cell culture replicates with similar results (*n* = 3). **d**, qRT-PCR analysis of brown adipocyte cultures upon treatment with vehicle (DMSO) or 100 μM pan-adrenergic receptor agonist, isoproterenol (ISO) for 24 hours. Each dot represents RNA collected from a separate adipocyte culture at a distinct passage. Each primary adipocyte culture was established from one neonate. WT DMSO, *n* = 5 culture samples from 2 neonates; KO DMSO, *n* = 5 culture samples from 2 neonates; WT ISO, *n* = 6 culture samples from 2 neonates; KO ISO, *n* = 6 culture samples from 2 neonates. **e**, Immunoblot analysis of PKA protein expression in white adipocyte cultures upon treatment with the pan-β-adrenergic receptor agonist (isoproterenol; ISO) for 5 min or the PKA inhibitor (H89) for 60 min at the indicated concentrations. Data are representative of three independent cell culture replicates with similar results. **f**, Immunoblot analysis of activating phosphorylation of hormone lipase (pHSL) in white adipocyte cultures upon treatment with the pan-β-adrenergic receptor agonist (isoproterenol; ISO) or the PKA inhibitor (H89) at the indicated concentrations and times. Data are representative of three independent cell culture replicates with similar results. **g**, Cyclic AMP (cAMP) measurements of white adipocyte cultures upon treatment with the pan-β-adrenergic receptor agonist for the indicated durations. Each dot represents an independent cell culture replicate (*n* = 4). **h**, Inductively coupled plasma mass spectrometric analysis of total iron content in the immunoprecipitants of wildtype (WT) and mutant (E203A) MSRA from white adipocyte cultures. Each dot represents an independent cell culture replicate (*n* = 3). **i**, Core body temperature measurements of mice housed at the indicated temperatures using a rectal probe. WT, *n* = 13 mice; KO, *n* = 8 mice. **j**, Representative thermal images and quantification of local inguinal fat temperature (white circle demarcates the location of AAV injection), calculated as the average of the highest 50% of the region of interest, in adult mice before and after 24 hours of acute cold exposure. WT denotes *MsrA*^−*/*−^ mice receiving AAV expressing *MsrA-WT* cDNA in the inguinal fat pad. E203A denotes *MsrA*^−*/*−^ mice receiving AAV expressing *MsrA-E203A* cDNA in the inguinal fat pad. KO denotes *MsrA*^−*/*−^ mice receiving AAV expressing *mCherry* cDNA in the inguinal fat pad (*n* = 2 mice each). WT, *MsrA*^*+/+*^; KO, *MsrA*^−*/*−^. Error bars in this Fig. are means ± SDs. A two-tailed unpaired Student’s *t*-test was performed. N.S., not significant. Source data

To assess the functional relationship between MSRA and PKA in adipocytes, we first examined adipose tissues from *MsrA*^−/−^ mice, which mature to adulthood with no apparent phenotype in the absence of oxidative stress^52^. The BAT and WAT of *MsrA*-null animals were histologically indistinguishable from those of WT mice (Extended Data Fig. 5a) and BAT-specific expression of UCP1 was unaffected by *MsrA* nullizygosity (Extended Data Fig. 5b). These results indicate that *MsrA* is dispensable for adipose tissue development in vivo. Likewise, ex vivo differentiation of cultured preadipocytes from neonatal adipose tissues^53^ did not require *MsrA* (Extended Data Fig. 5c).

**Fig. 4:**
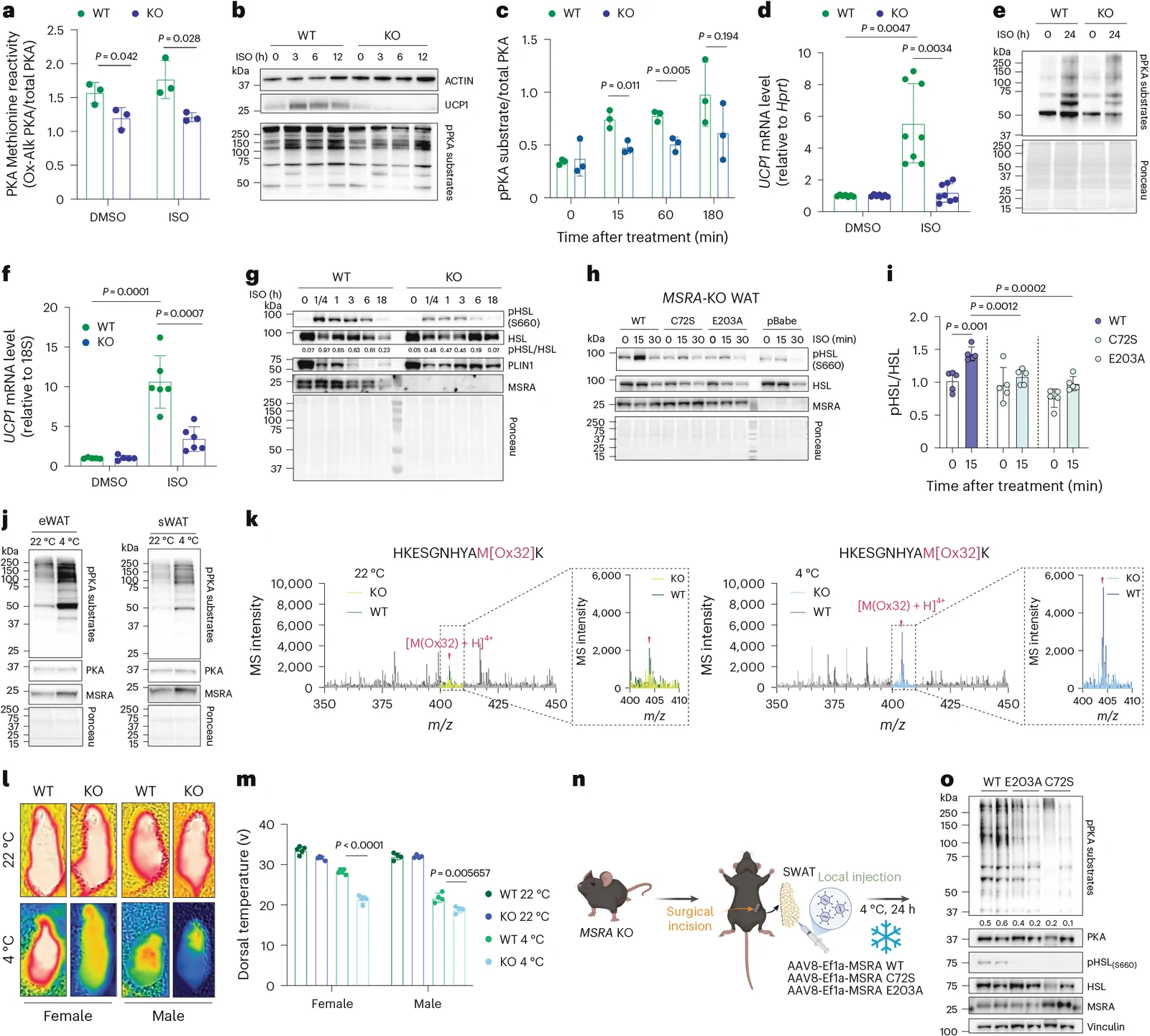
MSRA promotes PKA activation and adipose browning. **a**, PKA methionine oxidation status by Ox-Alk analysis in primary white adipocyte cultures upon adding 100 μM pan-βAR agonist, isoproterenol (ISO). Each dot represents an independent cell culture replicate of primary adipocytes derived from neonates of each genotype (*n* = 3). **b**,**c**, Immunoblot analyses of WAT adipocyte cultures upon stimulation with 100 μM ISO over the indicated duration (**b**). The level of PKA substrate phosphorylation is quantified relative to the total PKA expression at each time point (**c**). Each dot represents an independent cell culture replicate of primary adipocytes derived from neonates of each genotype (*n* = 3). **d**, RT–qPCR analysis of primary white adipocyte cultures treated with vehicle (DMSO) or 100 μM ISO for 24 h. Each dot represents RNA collected from a separate adipocyte culture at a distinct passage. Each primary adipocyte culture was established from one neonate. WT DMSO, *n* = 7 culture samples from three neonates; KO DMSO, *n* = 7 culture samples from three neonates; WT ISO, *n* = 8 culture samples from three neonates; KO ISO, *n* = 8 culture samples from three neonates. **e**, Immunoblot analyses of BAT adipocyte cultures upon stimulation with 100 μM ISO over the indicated duration. Data are representative of two independent experiments with similar results. **f**, RT–qPCR analysis of brown adipocyte cultures upon treatment with vehicle or 100 μM ISO for 24 h. Each dot represents RNA collected from a separate adipocyte culture at a distinct passage. Each primary adipocyte culture was established from one neonate. WT DMSO, *n* = 5 culture samples from two neonates; KO DMSO, *n* = 5 culture samples from two neonates; WT ISO, *n* = 6 culture samples from two neonates; KO ISO, *n* = 6 culture samples from two neonates. **g**, Immunoblot analyses of white adipocyte cultures upon stimulation with 100 μM ISO over 18 h. Data are representative of three independent experiments with similar results. **h**,**i**, Immunoblot analyses (**h**) and quantification (**i**) of HSL phosphorylation in *MsrA*-null white adipocyte cultures reconstituted with WT or mutant forms (C72S or E203A) of MSRA or empty vector (pBABE), followed by stimulation with 100 μM ISO over 30 min. Each dot represents an independent cell culture replicate of primary adipocytes derived from neonates of the indicated genotype (*n* = 5). **j**, Immunoblot analysis of WAT tissues of male mice housed at ambient temperature (22 °C) or 4 °C. Data are representative of three independent experiments with similar results. **k**, Mass spectra of the Ox-Alk (Ox32) oxaziridine labeling on M71 residue of PKA from sWAT tissues of *MsrA* WT or KO mice housed at ambient temperature (22 °C) or 4 °C. Spectra shown are aligned to the same retention time and the signal corresponding to the target mass is magnified for clarity. **l**,**m**, Representative thermal images (**l**) and quantification (**m**) of surface body temperature, calculated as the average of the highest 50% of the region of interest, of adult mice before and after cold exposure. WT, *n* = 5 female mice and *n* = 4 male mice; KO, *n* = 5 female mice and *n* = 4 male mice. **n**, Experimental schematic of *MsrA*-null animals receiving MSRA-expressing AAV in their inguinal fat pad. **o**, Immunoblot analysis of total PKA substrates and HSL phosphorylation in the inguinal fat pad of *MsrA*-null animals administered MSRA-expressing AAV, followed by acute cold exposure. Each lane represents lysate from an independent mouse. WT, *n* = 2 mice; E203A, *n* = 2 mice; C72S, *n* = 2 mice. Error bars represent the mean ± s.d. A two-tailed unpaired Student’s *t*-test was performed. Panel **n** created in BioRender; Nam, J. https://BioRender.com/9q4th0u (2026). Source data

As expected, PKAC was more oxidized in *MsrA*-null adipocyte cultures (Fig. 4a) and several lines of evidence indicate that its oxidation compromises PKA signaling output. First, while the pan-βAR agonist isoproterenol induced PKA substrate phosphorylation and UCP1 expression in WT WAT (Fig. 4b–d) and BAT (Fig. 4e,f and Extended Data Fig. 5d) cultures, these effects were impaired in *MsrA*-null cells, despite comparable PKA expression (Extended Data Fig. 5e). Second, *MsrA* loss dampened β3AR–PKA-mediated phosphorylation of HSL (Fig. 4g and Extended Data Fig. 5f) similar to pharmacological PKA inhibition (Extended Data Fig. 5f). Of note, MSRA loss did not impair βAR induction of cAMP levels, indicating that MSRA functions downstream of cAMP production, at the level of PKA catalytic activity, within this adipose signaling pathway (Extended Data Fig. 5g). Third, rescue experiments in *MsrA*^−/−^ adipocytes confirmed that β3AR–PKA signaling is dependent on MSRA, as well as its iron-binding capacity (Extended Data Fig. 5h) and catalytic activity (Fig. 4h,i).

Further supporting its physiological role in adipocyte β3AR–PKA signaling, MSRA expression and PKA substrate phosphorylation were both induced in WAT by acute cold exposure (Fig. 4j). PKAC M71 was also more oxidized in the adipose tissue of *MsrA*-null mice compared to WT mice maintained at ambient temperature (22 °C) and this effect was further augmented upon cold exposure (4 °C) (Fig. 4k). Accordingly, the ability of *MsrA*^−/−^ mice to maintain dorsal and core body temperature in response to cold was significantly impaired (Fig. 4l,m and Extended Data Fig. 5i). Indeed, local restoration of MSRA expression in *MsrA*^−/−^ mice using adeno-associated virus (AAV) delivered to the inguinal fat pad restored cold-induced sWAT local heat generation (Extended Data Fig. 5j) and PKA activity (Fig. 4n,o). In contrast, catalytically inactive (C72S) and iron domain (E203A) mutants of MSRA did not. Thus, the enzymatic activity of iron-bound MSRA is required for β3AR-mediated induction of PKA enzymatic activity and the thermogenic program in white adipocytes, establishing a causal relationship linking MSRA activity, PKAC redox state, PKA enzymatic function and thermogenesis.

## MSRA is essential for PDA-associated adipose atrophy and cachexia

**Fig. 5:**
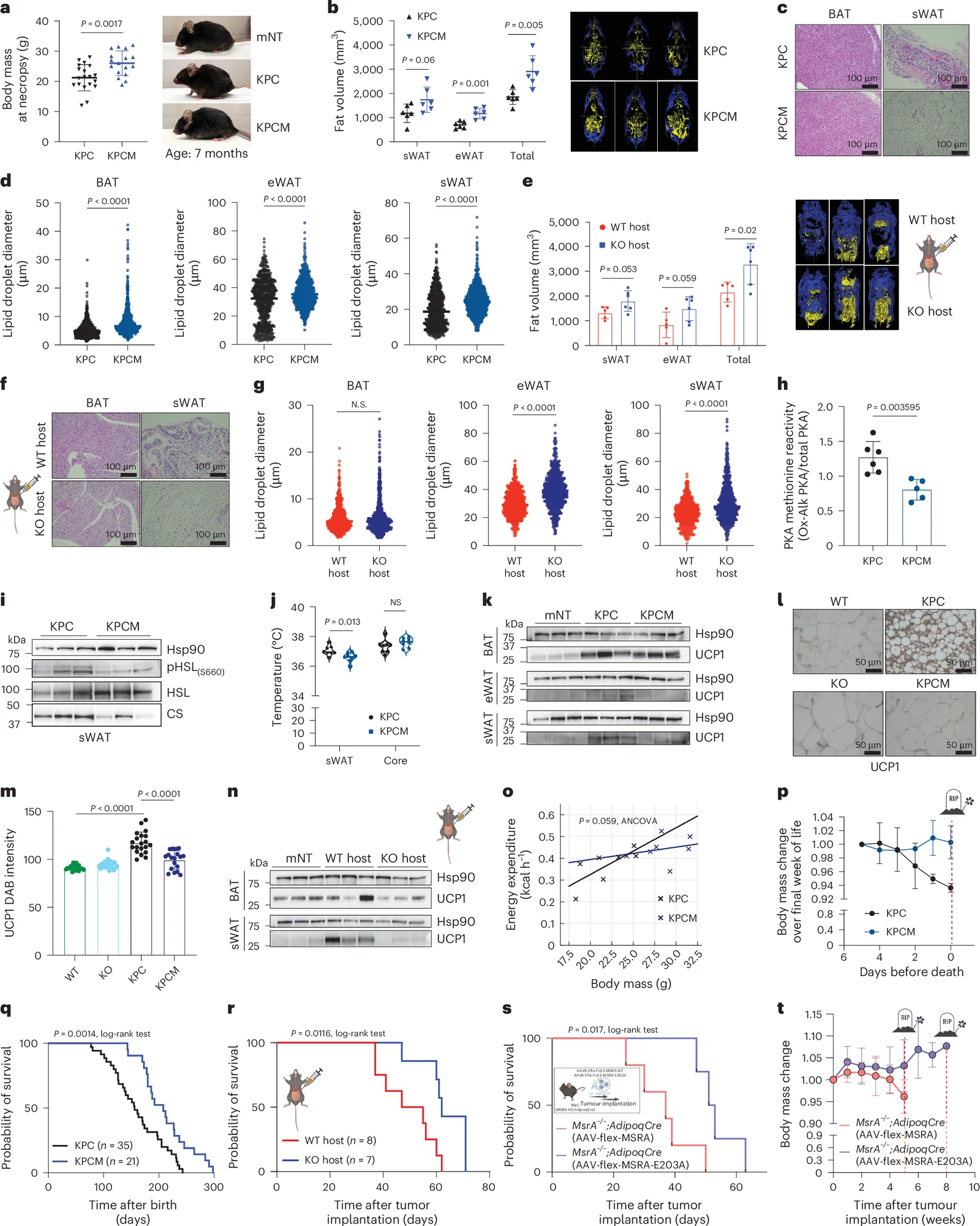
MSRA is required for PDA-associated adipose atrophy and cachexia. **a**, Body mass of pancreatic tumor-bearing mice. KPC, *n* = 19 mice (five males and 14 females); KPCM, *n* = 17 mice (seven males and ten females). **b**, Fat volume analysis by MRI. KPC, *n* = 6 mice (four males and two females); KPCM, *n* = 6 mice (three males and three females). **c**,**d**, Representative H&E staining (**c**) and lipid droplet diameter measurements (**d**) of BAT and WAT. A total of 100 lipid droplets were quantified from each adipose tissue specimen (*n* = 6 mice). **e**, Fat volume analysis by MRI of male mice 5 weeks after orthotopic pancreatic cancer cell injection. WT, *n* = 5 mice; KO, *n* = 6 mice. **f**,**g**, Representative H&E staining (**f**) and lipid droplet diameter measurements (**g**) of BAT and sWAT of mice 5 weeks after orthotopic pancreatic cancer cell injection (*n* = 6 mice). A total of 100 lipid droplets were quantified from each adipose tissue specimen. **h**, PKA methionine oxidation status by Ox-Alk analysis in KPC and KPCM sWAT. KPC, *n* = 6 mice; KPCM, *n* = 5 mice. **i**, Immunoblot analysis of adipose tissues from autochthonous PDA mice. Hsp90 was used as a loading control. Each lane represents lysate from an independent mouse. KPC, *n* = 3 mice; KPCM, *n* = 3 mice. **j**, Inguinal sWAT temperature measured with implanted temperature transponders and core temperature measured with a rectal probe. KPC data points on this graph are the same as mPDA from Fig. 1g. KPC, *n* = 8 mice; KPCM, *n* = 8 mice. **k**, Immunoblot analysis of adipose tissues from autochthonous PDA mice. Hsp90 was used as a loading control. Each lane represents lysate from an independent mouse. mNT, *n* = 3 mice; KPC, *n* = 3 mice; KPCM, *n* = 3 mice. **l**,**m**, Immunohistochemical analysis (**l**) and quantification (**m**) of UCP1 expression in sWAT of tumor-bearing mice compared to age-matched nontumor controls. The values plotted are intensity values of each field of view. WT, *n* = 5 mice; KO, *n* = 5 mice; KPC, *n* = 4 mice; KPCM, *n* = 4 mice. **n**, Immunoblot analysis of adipose tissues from mice 5 weeks after orthotopic pancreatic cancer cell injection. Hsp90 was used as a loading control. Each lane represents lysate from an independent mouse. mNT, *n* = 3 mice, WT, *n* = 3 mice; KO, *n* = 3 mice. **o**, Mean energy expenditure per animal plotted as a function of body mass. An ANCOVA was performed to assess the energy expenditure–body mass relationship of KPC compared to KPCM mice (KPC/mPDA slope = 0.0215 kcal h^−1^ g^−1^; KPCM slope = 0.0057 kcal h^−1^ g^−1^). KPC data points on this graph are the same as mPDA from Fig. 1h. KPC, *n* = 7 mice; KPCM, *n* = 7 mice. **p**, Body mass trajectories of KPC (*n* = 5 mice) and KPCM (*n* = 4 mice) mice during their final week of life. **q**–**s**, Overall survival of autochthonous (**q**), orthotopic transplant (**r**) and orthotopic transplant with adipose-specific MSRA rescue by AAV (**s**) mouse models of PDA. KPC, *n* = 35 mice (15 males and 20 females); KPCM, *n* = 21 mice (nine males and 12 females); WT, *n* = 8 mice; KO, *n* = 7 mice; KO expressing WT MSRA in adipose tissues (*MsrA*^−*/*−^*AdipoqCre*AAV-flex-MSRA), *n* = 5 mice; KO expressing MSRA-E203A in adipose tissues (*MsrA*^−*/*−^*AdipoqCre*AAV-flex-MSRA-E203A), *n* = 4 mice. A log-rank test was performed. **t**, Body mass kinetics of mice after AAV administration and orthotopic transplantation of pancreatic cancer cells over 8 weeks or until the end of life. KO expressing WT MSRA in adipose tissues, *n* = 5 mice; KO expressing E203A MSRA in adipose tissues, *n* = 4 mice. Error bars represent the mean ± s.d. A two-tailed unpaired Student’s *t*-test was performed unless stated otherwise. Panel **s** created in BioRender; Nam, J. https://BioRender.com/iwb178b (2026). Source data

**Extended Data Fig. 6:**
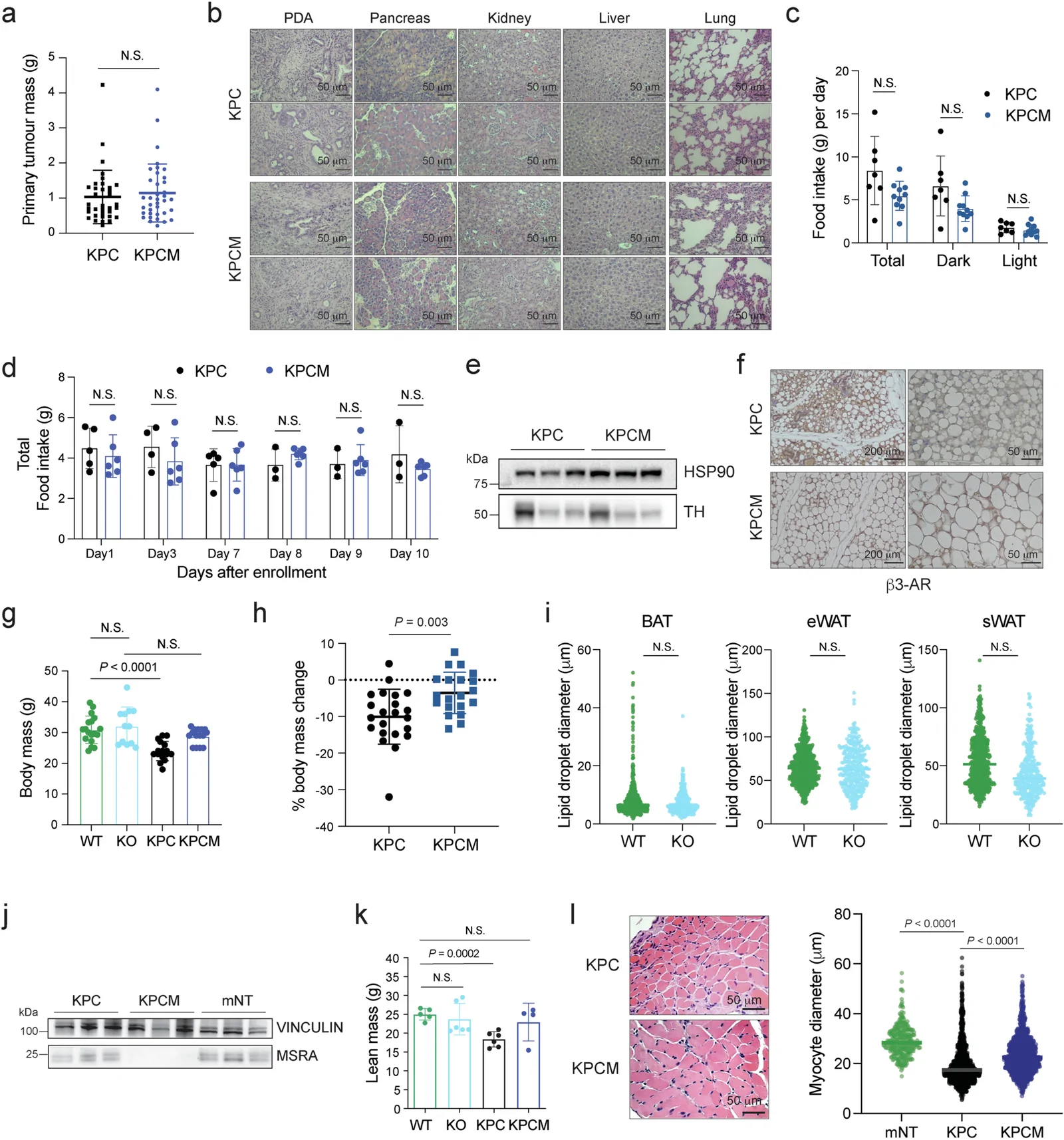
*MsrA* loss suppresses cachectic adipose remodelling in the autochthonous PDA model. **a**, The mass of pancreatic tumours from moribund mice. KPC, *n* = 35 mice; KPCM, *n* = 37 mice. **b**, Representative hematoxylin and eosin (H&E) staining of pancreatic tumour (PDA) and other organs from tumour-bearing mice. Representative images are shown from three independent mice with similar results. **c**, Light and dark cycle food intake. KPC data is identical to mPDA in Extended Data Fig. 1h. KPC, *n* = 7 mice; KPCM, *n* = 10 mice. **d**, Manual measurement of food consumption was conducted by weighing the daily differences in chow pellets for each singly housed mouse during their waking hours (at 6 p.m. and 9 a.m.) over a 10-day period. Actual food intake was calculated by subtracting the mass of ground food on the cage floor. KPC data is identical to mPDA in Fig. 1i. KPC, *n* = 5 mice; KPCM, *n* = 6 mice. **e**, Immunoblot analysis of tyrosine hydroxylase (TH) expression in the sWAT of pancreatic tumour-bearing mice. Each lane represents lysate from an independent mouse. KPC, *n* = 3 mice; KPCM, *n* = 3 mice. **f**, Immunohistochemical analysis of β3-adrenergic receptor (β3-AR) expression in the sWAT of pancreatic tumour-bearing mice. Representative images are shown from three independent mice with similar results. **g**, Body mass of tumour-bearing mice compared to age-matched non-tumour controls. KPC, *Kras*^*G12D*^*;p53*^*R172H*^*;PdxCre;MsrA*^*+/+*^; KPCM, *Kras*^*G12D*^*;p53*^*R172H*^*;PdxCre;MsrA*^−*/*−^*;* WT, non-tumour *MsrA*^*+/+*^; KO, non-tumour *MsrA*^−*/*−^. WT, *n* = 18 mice; KO, *n* = 12 mice; KPC, *n* = 16 mice; KPCM, *n* = 15 mice. **h**, Percentage change of body mass loss over 4 weeks in pancreatic-tumour-bearing mice with late-stage disease. KPC, *n* = 22 mice (13 males and 9 females); KPCM, *n* = 20 mice (9 males and 11 females). **i**, Lipid droplet diameter measurements of adipose tissues in WT and KO non-tumour bearing animals. 100 lipid droplets were quantified from each adipose tissue specimen. BAT: WT, *n* = 5; KO, *n* = 3. eWAT and sWAT: WT, *n* = 6; KO, *n* = 3 mice. **j**, Immunoblot analysis of MSRA expression in tibial skeletal muscles of age-matched tumour-bearing mice compared to age-matched non-tumour controls (mNT). Each lane represents lysate from an independent mouse. KPC, *n* = 3 mice; KPCM, *n* = 3 mice; mNT, *n* = 3 mice. **k**, Lean mass of tumour-bearing mice compared to age-matched non-tumour controls. WT, *n* = 5 mice; KO, *n* = 6 mice; KPC, *n* = 6 mice; KPCM, *n* = 4 mice. **l**, Skeletal muscle cell diameter of tumour-bearing mice compared to age-matched non-tumour controls (mNT). 100 myocytes were quantified from each muscle specimen. mNT, *n* = 3 mice; KPC, *n* = 13 mice; KPCM, *n* = 14 mice. Error bars in this Fig. are means ± SDs unless indicated otherwise. A two-tailed unpaired Student’s *t*-test was performed. N.S., not significant. Source data

To determine whether the thermogenic functions of MSRA are also required for adipose remodeling in response to tumor development, we interbred KPC and *MsrA*^−/−^ mice to generate KPCM compound mutant (*Kras*^G12D^;*p53*^R172H^;*PdxCre*;*MsrA*^−*/*−^) animals. No apparent effects of *MsrA* loss were observed on primary tumor burden (Extended Data Fig. 6a), the macroscopic or histological appearance of other organs (Extended Data Fig. 6b), food intake (Extended Data Fig. 6c,d), sWAT sympathetic tone (Extended Data Fig. 6e) or sWAT β3AR expression (Extended Data Fig. 6f). However, comparing KPC and KPCM mice with similar tumor burdens revealed that body mass attrition was significantly mitigated by the absence of *MsrA*, both during early (tumor diameter: 5–8 mm; Extended Data Fig. 6g) and late (tumor diameter: 9–10 mm; Fig. 5a and Extended Data Fig. 6h) stages of disease. *MsrA* deletion also preserved adipose tissue volume (Fig. 5b) and lipid droplet diameter in mice bearing early-stage tumors (Fig. 5c,d). Meanwhile, loss of MSRA in nontumor mice had no impact on body mass (Extended Data Fig. 6g) or adipocyte diameter (Extended Data Fig. 6i). Interestingly, although MSRA expression was not induced in the skeletal muscles of KPC mice (Extended Data Fig. 6j), the absence of *MsrA* did preserve lean mass (Extended Data Fig. 6k) and skeletal myocyte diameter (Extended Data Fig. 6l) in KPCM mice, consistent with the notion that adipose atrophy precedes and possibly facilitates muscle wasting during cancer cachexia^7,15,16,18^.

**Extended Data Fig. 7:**
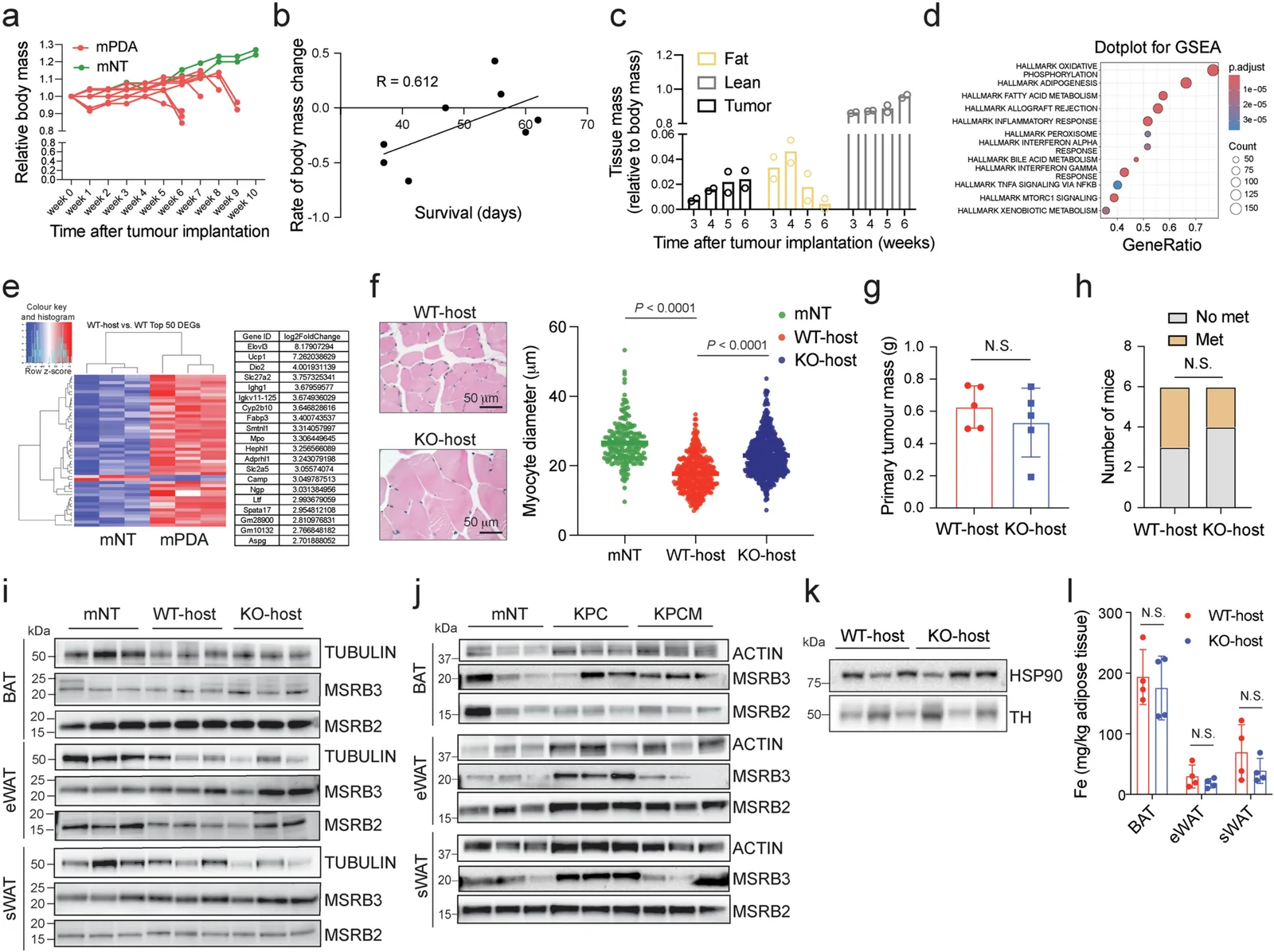
Host *MsrA* loss attenuates cachexia in the orthotopic PDA model without altering tumour progression. **a**, Relative body mass of male mice after orthotopic transplantation of pancreatic cancer cells (mPDA) compared to sex and age-matched non-tumour controls (mNT). Each line represents one mouse. mNT, *n* = 2 mice; mPDA, *n* = 8 mice. **b**, Correlation of survival and the rate of body mass change over the course of tumour development in eight mice orthotopically transplanted with pancreatic cancer cells. **c**, Pancreatic tumour, fat, and lean mass normalized to body mass between 3 and 6 weeks after orthotopic implantation of pancreatic cancer cells (*n* = 2 mice). **d**, Gene Set Enrichment Analysis of the sWAT transcriptome of male mice after orthotopic transplantation of pancreatic cancer cells compared to age-matched non-tumour male controls (*n* = 3 mice per group). **e**, Top 20 upregulated genes in the sWAT of male mice after orthotopic transplantation of pancreatic cancer cells compared to age-matched non-tumour male controls. **f**, Skeletal muscle cell diameter of male mice 5 weeks after orthotopic transplantation of pancreatic cancer cells, compared to age-matched non-tumour male controls (mNT). 100 myocytes were quantified from each muscle specimen. WT-host, *MsrA*^*+/+*^; KO-host, *MsrA*^−*/*−^.mNT, *n* = 2 mice; WT-host, *n* = 4 mice; KO-host, *n* = 6 mice. **g**,**h**, Tumour burden (**g**) and metastatic incidence (**h**) of male mice 5 weeks after orthotopic transplantation of pancreatic cancer cells. WT-host, *n* = 5 mice; KO-host, *n* = 5 mice. **i**, Immunoblot analysis of MSRB2 and MSRB3 expression in the adipose tissues from male mice 5 weeks after orthotopic transplantation of pancreatic cancer cells, compared to age-matched non-tumour controls (mNT). Each lane represents lysate from an independent mouse. mNT, *n* = 3 mice; WT-host, *n* = 3 mice; KO-host, *n* = 3 mice. **j**, Immunoblot analysis of MSRB2 expression in the adipose tissues from PDA-bearing mice compared to non-tumour controls (mNT). KPC, *Kras*^*G12D*^*;p53*^*R172H*^*;PdxCre;MsrA*^*+/+*^; KPCM, *Kras*^*G12D*^*;p53*^*R172H*^*;PdxCre;MsrA*^−*/*−^. Each lane represents lysate from an independent mouse. mNT, *n* = 3 mice; KPC, *n* = 3 mice; KPCM, *n* = 3 mice. **k**, Immunoblot analysis of tyrosine hydroxylase (TH) expression in the sWAT from male mice 5 weeks after orthotopic transplantation of pancreatic cancer cells. Each lane represents lysate from an independent mouse. WT-host, *n* = 3 mice; KO-host, *n* = 3 mice. **l**, Inductively coupled plasma mass spectrometry (ICP-MS) to measure total iron content in adipose tissues from male mice 5 weeks after orthotopic transplantation of pancreatic cancer cells. WT-host, *n* = 4 mice; KO-host, *n* = 4 mice. Error bars in this Fig. are means ± SDs unless indicated otherwise. A two-tailed unpaired Student’s *t*-test was performed. N.S., not significant. Source data

To evaluate whether MSRA has a cancer-cell-extrinsic role in PDA-associated cachexia^29^, we tested whether PDA cells induce adipose and muscle atrophy to differing extents when orthotopically transplanted into wild-type (WT-host) or *MsrA*-null (KO-host) mice. As expected, WT-host tumor recipients progressively lost body mass during tumor progression (Extended Data Fig. 7a), with weight loss correlating inversely with overall survival (Extended Data Fig. 7b). Consistent with prior reports that adipose atrophy is an early event in cancer cachexia^14,54,55^, loss of adipose tissue coincided with tumor growth and preceded detectable changes in lean mass (Extended Data Fig. 7c). At 4–5 weeks after transplantation—when adipose depletion is most robust (Extended Data Fig. 7c)—the sWAT transcriptome (Supplementary Table 4) was enriched for oxidative phosphorylation genes (Extended Data Fig. 7d) and thermogenic markers such as *Elovl3*, *Ucp1* and *Dio2* (Extended Data Fig. 7e). Using this timeline, we then compared cachexia development in age-matched *MsrA*^+/+^ (WT-host) and *MsrA*^−/−^ (KO-host) males following orthotopic PDA cell injection. As in the autochthonous KPC model (Fig. 5a–d), *MsrA* deletion in the host attenuated both adipose (Fig. 5e) and muscle (Extended Data Fig. 7f) wasting 5 weeks after tumor inoculation, without significantly altering primary tumor burden (Extended Data Fig. 7g) or metastatic incidence (Extended Data Fig. 7h). Histologically, *MsrA* loss also reduced the multilocular transformation and wasting of WAT adipocytes in tumor-bearing mice (Fig. 5f,g).

At the adipocyte level, PKAC methionine reactivity (Fig. 5h), markers of lipolysis (Fig. 5i), local sWAT temperature (Fig. 5j) and UCP1 expression (Fig. 5k–n) were all decreased in both autochthonous (KPC) and orthotopic transplant PDA models by *MsrA* loss. Consistent with these thermogenic defects, the hypermetabolic phenotype of KPC mice was markedly attenuated upon *MsrA* deletion (Fig. 5o). These defects were not accompanied by compensatory upregulation of methionine sulfoxide reductase B family members (MSRB2 and MSRB3) (Extended Data Fig. 7i,j) or by changes in sympathetic innervation (Extended Data Fig. 7k) or iron content (Extended Data Fig. 7l), indicating a specific requirement for MSRA.

**Extended Data Fig. 8:**
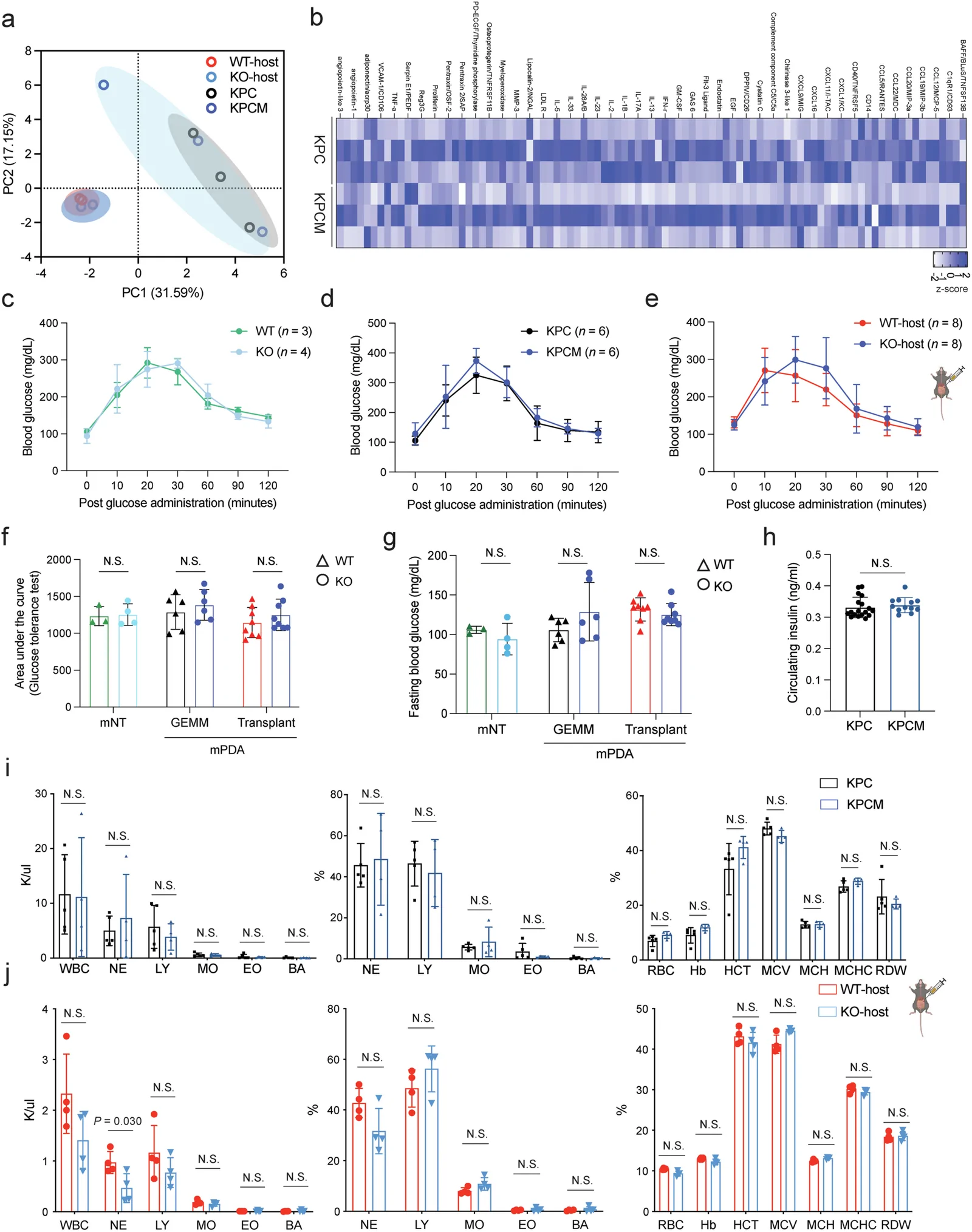
MSRA does not broadly control systemic inflammatory or glucose homeostatic responses in PDA cachexia. **a**,**b**, Principal component analysis (**a**) and heatmap (**b**) of circulating levels of cytokine and adipokines of the serum from KPC vs KPCM mice (*n* = 3 mice each) and WT-host vs KO-host (*n* = 2 mice each). Heatmap: Row, Z-score for the expression of each cytokine. Column, biological replicate. The full list of cytokines and adipokines analysed is provided in Supplementary Table 5. **c**-**e**, Glucose tolerance test in non-tumour WT (*n* = 3 mice) and KO (*n* = 4 mice) (**c**), KPC (*n* = 6 mice) and KPCM (*n* = 6 mice) (**d**), as well as WT (*n* = 8 mice) and KO (*n* = 8 mice) hosts orthotopically transplanted with pancreatic cancer cells (**e**), after 6 hours of fasting. **f**, Area under the curve measurements for glucose tolerance test plots in **c**-**e**. **g**, Circulating glucose levels after 6 hours of fasting. WT, *n* = 3 mice; KO, *n* = 4 mice; KPC, *n* = 6 mice; KPCM, *n* = 6 mice; WT-host, *n* = 8 mice; KO-host, *n* = 8 mice. **h**, Post-prandial circulating insulin levels of KPC (*n* = 18 mice) and KPCM (*n* = 12 mice). **i**, Complete blood count of KPC (*n* = 5 mice) and KPCM (*n* = 4 mice) at humane endpoint. **j**, Complete blood count of WT (*n* = 4 mice) and KO (*n* = 4 mice) hosts 5 weeks after orthotopic transplantation of pancreatic cancer cells. WT host, *MsrA*^*+/+*^ host; KO host, *MsrA*^−*/*−^ host; KPC, *Kras*^*G12D*^*;p53*^*R172H*^*;PdxCre;MsrA*^*+/+*^; KPCM, *Kras*^*G12D*^*;p53*^*R172H*^*;PdxCre;MsrA*^−*/*−^*;* WT, *MsrA*^*+/+*^; KO, *MsrA*^−*/*−^. Error bars in this Fig. are means ± SDs. A two-tailed unpaired Student’s *t*-test was performed. N.S., not significant. Source data

**Extended Data Fig. 9:**
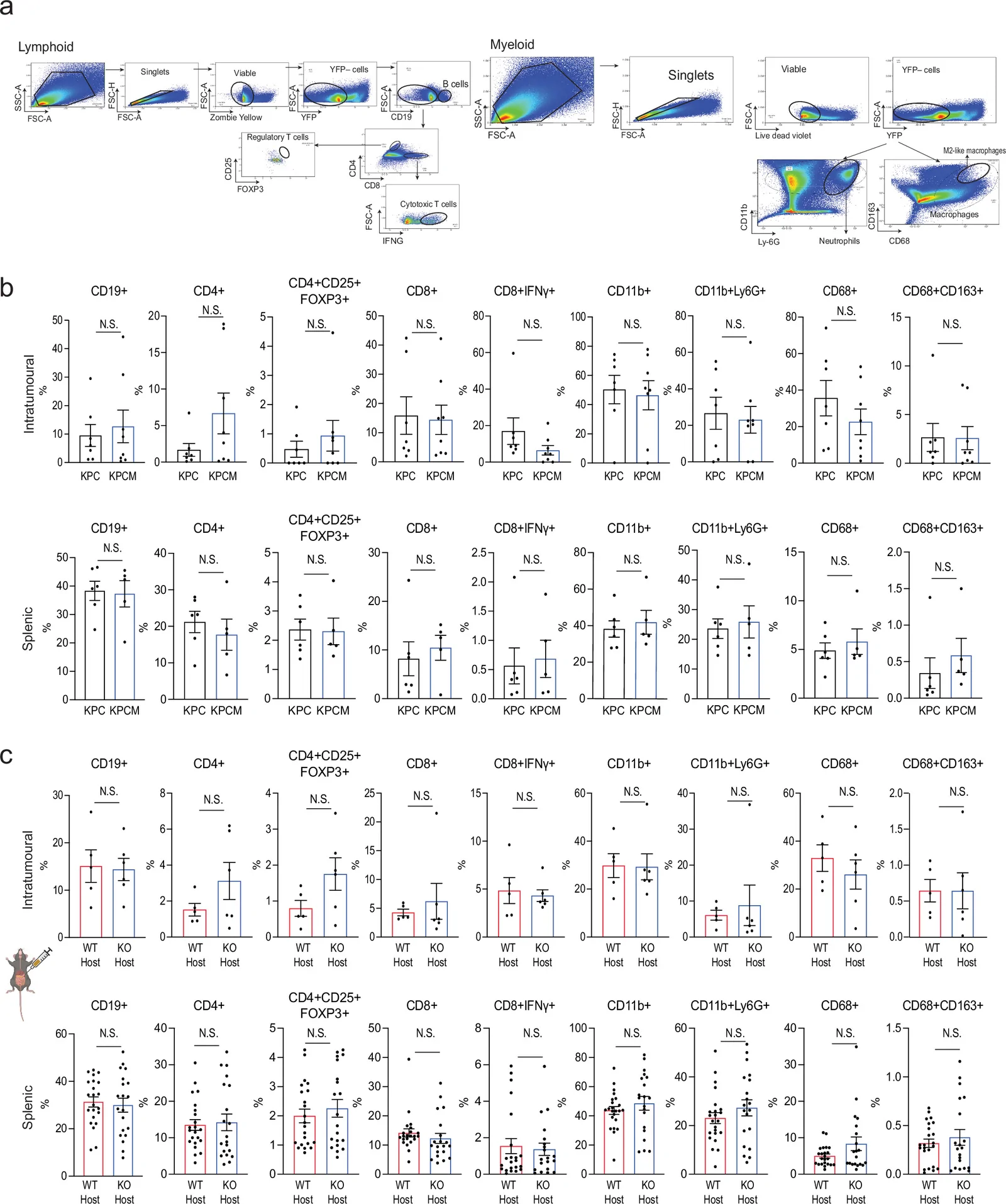
*MsrA* loss does not alter tumour-infiltrating or peripheral immune cell populations. **a**, Gating Strategy for flow cytometric analysis of immune cell composition. **b**, Flow cytometric analysis of intratumoural (KPC, *n* = 7 mice; KPCM, *n* = 8 mice) and peripheral (splenic) (KPC, *n* = 6 mice; KPCM, *n* = 5 mice) immune cell composition of KPC and KPCM mice with tumours 7-9 mm in diameter. **c**, Flow cytometric analysis of intratumoural (WT-host, *n* = 5 mice; KO-host, *n* = 6 mice) and peripheral (splenic) (WT-host, *n* = 23 mice; KO-host, *n* = 20 mice) immune cell composition of WT and KO hosts 5 weeks after orthotopic transplantation of pancreatic cancer cells. KPC, *Kras*^*G12D*^*;p53*^*R172H*^*;PdxCre;MsrA*^*+/+*^; KPCM, *Kras*^*G12D*^*;p53*^*R172H*^*;PdxCre;MsrA*^−*/*−^*;* WT host, *MsrA*^*+/+*^ host; KO host, *MsrA*^−*/*−^ host. Error bars in this Fig. are means ± SDs. A two-tailed unpaired Student’s *t*-test was performed. N.S., not significant. Source data

Although proinflammatory cytokines^56^ and insulin^57^ have been implicated in cancer cachexia, we did not observe consistent MSRA-dependent changes in circulating cytokines or adipokines levels in either autochthonous or orthotopic PDA models, regardless of tumor status (Extended Data Fig. 8a,b and Supplementary Table 5). Glucose tolerance (Extended Data Fig. 8c–f), fasting glucose (Extended Data Fig. 8g), insulin levels (Extended Data Fig. 8h), total blood cell counts (Extended Data Fig. 8i,j) and tumor-infiltrating and peripheral immune populations (Extended Data Fig. 9a–c) were also comparable in WT and KO mice in either PDA model.

**Extended Data Fig. 10:**
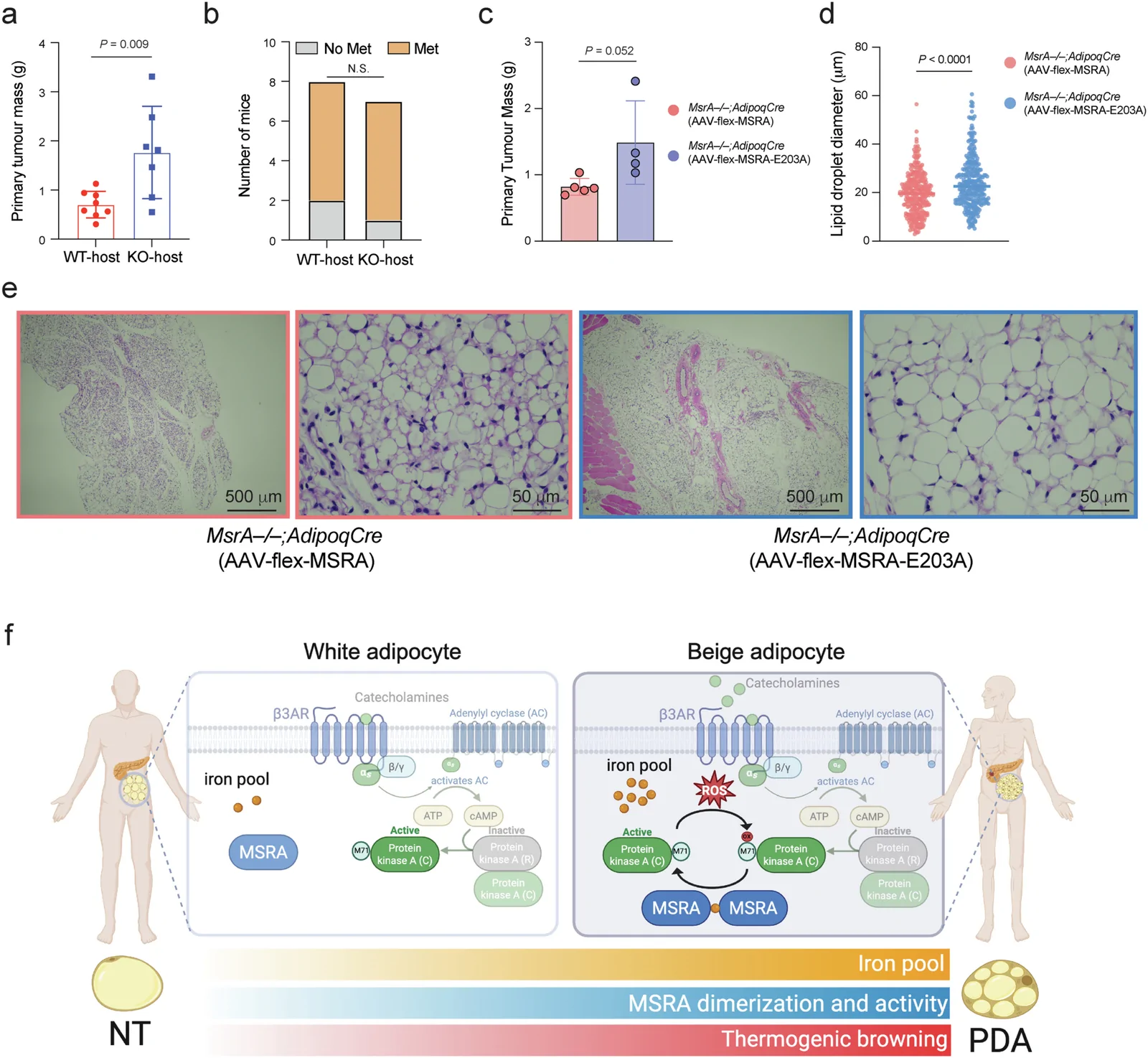
Adipose-specific restoration of wild-type MSRA reverses the survival benefit of *MsrA* deficiency in tumour-bearing mice. **a**, Tumour burden of moribund male mice after orthotopic transplantation of pancreatic cancer cells. WT-host, *MsrA*^*+/+*^ (*n* = 8 mice); KO-host, *MsrA*^−*/*−^ (*n* = 7 mice). **b**, Metastasis incidence of moribund male mice after orthotopic transplantation of pancreatic cancer cells. WT-host, *MsrA*^*+/+*^ (*n* = 8 mice); KO-host, *MsrA*^−*/*−^ (*n* = 7 mice). **c**, Tumour burden of moribund male mice after orthotopic transplantation of pancreatic cancer cells. *MsrA*^−*/*−^*;AdipoqCre* (AAV-flex-MSRA), *MsrA* null host expressing AdipoqCre transgene receiving AAV8 virus to conditionally express MSRA WT cDNA in all adipose tissues (*n* = 5 mice). *MsrA*^−*/*−^*;AdipoqCre* (AAV-flex-MSRA-E203A), *MsrA* null host expressing AdipoqCre transgene receiving AAV8 virus to conditionally express MSRA E203A mutant cDNA in all adipose tissues (*n* = 4 mice). **d**, Lipid droplet diameter measurements of subcutaneous WAT. 100 lipid droplets were quantified from each adipose tissue specimen. *MsrA*^−*/*−^*;AdipoqCre* (AAV-flex-MSRA), *n* = 3 mice; *MsrA*^−*/*−^*;AdipoqCre* (AAV-flex-MSRA-E203A), *n* = 3 mice. **e**, Representative hematoxylin & eosin images of subcutaneous WAT. Representative images are shown from three independent mice with similar results. **f**, Model of PDA-associated cachexia. β3AR, β3-adrenergic receptor; αs,β,γ G-protein subunits; cAMP, cyclic AMP; NT, non-tumour; PDA, pancreatic ductal adenocarcinoma. Error bars in this Fig. are means ± SDs. A two-tailed unpaired Student’s *t*-test was performed. N.S., not significant. Panel **f** created in BioRender. Chio, I. https://BioRender.com/w3iqo82 (2026). Source data

As adipose tissue loss correlates with reduced survival PDA patients^58,59^, we next asked whether MSRA-dependent adipose remodeling impacts survival in experimental settings. Notably, whereas KPC mice lost body mass in the days preceding death, this effect was attenuated in their *MsrA*-null counterparts (Fig. 5p). Moreover, the overall survival of tumor-bearing mice was significantly prolonged by *MsrA* loss in both autochthonous (Fig. 5q) and orthotopic (Fig. 5r) PDA models. This survival benefit occurred independently of primary tumor burden (Extended Data Fig. 10a) or metastatic incidence (Extended Data Fig. 10b). Furthermore, using the AAV-flex system for adipose-specific rescue, expression of WT MSRA—but not its iron-binding E203A mutant—completely reversed the survival advantage conferred by whole-body *MsrA* deletion (Fig. 5s), independent of primary tumor burden (Extended Data Fig. 10c). Moreover, tumor-bearing *MsrA*-null mice expressing AAV-encoded WT MSRA—but not the E203A mutant—showed progressive weight loss leading up to death (Fig. 5t), mirroring the pattern observed in KPC mice. Adipocyte diameters similarly declined only in the presence of WT MSRA (Extended Data Fig. 10d,e). Together, these results identify MSRA as an iron-dependent regulator of adipose remodeling required for the initiation of cachexia during pancreatic tumorigenesis (Extended Data Fig. 10f).

## Discussion

Although effective therapies to alleviate the lethal impact of cancer cachexia are still not available^1^, multiple studies show that adipose depletion precedes and may be required for muscle wasting^5,7,15,16,18^. Here, we identified an iron-dependent process that promotes adipose depletion by governing the redox state of proteinaceous methionine residues. This process entails sympathetic induction of β3AR signaling, labile iron accumulation, iron-dependent activation of MSRA and MSRA-catalyzed reduction of select protein substrates. Moreover, this β3AR–iron–MSRA axis is required not only for cachectic adipose browning but also for physiological browning during cold-induced thermogenesis. Importantly, disruption of the β3AR–iron–MSRA pathway in PDA-bearing mice mitigates the morbid outcomes of cancer cachexia, including adipose depletion, muscle wasting, weight loss and reduced survival.

Physiological adipose browning adipocytes is dependent on expansion of the labile iron pool to support the increased mitochondrial capacity^35^. However, elevated iron content can promote ROS production through the Fenton reaction, thereby rendering adipose proteins more susceptible to oxidative modifications. Here, we showed that iron levels also rise during cachectic adipose browning and the methionine proteome of WAT is more oxidized globally in PDA-bearing animals. Interestingly, MSRA expression is increased in an iron-dependent manner specifically in the browning WAT cells of tumor-bearing mice and the induction of both iron and MSRA are required for WAT browning.

Our results also showed that iron can directly bind a phylogenetically conserved EXXH motif of MSRA in a manner reminiscent of the ferritin superfamily proteins^39^. However, unlike the ferritin-like polypeptides, each of which possesses two EXXH motifs that coordinate iron intramolecularly, MSRA exploits its single EXXH motif to allow iron-mediated dimerization of two MSRA protomers. Dimerization in turn enables MSRA to function optimally in the iron-rich environment of thermogenic WAT cells by enhancing both its stability and its full catalytic activity. This model may explain why MSRA is largely dispensable for normal development^52^ but essential within the iron-rich oxidative intracellular environment of browning adipocytes. MSRA had often been viewed as a constitutive housekeeping enzyme that broadly maintains redox homeostasis, with overt phenotypes emerging only when mice are challenged with strongly oxidizing agents such as paraquat^60^. Our results revise this view by showing that MSRA is uniquely required in defined physiological and pathological contexts, such as cold-induced thermogenesis and cancer cachexia.

As MSRA is required for cancer cachexia, its relevant substrates should have redox-sensitive methionines that are preferentially reduced in the adipose tissues of tumor-bearing individuals. Our unbiased chemoproteomic screen identified seven proteins, including PKAC, with these properties. Thus, we propose that MSRA helps to orchestrate adipose remodeling by coordinately modulating the functions of multiple substrates. The biological impact of methionine oxidation on each of these substrates will likely vary depending on its particular structural and biochemical properties. Given the known role of PKA in β3AR-stimulated thermogenesis, we examined how methionine oxidation of PKAC affects its enzymatic activity and its impact on adipose browning. Structurally, cAMP activates PKA signaling by binding the PKAR regulatory subunit, which allows the PKAC subunit to freely equilibrate between its open (inactive) and closed (active) conformations^48^. Interestingly, the sole MSRA-regulated residue of PKAC is a highly conserved methionine (M71) that lies adjacent to an ATP-binding residue (K72) within the PKAC catalytic cleft. Our combined in silico, in vitro and in vivo analyses argue that oxidation of PKA diminishes ATP binding, whereas its reduction by MSRA restores full kinase activity. These data imply that PKA activation is, at least during β3AR-induced adipose browning, a two-step process involving cAMP-mediated release of PKAC followed by MSRA-mediated preservation of its reduced redox state.

Understanding the differences between physiological browning (a reversible process associated with metabolic health^8,14^) and cachectic browning (irreversible even in the face of increased caloric intake^1^) should allow for the development of nontoxic therapies for cancer cachexia. As adipose atrophy preceded skeletal muscle wasting in both genetically engineered and transplant PDA models, our data support the notion that WAT browning is an early and possibly initiating event in the pathogenesis of cancer cachexia^7,15,16,18^. Thus, as an essential mediator of adipose atrophy, the β3AR–iron–MSRA axis is a promising target for therapeutic mitigation of cancer cachexia.

## Methods

### Materials and reagents

Complete reagent names, suppliers, catalog numbers and concentrations used are compiled in Supplementary Table 6. Media, sera, antibiotics, freezing medium, sequences and processing enzymes were used according to manufacturers’ instructions without deviations unless noted below.

### Human samples

#### Rapid autopsy samples

Visceral adipose tissue samples were obtained from individuals previously diagnosed with PDA through the University of Nebraska Medical Center’s Rapid Autopsy Program (UNMC RAP) for Pancreas under IRB 091-01. Noncancerous tissues were collected in a similar manner through the UNMC Normal Organ Recovery Program in collaboration with LiveOn Nebraska. To ensure specimen quality, organs were isolated within 3 h postmortem and the samples were either flash-frozen in liquid nitrogen or immediately placed in formalin for fixation. For Columbia University samples, visceral adipose tissue was freshly collected from participants undergoing laparoscopic cholecystectomy or resection of clinical stage I or II PDA or placement of port-a-catheters for systemic treatment in participants with clinical stage III or IV PDA under IRB-AAAU7458. Informed consent from participants was obtained for the use of the samples collected in the study.

### Animal models

*Trp53*^+/LSL-R172H^, *Kras*^+/LSL-G12D^ and *Pdx1-Cre* strains in C57Bl/6NJ background were interbred to generate compound mutant (KPC)^22^ mice. *MsrA*^−/−^ and *AdipoqCre* mice in C57BL/6NJ were obtained from Jackson Laboratory. *MsrA* KO was interbred with KPC to generate the compound mutant KPCM. Orthotopic engraftment of mouse PDA cells was conducted as described previously^29^. In brief, mice were anesthetized using isoflurane and subcutaneously administered 5 mg kg^−1^ carprofen. A total of 100,000 cells were transplanted to the parenchyma of the pancreas. The abdominal wall was sutured with absorbable Vicryl sutures and the skin was closed with wound clips. Tumor-bearing mice were subjected to high-contrast ultrasound imaging using the Vevo 2100 system with an MS250, 13–24-MHz scanhead (Visual Sonics) to monitor tumor volume intravitally. Mice were housed in ventilated cages in a temperature-controlled, specific-pathogen-free animal facility with a 12 h light–dark cycle. Mice were fed a standard rodent diet ad libitum. All animal experiments were conducted in accordance with procedures approved by the Institutional Animal Care and Use Committee (IACUC) at Columbia University (AC-AABK5554). The tumor burden endpoints (tumor volume no greater than 2,000 mm^3^, morbidity, labored breathing or impingement of the animal’s ability to ambulate, eat and drink) allowed by our IACUC were not exceeded.

### Indirect calorimetry system and body composition

Mice were acclimated in metabolic chambers (Promethion Core, Sable Systems International) for 4 days before the start of the recordings. Mice were continuously recorded for 3 days, with measurements taken every 30 min, including food intake, water intake and ambulatory activity (in *x* and *z* axes) using rodent metabolic cages. Values were adjusted by body mass where mentioned. In addition, food intake was continuously determined by integrating weighing sensors fixed at the top of the cage, from which the food containers were suspended, into the sealed cage environment. For longitudinal studies, food intake was measured manually by daily measurements of the changes in chow pellet mass. Ground food was extracted from cage beddings with a strainer. Body composition was measured intravitally by magnetic resonance imaging (MRI; EchoMRI, Echo Medical Systems).

### MRI to measure body fat

MRI experiments were carried out using a Bruker BioSpec 9.4 Tesla preclinical scanner equipped with the Paravision (PV.7.0.0) software platform (Bruker). Mice were anesthetized with 1–2% isoflurane mixed with medical air and delivered through a nose cone. During the procedure, the isoflurane concentration was adjusted to maintain a respiratory rate of 40–80 breaths per min, monitored with a respiration pillow connected to a monitoring system (SA Instruments). The mice were placed in a prone position on the animal bed inside a circular polarized birdcage coil with an inner diameter of 72 mm. Images were acquired using the rapid acquisition with relaxation enhancement sequence with a fat–water separation technique. The imaging parameters were set as follows: TR = 1,448 ms, TE = 26 ms and a resolution of 265 × 265 μm^2^. Coronal images were obtained with a slice thickness of 1 mm, consisting of 24 slices to cover the entire body of the mouse. A 140° angle between fat and water magnetization was used to produce out-of-phase images. Separate images of fat and water were generated and a recombined fat–water image was reconstructed to provide a clear depiction of the mouse’s anatomy. The data were imported into Analyze Direct 14.0 software. Initial preprocessing steps included contrast enhancement to improve the clarity of the fat tissue boundaries. Segmentation was carried out to differentiate and quantify subcutaneous and visceral fat. Using the thresholding tool, intensity thresholds were set to identify fat tissues on the basis of their specific signal characteristics. The segmentation process was further refined using region-growing algorithms to ensure accurate delineation of fat boundaries. Manual adjustments were made where necessary to correct any misclassifications. Following segmentation, the volume of the identified fat compartments was calculated. The software’s volume rendering module was used to create three-dimensional reconstructions of the fat distribution. Volume measurements were obtained by summing the voxel volumes of the segmented regions. The total fat volume, as well as the volumes of subcutaneous and visceral fat, was recorded for each subject.

### Temperature measurements

To monitor local sWAT temperature longitudinally, implantable transmitters were implanted directly above the inguinal sWAT. Temperature was recorded daily at 4 p.m. using BMDS reader programmers. Core body temperature was measured daily at 4 p.m. using a ThermoWorks rectal probe. The compact thermal camera (FLIR C5) was used to measure the dorsal temperatures of individual mice and the collected images were processed using FLIR thermal studio.

### Immunohistochemical and immunofluorescence analysis

Tissues were fixed in 10% neutral buffered formalin, embedded in paraffin and sectioned to a thickness of 5 μm.

Tissue sections were unwaxed and prepared for antigen retrieval following the methodology previously described^61^. Antigen retrieval was conducted using citrate buffer pH 6.0. Slides were washed three times with PBS and blocked with blocking solution (PBS supplemented with 0.1% Triton X-100, 1% BSA and 2% donkey serum) for 1 h at room temperature. Sections were incubated overnight at 4 °C with primary antibodies previously diluted in blocking solution as previously described^33^. Sections were washed three times with PBS and stained with secondary antibodies at room temperature for 1 h. Sections were washed three times with PBS and coverslips were mounted in ProLong Diamond antifade mountant. All fluorescence images for full tissue scans were acquired at the Dana-Farber Cancer Institute Molecular Imaging Core using LASX Office version 1.4.5 27713 on a Leica THUNDER Imager wide-field microscope. Images were managed using Fiji^62^. For COX4 and TNAP colocalization analysis, a total of 334 images were converted to masks using CellProfile^63^. The segmented masks and original images were then fed into CellProfiler to calculate the correlation between TNAP and COX4.

### Adipocyte lipid droplet and myocyte diameter analysis

Hematoxylin and eosin (H&E)-stained slides were scanned at ×40 using the Leica SCN400 and the diameters of 100 lipid droplets and 100 myocytes were measured per slide using the QuPath software (version 0.5.1).

### Cell culture

Primary adipocytes were isolated from newborn mice using a published protocol^53^. In brief, white and brown adipose depots were isolated from pups on postnatal day 1. Tissues were dissociated with 15 mg ml^−1^ collagenase I for 30 min at 37 °C with mixing. The digest was passed through a 100-μm cell strainer and plated. When preadipocytes became confluent, differentiation was performed with 10% DMEM, 170 nM insulin, 1 mM dexamethasone, 0.5 mM 3-isobuthyl-1-methylxanine and 2 μM rosiglitazone. All primary cultures were genotyped and authenticated by short tandem repeat analysis to identify misidentified, cross-contaminated or genetically drifted cells. All cells cultured in our laboratory were screened for *Mycoplasma* contamination monthly. Unless stated otherwise, all cells were cultured in DMEM, supplemented with 10% FBS and 1% penicillin–streptomycin (PS). All cells were cultured at 37 °C with 5% CO_2_.

### CRISPR–Cas9-mediated gene deletion and viral transduction

White preadipocytes were transduced with lentivirus expressing Cas9 and sgRNAs. Single sgRNAs were cloned by annealing two DNA oligos and T4 DNA ligation into a BsmB1-digested LRC2.1T as described previously^64^. The design principle of sgRNA was based on previous reports: (1) sgRNAs with the predicted low off-target effect^65^ and (2) sgRNAs targeting the functional protein domain region^64^, for which sequence information was retrieved from the National Center for Biotechnology Information conserved domains database. Lentiviruses were produced in 293T cells coexpressing the packaging vectors (pPAX2 and VSVG) and ecotropic pBabe-puro retroviruses were produced in Phoenix-ECO cells. Viruses were concentrated with LentiX concentrator and resuspended with DMEM supplemented with 10% FBS and 1% PS at 10× concentration. Cells were transduced with 5× concentrated viruses by spinoculation at 600*g* for 45 min at room temperature.

### AAV generation and transduction

Plasmids: mMSRA WT and mutant (C72S and E203A) open reading frames were cloned into pAAV-Ef1a or pAAV-Ef1a-FLEX transfer plasmids using Q5 DNA polymerase and NEBuilder HIFI DNA assembly and DNA was propagated using NEB Stable cells. Endotoxin-free plasmid DNA was purified using Maxi Fast ion plasmid columns. rAAVs were produced using 293T cells transfected with pHelper, AAV8 capsid and AAV transfer plasmids using polyethylenimine and cultured as previously described^66^. Cell lysates and medium were isolated 72 h after transfection and viral particles in the medium were precipitated using 40% polyethylene glycol. The cell pellet was subjected to three rounds of liquid nitrogen snap-freezing with 55 °C thawing, followed by trituration using an 18G needle. Lysates and precipitated medium pellets were combined and resuspended in 1× GB buffer (50 mM Tris pH 7.6, 150 mM NaCl and 10 mM MgCl_2_), treated with Benzonase at 37 °C for 1 h and clarified by centrifugation at 3,000*g*. Supernatants were purified with iodixanol step gradients (15%, 25%, 40% and 58%). The iodixanol gradient was spun at 360,000*g* for 1 h at 18 °C in a Ti70 rotor. Fractions between the 58/40 and 40/25 interface were collected and analyzed using PAGE to identify clean AAV fractions. These fractions were pooled, exchanged with PBS and concentrated using Amicon filters. Viral samples treated with DNase I were used for qPCR to estimate titers. For local expression, 1 × 10^11^ viral genome copies (vg) were administered into the inguinal fat pad. For adipose-specific (*MsrA*^−*/*−^;*AdipoqCre* recipients) expression, 3 × 10^11^ vg of virus were introduced intraperitoneally.

### Lipidomic analysis

Lipids were extracted from fecal samples as previously described^67^. Briefly, 500 μl of ice-cold methanol containing 0.01% (w/v) BHT was added to 30 mg of fecal material in a tube containing 2.8-mm ceramic beads. The samples were homogenized and then centrifuged at 500*g* for 3 min at 4 °C to collect the supernatant. Next, 850 μl of methyl *tert*-butyl ether was added to the supernatant, vortexed for 1 min and incubated at −20 °C for 2 h. Subsequently, 200 μl of water was added, vortexed again for 1 min and incubated at −20 °C for 20 min to induce phase separation. Samples were then centrifuged at 1,250*g* for 20 min at 4 °C and 700 μl of the upper (lipid-containing) layer was collected and evaporated to dryness under a gentle stream of nitrogen. The dried lipid extract was resuspended in 2-propanol, acetonitrile and water (4:4:1, v/v/v) containing 10 μl of Mouse SPLASH Lipidomix internal standard before liquid chromatography (LC)–MS analysis. Lipidomic profiling was performed on a SYNAPT G2-Si Q-ToF MS instrument coupled to an ultrahigh-performance LC I-Class system, as previously described^68^.

### Methionine reactivity profiling

Methionine reactivity profiling was performed using Ox-Alk labeling followed by click chemistry, streptavidin enrichment, on-bead digestion and diaPASEF MS. Briefly, adipose tissues were homogenized in RIPA buffer and clarified by centrifugation, followed by quantification of total protein. Proteins were labeled with Ox-Alk, conjugated to biotin azide by copper-catalyzed click chemistry, enriched using streptavidin beads, digested with trypsin and analyzed by LC–MS/MS on a timsTOF Pro 2 platform. Raw DIA data were processed using Spectronaut and differential methionine reactivity was analyzed in R using limma/Normalyzer and downstream pathway enrichment tools. The full protocol is available on protocols.io^69^. The database used was *Mus* *musculus* (version 2022; 21,992 entries), downloaded from UniProt. Carbamidomethyl on cysteine was specified as the fixed modification, while oxidation on methionine was specified as the variable modification. Peptides that passed the 1% *q*-value cutoff were used to calculate the major group quantities with the MaxLFQ method. C_14_H_25_N_5_O_3_ (*m*/*z* = 311.1957) was used as the variable modification for oxaziridine-labeled methionine residues. The limma package (version 3.54.2) was used to compare protein expression levels, while the LOESS (limma package) normalization method through Normalyzer^70^ was used to compare methionine reactivity, identifying differentially expressed proteins with an adjusted *P* value < 0.05. To minimize potential signal from nonadipocyte proteins, we filtered the dataset using publicly available adipocyte proteomic profiles (PXD039523)^71^. Following the differential expression analysis, we performed gene set enrichment analysis (GSEA) using annotated gene sets from the Molecular Signatures Database (MSigDB) to determine whether a predefined set of genes shows statistically significant, concordant differences between two biological states. Fast GSEA was performed with the fgsea package (version 1.28.0). We then visualized the pathway analysis results through enrichment plots, with an adjusted *P* value < 0.05 applied throughout. All analyses were performed using R Statistical Software (version 4.2.2; R Core Team 2022).

### ICP–MS

Total elemental abundance was measured by ICP–MS. Briefly, mouse tissues were weighed and digested by microwave-assisted nitric acid digestion and then diluted with internal standards before analysis. Serum and MSRA immunoprecipitate samples were diluted in matrix-matched acid-based solutions containing internal standards. Copper, zinc, iron and selenium were quantified using an Agilent 8900 ICP–MS/MS with external matrix-matched calibration and appropriate gas modes for each element. Method blanks and certified reference materials were included for quality control and accuracy assessment. The full protocol is available on protocols.io^72^.

### Global transcriptome analysis

Total RNA (RNA integrity number > 7) was extracted from adipose tissues and poly(A) pulldown was performed to enrich mRNAs. Library construction was performed using Illumina TruSeq chemistry, with the final PCR step modified using the KAPA HiFi HotStart ready mix. Libraries were sequenced using Element AVITI at the Columbia Genome Center. Samples were multiplexed in each lane, yielding a targeted number of paired-end 75-bp reads for each sample. Bases2fastq (version 1.4.0.833301531) was used to convert BCL data to fastq format, with adaptor trimming. Pseudoalignment to a kallisto index was created from transcriptomes (mouse:GRCm38.p6) using kallisto (0.44.0). Differentially expressed genes were analyzed using DESeq2 R packages designed to test differential expression between two experimental groups from RNA-seq counts data. GSEA was performed using the clusterProfiler package (version 4.10.0).

### RT–qPCR

TRIzol was used for RNA extraction. cDNA was synthesized with TaqMan reverse transcription (RT) reagents. All targets were amplified (40 cycles) using gene-specific SybrGreen primers and Taqman primers and probe sets (Applied Biosystems) on a QuantStudio5 real-time PCR instrument (Applied Biosystems). Relative gene expression was quantified using the ΔΔ*C*_*t*_ method with QuantStudio real-time PCR software (version 1.1; Applied Biosystems). Expression levels were normalized to *18S* and *Hprt*. Taqman probes and SybrGreen primers are described in Supplementary Table 6.

### Protein extraction and immunoblotting

Tissues and cells were lysed with either RIPA or Native/IP lysis buffer (50 mM HEPES pH 7.5, 140 mM NaCl and 1% Triton X-100) containing protease inhibitor (cOmplete) and phosphatase inhibitor (PhosStop). For adipose tissues, snap-frozen tissues were placed in lysis buffer without Triton X-100 and homogenized until clear. Homogenates were centrifuged at 6,000*g* for 15 min at 4 °C. After removal of the top white lipid layer, Triton X-100 was added to the bottom aqueous layer to a final concentration of 1% (v/v). Nonadipose tissues were homogenized in 3.0-mm zirconium bead-filled tubes containing 500 µl of lysis buffer. Adipocyte cultures were isolated in lysis buffer on ice using cell lifters. All lysates were clarified by centrifugation at 12,000*g* for 15 min at 4 °C. For SDS–PAGE analysis, lysates in RIPA buffer were separated on a 12% Bis–Tris SurePAGE gel, transferred onto nitrocellulose membrane and incubated with antibodies. For native PAGE analysis, lysates in native/IP buffer were separated on Novex tricine gels, transferred onto nitrocellulose membrane and incubated with anti-MYC tag antibody. Because migration under native conditions depends on protein charge, conformation, oligomeric state and shape, apparent molecular weight cannot be inferred reliably from standard protein ladders. Therefore, native immunoblot panels are shown without molecular weight markers, and the two detected MSRA species are referred to as lower and upper migrating bands (Fig. 2i,k,l and Extended Data Fig. 3f,h). Antibodies are described in Supplementary Table 6.

### Immunoprecipitations

Anti-MYC tag magnetic beads were washed three times with IP lysis buffer and incubated overnight with clarified lysates at 4°C (150 µl of beads per 1 mg of lysate). Beads were then washed four times with IP lysis buffer, eluted with 100 µl of glycine–HCl buffer (0.1 M, pH 2.2) and immediately neutralized with 15 µl of Tris buffer (1 M, pH 8.0).

### TurboID proximity ligation

Cells were incubated with 50 µM biotin in culture for 30 min, washed three times with PBS and collected into a microtube. Cell pellets were resuspended in 8 M urea Tris buffer (50 mM, pH 8.0), followed by reduction (10 mM DTT at 37 °C for 1 h), alkylation (55 mM iodoacetamide at 37 °C for 1 h in the dark), 1:8 dilution with 50 mM Tris buffer (1 M, pH 8.0) and digestion (1 mM CaCl_2_ and sequence-grade trypsin at 1:50 w/w at 37 °C overnight). The digested samples were mixed with streptavidin magnetic beads (200 µl of beads per 1.5 mg of starting lysate) and incubated at room temperature with rotation for 1 h. The beads were removed using a magnetic rack, washed twice with 2 M urea in 50 mM ABC and eluted with 80% acetonitrile, 0.2% trifluoroacetic acid (TFA) and 0.1% formic acid at 60 °C for 5 min. The eluted peptides were collected into a new microtube, desalted using Pierce peptide desalting spin columns and analyzed by MS.

### In silico structural analyses

AlphaFold3 predictions were performed using the Google DeepMind AlphaFold3 Server. The sequence from *M*. *musculus* was used for both MSRA (UniProt Q9D6Y7) and PKAC (UniProt P05132) structure prediction. The pKa of residue C72 of MSRA was predicted using the PropKa webserver. The AlphaFold-predicted structure of an MSRA monomer or dimer was used as the input structure. PDBePISA was used to analyze the SASA of residue M71 in PKAC. The input structures used are indicated in the figure legends. Protein stability changes in PKAC were predicted using DynaMut2. The corresponding structure input used for the prediction is indicated in the figure legends.

### TNP-ATP binding assay and *K*_d_ calculations

The 293T cells transfected with pcDNA3.1 constructs encoding PKA-WT-c-MYC, PKA-M71E-c-MYC or PKA-K72H-c-MYC were lysed in native/IP buffer, incubated on ice for 30 min and clarified by centrifugation (12,000*g*, 15 min, 4 °C). Next, 2 mg of lysate was incubated overnight at 4 °C with 100 µl of Pierce anti-c-MYC magnetic beads. Beads were washed three times with native/IP buffer and bound proteins were eluted with 0.5 mg ml^−1^ c-MYC peptide in 1× TBS at 37 °C for 15 min in a thermomixer (750 rpm). TNP-ATP binding assays were performed as described previously^73^. Briefly, 10 µl of eluted proteins were mixed with 5 µM TNP-ATP in Tris-HCl buffer (50 mM Tris-HCl and 50 mM KCl, pH 7.2) in a 96-well black plate. Fluorescence was recorded with excitation at 410 nm and an emission scan from 500–600 nm. Competitive binding was assessed by adding 0.4 mM ATP. For saturation assays, buffer alone or immunoprecipitated proteins were incubated with 1, 5, 10, 25, 50 or 100 µM TNP-ATP and fluorescence was recorded with excitation at 410 nm and emission at 560 nm. The relative fluorescence units (RFU) of each sample were baseline-corrected by subtracting the average RFU of the buffer-only control at each corresponding concentration. All samples were measured in triplicate. Dissociation constants (*K*_d_) were determined by nonlinear curve fitting using GraphPad Prism (one-site-total binding model).

### Fluorometric cAMP assay

cAMP levels were measured using a cAMP assay kit according to the manufacturer’s instructions. A total of 75 μl of lysates (containing 20 μg of protein) or cAMP standards (0, 0.3, 1, 3, 10, 30, 100 and 300 nM) were added to anti-cAMP antibody-coated wells of a black 96-well plate and incubated for 10 min. Then, 25 μl of 1× HRP–cAMP conjugate working solution was added to each well. After 2 h of incubation on a plate shaker, the wells were aspirated and washed four times with 200 μl of 1× wash solution. Next, 100 μl of AbRed working solution was added to each well and fluorescence was measured in top-read mode (excitation: 540 ± 9 nm, emission: 590 ± 15 nm). A standard curve was generated using a four-parameter logistic regression model and sample cAMP concentrations were calculated accordingly.

### Recombinant protein synthesis

The genes encoding human *MSRA* or mouse *MsrA*, m*MsrA*^E203A^ and m*MsrA*^AxxA^ were cloned into the pET30a(+) expression vector with an N-terminal 6× His tag followed by the tobacco etch virus (TEV) protease recognition sequence (ENLYFQG). The plasmids were transformed into BL21 StarTM (DE3) chemically competent *Escherichia* *coli* and grown in lysogeny broth for 16 h at 15 °C. Expressed proteins were purified through a Ni-NTA HisTrap column. To remove the His tag, TEV protease was added at a protease-to-substrate ratio of 1:50 (w/w) into His-tagged MSRA in 150 mM NaCl, 0.5 mM TCEP and 20 mM Tris-HCl (pH 7.5). The reaction mixture was incubated at 4 °C overnight. The cleavage reaction mixture was then applied to a second nickel-affinity column to enrich for the untagged target protein, followed by concentration and purification by SEC using a Superdex 200 Increase column (10/300) (Cytiva).

### Reconstitution of recombinant MSRA with iron

MSRA protein solution, Mohr’s salt (ammonium iron(II) sulfate) and Milli-Q water were degassed by applying 3–5 vacuum cycles, before maintaining in an anaerobic glove box maintained at <3 ppm oxygen. Inside the glove box, Mohr’s salt was freshly dissolved in Milli-Q water, added to the protein solution at defined molar equivalents and incubated for 2 h. After incubation, samples were aerated by gentle pipetting outside the glove box to facilitate oxo-bridge formation at the MSRA iron-binding site.

### SEC–MALS

Chromatography experiments were performed using a Superdex 200 Increase 5/150 GL (Cytiva) column attached to an Akta Pure fast protein LC (FPLC) system (GE Healthcare) coupled to inline static light scattering (Dawn Heleos II, Wyatt Technology), differential refractive index (Optilab rEX, Wyatt Technology) and UV detection. The SEC flow rate was set to 0.5 ml min^−1^ using a buffer consisting of 150 mM NaCl and 20 mM HEPES pH 7.5. Every SEC run was monitored using the Akta Pure UV detector at 280 nm. The eluted samples were collected in 500-μl fractions for further biochemical analyses.

### MSRA activity assay

SEC-purified specimens were added to 50-µl reaction mixtures containing 5 mM L-methionine, 5 mM L-methionine sulfoxide, 30 mM KCl, 10 mM MgCl_2_ and 20 mM DTT at 37 °C. At the end of the reaction, 300 µl of Milli-Q water was added and MSRA protein was removed using Amicon Ultra 0.5 3-kDa centrifugal filters at 12,000*g* for 20 min. L-Methionine and L-methionine sulfoxide levels in the filtrate were quantified by reverse-phase high-performance LC with UV detection at 214 nm (Agilent 1260 Infinity II). Solvent A was water with 0.1% TFA and solvent B was acetonitrile with 0.1% TFA. Separation was performed at a flow rate of 1 ml min^−1^ using the following linear gradient: 0–2 min, 0% B; 2–32 min, 0–70% B; 32–33 min, 70–100% B; 33–35 min, 100% B; 35–36 min, 100–0% B. Retention times for L-methionine and L-methionine sulfoxide were 3.8 min and 2.1 min, respectively.

### Ox-Alk and iodoacetamide-alkyne labeling for immunoblot analysis

First, 100 μM Ox-Alk or iodoacetamide-alkyne was incubated with lysates for 1 h at 25 °C, followed by copper click reactions using 200 μM biotin-(PEG)_3_-azide and trichloromethane–methanol precipitation. The precipitated proteins were solubilized in 2% SDS in PBS and diluted to 0.1% SDS for biochemical analyses.

### Immunological analyses

#### Total blood count

First, 20–50 µl of blood was placed into EDTA-coated blood collection tubes and mixed to prevent clotting. Whole blood count was performed using Hemavet 950 (Drew Scientific).

#### Tissue immune profiling by flow cytometry

Tumor samples were dissociated with 2.5 mg ml^−1^ collagenase D, 0.5 mg ml^−1^ Liberase TL, 0.2 mg ml^−1^ DNAse I and Y27632 in advanced DMEM/F12 medium with 5% FBS. Spleens were homogenized using a 70-μm cell strainer in RPMI. Red blood cells were lysed using ACK lysis buffer. Cells were washed with PBS and suspended in FACS buffer (PBS, 2% FBS and 0.2% 0.5 M EDTA) followed by Fc blocking (anti-CD16/CD32) and antibody staining. Samples were recorded using the Cytek Aurora and subsequently analyzed using the FlowJo software.

### Circulating cytokine and hormone analyses

Whole blood was collected into lithium heparin tubes by cardiac puncture at necropsy. Samples were kept at room temperature for 1 h to clot and then centrifuged at 2,000*g* for 15 min at 4 °C. The serum was then collected and snap-frozen on dry ice. Prepared samples were assayed using the procedures described in the R&D Systems Proteome Profiler mouse adipokine array kit, the Proteome Profiler mouse XL cytokine array and the mouse insulin ELISA kit according to the manufacturer’s instructions.

### Oral glucose tolerance test

Following a 6-h fast, mice were administered glucose (2 g kg^−1^ body weight) by oral gavage. Blood glucose levels were measured at baseline and at 10, 20, 30, 60, 90 and 120 min using a glucometer.

### Statistics and reproducibility

Biochemical experiments in vitro were repeated at least three times, with the number of replicates increased as needed on the basis of effect size. We estimated the sample size on the basis of the sample mean and variance. No statistical method was used to predetermine the sample size. Data distribution was assumed to be normal for all datasets presented but this was not formally tested. Individual data points are shown for all results. No animals or samples were excluded from any analysis. Animals were randomly assigned to groups for in vivo studies; no formal randomization method was used when assigning animals to treatment. All western blotting experiments with quantification were performed a minimum of three times with independent biological samples and analyzed by ImageJ 1.52q. Investigators were blinded to group allocation during data analysis. Statistical analyses were performed using GraphPad Prism 8. All tests and *P* values are provided in the corresponding figures or figure legends.

### Inclusion and ethics statement

All contributors to this study who met the authorship criteria set by Nature Portfolio journals have been included as authors, reflecting their essential roles in the design and execution of the research. Roles and responsibilities were established among collaborators prior to the commencement of the study. The research was conducted without severe restrictions or prohibitions in the researchers’ environment and does not pose any risk of stigmatization, incrimination, discrimination, or personal harm to participants.

### Reporting summary

Further information on research design is available in the Nature Portfolio Reporting Summary linked to this article.

## Supplementary information

Reporting Summary

Supplementary Tables Supplementary Tables 1–6.

## Source data

Source Data Fig. 1 Unprocessed western blots and/or gels.

Source Data Fig. 1 Statistical source data.

Source Data Fig. 2 Unprocessed western blots and/or gels.

Source Data Fig. 2 Statistical source data.

Source Data Fig. 3 Unprocessed western blots and/or gels.

Source Data Fig. 3 Statistical source data.

Source Data Fig. 4 Unprocessed western blots and/or gels.

Source Data Fig. 4 Statistical source data.

Source Data Fig. 5 Unprocessed western blots and/or gels.

Source Data Fig. 5 Statistical source data.

Source Data Extended Data Fig. 1 Unprocessed western blots and/or gels.

Source Data Extended Data Fig. 1 Statistical source data.

Source Data Extended Data Fig. 2 Unprocessed western blots and/or gels.

Source Data Extended Data Fig. 2 Statistical source data.

Source Data Extended Data Fig. 3 Unprocessed western blots and/or gels.

Source Data Extended Data Fig. 3 Statistical source data.

Source Data Extended Data Fig. 4 Unprocessed western blots and/or gels.

Source Data Extended Data Fig. 4 Statistical source data.

Source Data Extended Data Fig. 5 Unprocessed western blots and/or gels.

Source Data Extended Data Fig. 5 Statistical source data.

Source Data Extended Data Fig. 6 Unprocessed western blots and/or gels.

Source Data Extended Data Fig. 6 Statistical source data.

Source Data Extended Data Fig. 7 Unprocessed western blots and/or gels.

Source Data Extended Data Fig. 7 Statistical source data.

Source Data Extended Data Fig. 8 Statistical source data.

Source Data Extended Data Fig. 9 Statistical source data.

Source Data Extended Data Fig. 10 Statistical source data.

## Data Availability

RNA sequencing data were deposited to the Gene Expression Omnibus under accession code GSE274804. Proteomics data were deposited to the ProteomeXchange Consortium through the PRIDE repository with the dataset identifiers PXD054899, PXD063328 and PXD064378 for expression proteomics, chemoproteomics and proximity ligation (TurboID) proteomics, respectively. All data supporting the findings of this study are available from the corresponding author on reasonable request. Source data are provided with this paper.
