# Supplementary material for: An iron-regulated methionine redox axis governs adipose browning and cancer cachexia
Source: Nat Cancer. 2026 Sep 22;7(10):1514–32. doi: 10.1038/s43018-026-01234-y (PMC13694233; doi:10.1038/s43018-026-01234-y)
Supplement: Supplementary file 6 [file 43018_2026_1234_MOESM1_ESM.pdf]

nature portfolio

Corresponding author(s): Iok In Christine Chio

Last updated by author(s): Aug 4, 2026

Reporting Summary

Nature Portfolio wishes to improve the reproducibility of the work that we publish. This form provides structure for consistency and transparency in reporting. For further information on Nature Portfolio policies, see our Editorial Policies and the Editorial Policy Checklist.

Statistics

For all statistical analyses, confirm that the following items are present in the figure legend, table legend, main text, or Methods section.

n/a	Confirmed	
☐	☒	The exact sample size (*n*) for each experimental group/condition, given as a discrete number and unit of measurement
☐	☒	A statement on whether measurements were taken from distinct samples or whether the same sample was measured repeatedly
☐	☒	The statistical test(s) used AND whether they are one- or two-sided<br>*Only common tests should be described solely by name; describe more complex techniques in the Methods section.*
☒	☐	A description of all covariates tested
☐	☒	A description of any assumptions or corrections, such as tests of normality and adjustment for multiple comparisons
☐	☒	A full description of the statistical parameters including central tendency (e.g. means) or other basic estimates (e.g. regression coefficient) AND variation (e.g. standard deviation) or associated estimates of uncertainty (e.g. confidence intervals)
☐	☒	For null hypothesis testing, the test statistic (e.g. *F*, *t*, *r*) with confidence intervals, effect sizes, degrees of freedom and *P* value noted<br>*Give P values as exact values whenever suitable.*
☒	☐	For Bayesian analysis, information on the choice of priors and Markov chain Monte Carlo settings
☒	☐	For hierarchical and complex designs, identification of the appropriate level for tests and full reporting of outcomes
☒	☐	Estimates of effect sizes (e.g. Cohen's *d*, Pearson's *r*), indicating how they were calculated

Our web collection on statistics for biologists contains articles on many of the points above.

Software and code

Policy information about availability of computer code

Data collection	Structural modelling was performed using AlphaFold3.
Data analysis	1. Differential gene and protein analysis from RNAseq and proteomic experiments were performed using R Statistical Software (v4.2.2; R Core Team 2022) and the limma package (v3.54.2).<br>2. Proteomic data search was performed on Spectronaut18.<br>3. Magnetic resonance imaging data were analyzed using Analyze Direct 14.0.<br>4. Gene set enrichment analysis was performed with clusterProfiler package (v4.10.0).<br>5. Western blot densitometry quantification were performed by ImageJ 1.52q.<br>6. Statistical analyses were performed using GraphPad Prism8.<br>7. ANCOVA analysis was performed using the car and emmeans packakages in R Statistical Software.

For manuscripts utilizing custom algorithms or software that are central to the research but not yet described in published literature, software must be made available to editors and reviewers. We strongly encourage code deposition in a community repository (e.g. GitHub). See the Nature Portfolio guidelines for submitting code & software for further information.

Data

Policy information about availability of data

All manuscripts must include a data availability statement. This statement should provide the following information, where applicable:

- Accession codes, unique identifiers, or web links for publicly available datasets
- A description of any restrictions on data availability
- For clinical datasets or third party data, please ensure that the statement adheres to our policy

All data supporting the findings of this study are available in the paper and its Supplementary Information.
• Proteomics data have been deposited to the ProteomeXChange Consortium (PRIDE). Accession number: PXD054899 (expression proteomics), PXD063328 (chemoproteomics), and PXD064378 (TurboID proximity labeling proteomics).
• RNAseq data have been deposited into the GEO repository. Accession number: GSE274804.

Research involving human participants, their data, or biological material

Policy information about studies with human participants or human data. See also policy information about sex, gender (identity/presentation), and sexual orientation and race, ethnicity and racism.

Reporting on sex and gender	Adipose tissues from both male and female patients were used in this study. The sex of all individuals are reported in Supplementary Table 2.
Reporting on race, ethnicity, or other socially relevant groupings	N/A
Population characteristics	N/A
Recruitment	N/A
Ethics oversight	Rapid Autopsy Samples: Visceral adipose tissues from decedents who have previously been diagnosed with pancreatic ductal adenocarcinoma were obtained from the University of Nebraska Medical Centre's Rapid Autopsy Program (RAP) for Pancreas in compliance with IRB 091-01.<br><br>Columbia Tissue Bank resected samples: Visceral white adipose tissue was freshly collected from human patients undergoing laparoscopic cholecystectomy or resection of pancreatic ductal adenocarcinoma in compliance with IRB-AAAU7458.

Note that full information on the approval of the study protocol must also be provided in the manuscript.

Field-specific reporting

Please select the one below that is the best fit for your research. If you are not sure, read the appropriate sections before making your selection.

☒ Life sciences ☐ Behavioural & social sciences ☐ Ecological, evolutionary & environmental sciences

For a reference copy of the document with all sections, see nature.com/documents/nr-reporting-summary-flat.pdf

Life sciences study design

All studies must disclose on these points even when the disclosure is negative.

Sample size	We estimated the sample size considering the variation and mean of the samples. No statistical method was used to predetermine the sample size.
Data exclusions	No animals or samples were excluded from any analysis.
Replication	Biochemical experiments in vitro were repeated at least three times, and the repeat number was increased according to effect size or sample variation.
Randomization	Animals were randomly assigned groups for in vivo studies; no formal randomization method was applied when assigning animals for treatment.
Blinding	Investigators were blinded to group allocation during immunohistochemical data analysis.

Reporting for specific materials, systems and methods

We require information from authors about some types of materials, experimental systems and methods used in many studies. Here, indicate whether each material, system or method listed is relevant to your study. If you are not sure if a list item applies to your research, read the appropriate section before selecting a response.

Materials & experimental systems

n/a	Involved in the study
☐	☒ Antibodies
☐	☒ Eukaryotic cell lines
☒	☐ Palaeontology and archaeology
☐	☒ Animals and other organisms
☐	☒ Clinical data
☒	☐ Dual use research of concern
☒	☐ Plants

Methods

n/a	Involved in the study
☒	☐ ChIP-seq
☐	☒ Flow cytometry
☒	☐ MRI-based neuroimaging

Antibodies

Antibodies used

Primary antibodies for western blots:
4-HNE (RRID: AB_722490, 1:1000), ACTIN (RRID: AB_10998774, 1:1000), CS (RRID: AB_2665545, 1:1000), FABP4 (RRID: AB_3081454, 1:1000), FTH1 (RRID: AB_11217441, 1:1000), HSL (RRID: AB_2798800, 1:1000), HSP90 (RRID: AB_2120924, 1:1000), MSRA(RRID: AB_2804997, 1:250), MSRB2 (RRID: AB_2813685, 1:1000), MSRB3 (RRID: AB_2720052, 1:1000), MYC-TAG(RRID: AB_331783, 1:1000), pHSL-Ser660 (RRID: AB_2893315, 1:1000), PKA (RRID: AB_1523259, 1:1000), PLIN1 (RRID: AB_10829911, 1:1000), pp38-Thr180/Tyr182 (RRID: AB_2139682, 1:1000), pPKA substrates (RRID: AB_331817, 1:1000), TFR1 (RRID: AB_2904534, 1:1000), TH (RRID: AB_297840, 1:200), TUBULIN (RRID: AB_2288042, 1:1000), UCP1(RRID: AB_2241462,1:250), VINCULIN (RRID: AB_11458106, 1:2000), TNAP (RRID:AB_664062, 1:500). Affinity-purified rabbit-anti-full-length mouse Nrf2 antibody (made by JR Prigge, Montana State University) was provided by Dr. Ed Schmidt (Montana State University).

Secondary antibodies for western blots:
Peroxidase-AffiniPure Donkey Anti-Rabbit IgG (RRID: AB_10015282, 1:2500), Peroxidase-AffiniPure Donkey Anti-Mouse IgG (RRID: AB_2340770, 1:2500), AffiniPure Donkey Anti-Goat IgG (RRID: AB_2313587, 1:2500).

Antibodies for flow cytometry
Purified Anti-Mouse CD16 / CD32 (Fc Shield) (2.4G2) (RRID: AB_2621487, 1:100), Anti-CK19 Alexa Fluor 488 [EP1580Y] (RRID: AB_2927708, 1:100), Anti-CK19 Alexa Fluor 594 [EP1580Y] (No RRID assigned, 1:100), Anti-CD8 APC/Cy7 [53-6.7] (RRID: AB_312752, 1:100), Anti-Ly-6G APC [1A8] (RRID: AB_1877163, 1:100), Anti-CD25 [PC61.5] Alexa Fluor 488 (RRID: AB_763472, 1:100)
Anti-CD25 APC [PC61.5], APC (RRID: AB_469366, 1:100), Anti-FOXP3 PE [FJK-16S] (RRID: AB_465936, 1:100), Anti-IFNG Brilliant Ultraviolet 395 [XMG1.2] (RRID: AB_2925305, 1:100), Anti-CD11b FITC [M1/70] (RRID: AB_464936, 1:100), Anti-CD11b PE-eFluor780 [M1/70] (RRID: AB_2574527, 1:100), Anti-CD68 APC-eFluor780 [FA-11] (RRID: AB_2762642, 1:100), Anti-CD163 PerCP-eFLuor710 [TNKUPJ] (RRID: AB_2716955, 1:100), Anti-CD19 Super BrightTM 436 [1D3] (RRID: AB_2688107, 1:100), Anti-CD4 Brilliant Ultraviolet 615 [RM4-5] (RRID: AB_2920980, 1:100)

Primary antibodies for immunhistochemistry:
mUCP1 (RRID: AB_2687530, 1:500), mB3-AR (RRID: AB_2636362, 1:100), mTH (RRID: AB_297840, 1:250), hUCP1 (RRID: AB_2241462, 1:100), hMSRA (RRID: AB_2682032, 1:50).

Primary antibodies for immunofluorescence:
TNAP (RRID: AB_11129451, 1:25), UCP1 (AB_261838, 1:200), COX4-I1 (AB_2085286, 1:50).

Secondary antibodies for immunofluorescence:
anti-rat Alexa Fluor 488 (RRID: AB_141709, 1:100), anti-rabbit Alexa Fluor 568 (RRID: AB_2534017, 1:100), or anti-goat Alexa Fluor 647 (RRID: AB_141844, 1:100)

Validation

Antibody name, catalog number, Company name, and company validation statement:
Purified Anti-Mouse CD16 / CD32 (Fc Shield) (2.4G2) AB_2621487 Tonbo Biosciences The 2.4G2 antibody is specific for a common epitope found in the extracellular regions of mouse Fc-receptors Fc-gamma II (CD32) and Fc-gamma III (CD16).
Anti-CK19 Alexa Fluor 488 [EP1580Y] AB_2927708 Abcam A rabbit recombinant monoclonal antibody detecting Cytokeratin 19. KO validated for confirmed specificity.
Anti-CK19 Alexa Fluor 594 [EP1580Y] Abcam Rabbit Recombinant Monoclonal Cytokeratin 19 antibody - conjugated to Alexa Fluor® 594. KO validated for confirmed specificity.
Anti-CD8 APC/Cy7 [53-6.7] AB_312752 Biolegend Each lot of this antibody is quality control tested by immunofluorescent staining with flow cytometric analysis. Verified reactivity: Mouse.
Anti-Ly-6G APC [1A8] AB_1877163 Biolegend Each lot of this antibody is quality control tested by immunofluorescent staining with flow cytometric analysis. Verified reactivity: Mouse.
Anti-CD25 [PC61.5] Alexa Fluor 488 AB_763472 Invitrogen Applications Tested: This PC61.5 antibody has been tested by flow cytometric analysis of mouse splenocytes. Verified reactivity: Mouse.
Anti-CD25 APC [PC61.5], APC AB_469366 Invitrogen Applications Tested: The PC61.5 antibody has been tested by flow cytometric analysis of mouse splenocytes. Verified reactivity: Mouse.
Anti-FOXP3 PE [FJK-16S] AB_465936 Invitrogen Applications Tested: This FJK-16s antibody has been tested by intracellular flow cytometric analysis of mouse splenocytes using the Foxp3/Transcription Factor Staining Buffer Set (Product # 00-5523-00) and protocol. Verified reactivity: Mouse.
Anti-IFNG Brilliant Ultraviolet 395 [XMG1.2] AB_2925305 Invitrogen This XMG1.2 antibody has been tested by intracellular staining

followed by flow cytometric analysis of mouse splenocytes using the Intracellular Fixation & Permeabilization Buffer Set (Product # 88-8824-00) and protocol. Verified reactivity: Mouse.
Anti-CD11b FITC [M1/70] AB_464936 eBiosceince Applications Tested: Has been tested by flow cytometric analysis of mouse splenocytes and bone marrow cells. This can be used at less than or equal to 0.5 µg per test. Verified reactivity: Mouse.
Anti-CD11b PE-eFluor780 [M1/70] AB_2574527 eBiosceince Applications Tested: This M1/70 antibody has been tested by flow cytometric analysis of mouse bone marrow cells. Verified reactivity: Mouse.
Anti-CD68 APC-eFluor780 [FA-11] AB_2762642 eBiosceince Applications Tested: This FA-11 antibody has been tested by intracellular staining and flow cytometric analysis of mouse bone marrow derived. Verified reactivity: Mouse.
Anti-CD163 PerCP-eFLuor710 [TNKUPJ] AB_2716955 eBiosceince Applications Tested: This TNKUPJ antibody has been tested by flow cytometric analysis of mouse splenocytes. Verified reactivity: Mouse.
Anti-CD19 Super BrightTM 436 [1D3] AB_2688107 eBiosceince Applications Tested: This eBio1D3 (1D3) antibody has been tested by flow cytometric analysis of mouse splenocytes. Verified reactivity: Mouse.
Anti-CD4 Brilliant Ultraviolet 615 [RM4-5] AB_2920980 eBiosceince This RM4-5 antibody has been tested by flow cytometric analysis of mouse splenocytes. Verified reactivity: Mouse.
TH AB_297840 Abcam Rabbit Polyclonal Tyrosine Hydroxylase antibody. Neural Stem Cell marker.
pp38 AB_2139682 Cell Signaling Phospho-p38 MAPK (Thr180/Tyr182) (D3F9) XP® Rabbit mAb detects endogenous levels of p38 MAPK only when phosphorylated at Thr180 and Tyr182. This antibody does not cross-react with the phosphorylated forms of either p42/44 MAPK or SAPK/JNK.
HSP90 AB_2120924 Protein tech HSP90 Antibody detects endogenous levels of total HSP90 protein, alpha and beta isoforms. This antibody does not cross-react with other HSPs.
VINCULIN AB_11458106 Cell Signaling Vinculin (E1E9V) XP® recognizes endogenous levels of total vinculin protein. This antibody also reacts with metavinculin, a 145 kDa splice variant of vinculin.
UCP1 AB_2241462 Abcam UCP1 antibody recognizes endogenous levels of total UCP1 protein. This antibody does not cross-react with UCP2 and UCP3 proteins.
MSRA AB_1853826 Sigma MSRA antibody recognizes endogenous levels of MSRA wuth band of predicted size in kDa (+/-20%) with additional bands present.
TFR1 AB_2904534 Abcam TFR1 recognizes endogenous levels of Transferrin Receptor.
PLIN1 AB_10829911 Cell Signaling Perilipin-1 (D1D8) XP® Rabbit mAb detects endogenous levels of total perilipin-1 protein.
MYC-TAG AB_331783 Cell Signaling Myc-Tag (9B11) Mouse mAb detects exogenously expressed proteins containing the Myc epitope tag. This antibody recognizes the Myc tag fused to either the amino or carboxy terminus of targeted proteins in transfected cells. Myc-Tag (9B11) Mouse mAb detects exogenously expressed Myc-tagged proteins in cells expressed under a CMV promoter. Expression under other promoters has not been evaluated.
PPKA SUBSTRATE AB_331817 Cell Signaling Phospho-PKA Substrate (RRXS*/T*) (100G7E) Rabbit mAb detects peptides and proteins containing a phospho-Ser/Thr residue with arginine at the -3 and -2 positions. It is a useful tool in identifying new substrates of PKA. The antibody recognizes other -3 arginine-bearing phospho-Ser/Thr peptides, such as substrate motifs for Akt and PKC, to a lesser extent. It does not recognize the nonphosphorylated substrate motif peptides. (U.S. Patent No's.: 6,441,140; 6,982,318; 7,259,022; 7,344,714; U.S.S.N. 11,484,485; and all foreign equivalents.)
pHSL(Ser660) AB_2893315 Cell Signaling Phospho-HSL (Ser660) Antibody detects endogenous levels of HSL only when phosphorylated at Ser660 by PKA. This antibody does not cross-react with Ser563, Ser565, or Ser659 phosphorylated HSL.
HSL AB_2798800 Cell Signaling HSL (D6W5S) XP® Rabbit mAb recognizes endogenous levels of total HSL protein.
CS AB_2665545 Cell Signaling Citrate Synthase (D7V8B) Rabbit mAb recognizes endogenous levels of total citrate synthase protein.
MSRB3 AB_2720052 ThermoFisher MSRB3 antibody recognizes Recombinant protein of human MSRB3.
Tubulin AB_2288042 Cell Signaling The α/β-Tubulin Antibody detects endogenous levels of both α- and β-tubulin total protein, and does cross-react with both recombinant α- and β-tubulin.
4-HNE AB_722490 Abcam This polyclonal antibody targets 4 Hydroxynonenal.
FABP4 AB_3082382 Boster Bio FABP4 Antibody detects endogenous levels of total FABP4 protein.
FTH1 AB_11217441 Cell Signaling FTH1 (D1D4) Rabbit mAb recognizes endogenous levels of total FTH1 protein.
PKA AB_1523259 Abcam Rabbit Recombinant Monoclonal cAMP Protein Kinase Catalytic subunit phospho T197 + T197 + T197 + T197 + T197 antibody.
MSRB2 AB_2813685 ThermoFisher MSRB2 antibody recognizes Recombinant protein of human MSRB2.
Actin AB_10998774 Cell Signaling Pan-Actin (D18C11) Rabbit mAb recognizes endogenous levels of total actin protein (all isoforms).
TNAP AB_11129451 R&D Systems Antibody detects human Alkaline Phosphatase/ALPL in direct ELISAs. In direct ELISAs, 80-100% cross-reactivity with recombinant mouse Alkaline Phosphatase/ALPL is observed and no cross-reactivity with recombinant human (rh) Alkaline Phosphatase/ALPP or rhAlkaline Phosphatase/ALPI is observed.
UCP1 AB_261838 Sigma Aldrich Anti-UCP-1 specifically recognizes human, mouse and rat UCP-1. Staining of the UCP-1 band is specifically inhibited with the immunizing peptide. The epitopes recognized are compatible with routine formalin-fixation and paraffin-embedding.
COX4-I1 AB_2085286 R&D Systems Antibody detects human and mouse COX-4I1 in Western blots.

Peroxidase-AffiniPure Donkey Anti-Rabbit IgG AB_10015282 Jackson Immuno Research Based on immunoelectrophoresis and/or ELISA, the antibody reacts with whole molecule rabbit IgG. It also reacts with the light chains of other rabbit immunoglobulins. No antibody was detected against non-immunoglobulin serum proteins. The antibody has been tested by ELISA and/or solid-phase adsorbed to ensure minimal cross-reaction with bovine, chicken, goat, guinea pig, syrian hamster, horse, human, mouse, rat and sheep serum proteins, but it may cross-react with immunoglobulins from other species.
Peroxidase-AffiniPure Donkey Anti-Mouse IgG AB_2340770 Jackson Immuno Research Based on immunoelectrophoresis and/or ELISA, the antibody reacts with whole molecule mouse IgG. It also reacts with the light chains of other mouse immunoglobulins. No antibody was detected against non-immunoglobulin serum proteins. The antibody has been tested by ELISA and/or solid-phase adsorbed to ensure minimal cross-reaction with bovine, chicken, goat, guinea pig, syrian hamster, horse, human, rabbit and sheep serum proteins, but it may cross-react with immunoglobulins from other species.

Eukaryotic cell lines

Policy information about cell lines and Sex and Gender in Research

Cell line source(s)	1. HEK293T cell line was from ATCC (Cat# CRL-3216).<br>2. Primary white adipocytes were isolated from newborn mice<br>3. Primary PDAC tumor cell line was isolated from tumor of KPC mouse
Authentication	Identity of HEK293T is verified by STR profiling on a periodic basis (Duke University Core Facility). Identity of primary adipocytes is authenticated by adipocyte specific marker expression, including FABP4, and perilipins. Mouse pancreatic cancer cell lines used for transplantation are genotyped to ensure they carry mutant alleles of Kras and Trp53. Cells cultured in our laboratory are screened for mycoplasma contamination monthly and authenticated by short tandem repeat (STR) analysis to identify misidentified, cross-contaminated, or genetically drifted cells.
Mycoplasma contamination	Cell lines are tested for mycoplasma contamination quarterly by nested PCR assay.
Commonly misidentified lines<br>(See ICLAC register)	N/A

Animals and other research organisms

Policy information about studies involving animals; ARRIVE guidelines recommended for reporting animal research, and Sex and Gender in Research

Laboratory animals	Genetically engineered mouse model of pancreatic cancer: Trp53+/LSL-R172H, Kras+/LSL-G12D, Pdx1-Cre, strains in C57Bl/6NJ background were interbred to (KPC) mice. MsrA knockout mice in C57BL/6NJ were obtained from Jackson Laboratory (RRID:MMRRC_050602-JAX) and interbred with KPC to generate compound mutants obtain Kras+/LSL-G12D; Trp53+/LSL-R172H ; Pdx1-Cre; MsrA–/– (KPCM).<br><br>C57Bl/6J mice were purchased from the Jackson Laboratory (RRID:IMSR_JAX:000664) for syngeneic orthotopic transplant experiments.<br><br>All animal experiments were conducted in accordance with procedures approved by the IACUC at Columbia University (AC-AABK5554). The tumour burden endpoints (tumour volume no greater than 2,000mm3; morbidity; laboured breathing; impingement of the animal's ability to ambulate, eat, and drink) allowed by our IACUC were not exceeded.
Wild animals	N/A
Reporting on sex	Sex balanced cohorts of male and female KPC and KPCM mice were used in the study and reported in relevant figures.<br><br>Males were used for syngeneic orthotopic transplant experiments upon establishing no sexual dimorphism in the phenotype reported.
Field-collected samples	N/A
Ethics oversight	All animal experiments were conducted in accordance with procedures approved by the IACUC at Columbia University (AC-AABK5554).

Note that full information on the approval of the study protocol must also be provided in the manuscript.

Clinical data

Policy information about clinical studies

All manuscripts should comply with the ICMJE guidelines for publication of clinical research and a completed CONSORT checklist must be included with all submissions.

Clinical trial registration	N/A
Study protocol	Human Patient Samples<br>Rapid Autopsy Samples: Visceral adipose tissues from decedents who have previously been diagnosed with pancreatic ductal adenocarcinoma were obtained from the University of Nebraska Medical Centre's Rapid Autopsy Program (RAP) for Pancreas in compliance with IRB 091-01.<br><br>Columbia Tissue Bank resected samples: Visceral white adipose tissue was freshly collected from human patients undergoing laparoscopic cholecystectomy or resection of pancreatic ductal adenocarcinoma in compliance with IRB-AAAU7458.
Data collection	N/A
Outcomes	N/A

Plants

Seed stocks	N/A
Novel plant genotypes	N/A
Authentication	N/A

Flow Cytometry

Plots

Confirm that:

☐ The axis labels state the marker and fluorochrome used (e.g. CD4-FITC).

☐ The axis scales are clearly visible. Include numbers along axes only for bottom left plot of group (a 'group' is an analysis of identical markers).

☐ All plots are contour plots with outliers or pseudocolor plots.

☐ A numerical value for number of cells or percentage (with statistics) is provided.

Methodology

Sample preparation	*Describe the sample preparation, detailing the biological source of the cells and any tissue processing steps used.*
Instrument	*Identify the instrument used for data collection, specifying make and model number.*
Software	*Describe the software used to collect and analyze the flow cytometry data. For custom code that has been deposited into a community repository, provide accession details.*
Cell population abundance	*Describe the abundance of the relevant cell populations within post-sort fractions, providing details on the purity of the samples and how it was determined.*
Gating strategy	*Describe the gating strategy used for all relevant experiments, specifying the preliminary FSC/SSC gates of the starting cell population, indicating where boundaries between "positive" and "negative" staining cell populations are defined.*

☐ Tick this box to confirm that a figure exemplifying the gating strategy is provided in the Supplementary Information.
