# Supplementary material for: An iron-regulated methionine redox axis governs adipose browning and cancer cachexia
Source: Nat Cancer. 2026 Sep 22;7(10):1514–32. doi: 10.1038/s43018-026-01234-y (PMC13694233; doi:10.1038/s43018-026-01234-y)

Extended Data Figure 7 raw gel images

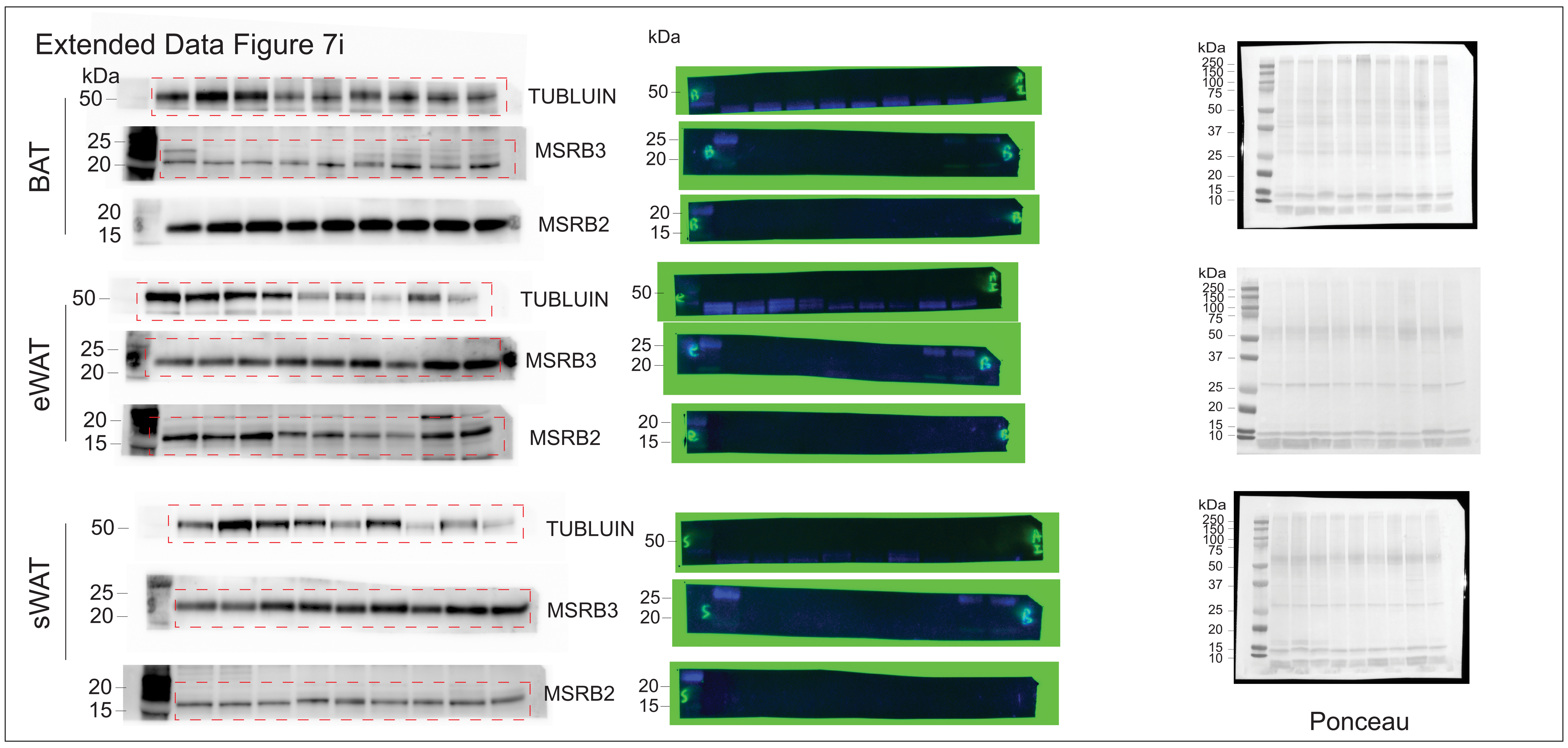

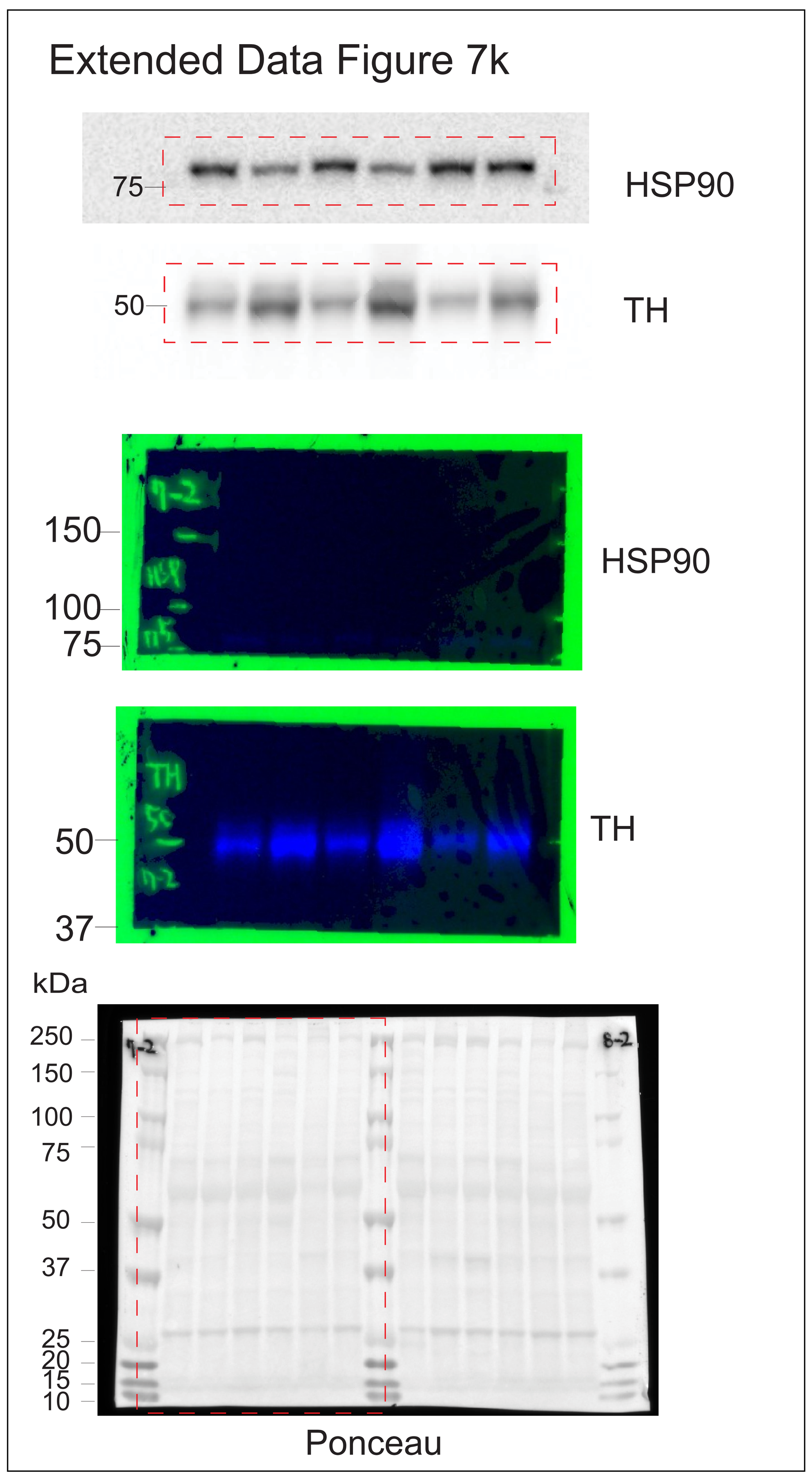

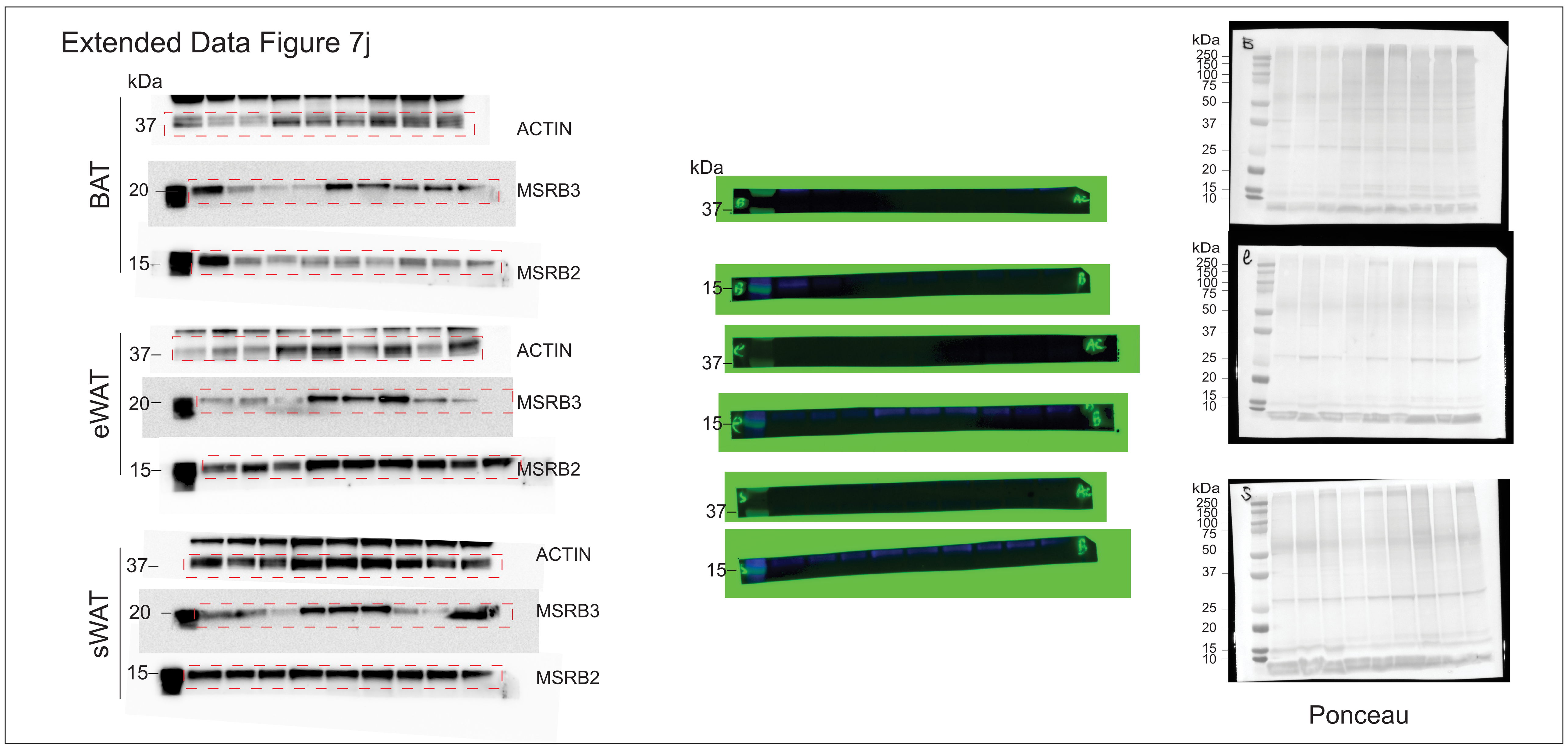

Supplement: Supplementary file 9 [file 43018_2026_1234_MOESM25_ESM.pdf]
